# PD-1 antibody camrelizumab plus apatinib and SOX as first-line treatment in patients with AFP-producing gastric or gastro-esophageal junction adenocarcinoma (CAP 06): a multi-center, single-arm, phase 2 trial

**DOI:** 10.1038/s41392-025-02193-z

**Published:** 2025-03-14

**Authors:** Yakun Wang, Jialin Lu, Xiaoyi Chong, Chang Wang, Xiaofeng Chen, Zhi Peng, Yanhong Gu, Yizhuo Wang, Xicheng Wang, Jian Li, Jifang Gong, Changsong Qi, Jiajia Yuan, Zhihao Lu, Ming Lu, Jun Zhou, Yanshuo Cao, Yang Chen, Cheng Zhang, Zhiguo Hou, Hongyi Kou, Lin Shen, Xiaotian Zhang

**Affiliations:** 1 State Key Laboratory of Holistic Integrative Management of Gastrointestinal Cancers, Beijing Key Laboratory of Carcinogenesis and Translational Research, Department of Gastrointestinal Oncology, Peking University Cancer Hospital & Institute, Beijing, China; 2 Key Laboratory of Carcinogenesis and Translational Research (Ministry of Education/Beijing), Department of Gastrointestinal Oncology, Peking University Cancer Hospital & Institute, Beijing, China; 3 Cancer Center, The First Hospital of Jilin University, Changchun, China; 4 Department of Oncology, The First Affiliated Hospital of Nanjing Medical University, Nanjing, China; 5 Jiangsu Key Lab of Cancer Biomarkers, Prevention and Treatment, Collaborative Innovation Center for Cancer Personalized Medicine, Nanjing Medical University, Nanjing, China; 6 Gastric Cancer Center, The First Affiliated Hospital of Nanjing Medical University, Nanjing, China; 7 State Key Laboratory of Holistic Integrative Management of Gastrointestinal Cancers, Beijing Key Laboratory of Carcinogenesis and Translational Research, Department of Early Drug Development Center, Peking University Cancer Hospital & Institute, Beijing, China; 8 Jiangsu Hengrui Pharmaceuticals Co. Ltd, Shanghai, China; 9 Department of Gastrointestinal Oncology, Peking University Cancer Hospital (Inner Mongolia Campus)/Affiliated Cancer Hospital of Inner Mongolia Medical University, Hohhot, China

**Keywords:** Gastrointestinal cancer, Biomarkers

## Abstract

Alpha-fetoprotein-producing gastric or gastro-esophageal junction (AFP-G/GEJ) cancer, a rare gastric cancer subtype, exhibits increased angiogenesis and more immunosuppression than non-AFP-G/GEJ cancer. The potential benefits of anti-angiogenic agents and immunotherapy for this specific subtype remain unknown. This multi-center, single-arm, phase 2 trial (ClinicalTrials.gov NCT04609176) evaluated the antitumor activity, safety, and biomarkers of camrelizumab plus apatinib and S-1 and oxaliplatin (SOX), followed by maintenance treatment with camrelizumab plus apatinib, as a first-line treatment in patients with AFP-G/GEJ adenocarcinoma. Primary endpoint was the confirmed objective response rate (ORR) per RECIST v1.1 in the full analysis set. Secondary endpoints were disease control rate (DCR), progression-free survival (PFS), overall survival (OS), duration of response, time to response, and safety. Between December 4, 2020, and August 4, 2023, 36 patients were enrolled and treated. The trial met its primary endpoint with a confirmed ORR of 66.7% (95% CI: 49.0–81.4). The DCR was 88.9% (95% CI: 73.9-96.9). With a median follow-up of 11.7 months (range: 3.2-37.9), the median PFS reached 7.8 months (95% CI: 4.9-12.3) and the median OS reached 18.0 months (95% CI: 10.5-NR). No new safety concerns were identified. In exploratory analysis, patients with durable clinical benefit exhibited higher pre-treatment (PD-1^+^) CD8^+^ T cell densities and effective scores. First-line treatment with camrelizumab plus apatinib and SOX, followed by maintenance treatment with camrelizumab plus apatinib, is effective and safe in AFP-G/GEJ adenocarcinoma. Further studies are necessary to validate these findings.

## Introduction

Alpha-fetoprotein-producing gastric cancer (AFP-GC) is a rare but aggressive subtype of gastric cancer (GC), marked by increased serum alpha-fetoprotein (AFP) levels and AFP positivity on immunohistochemical (IHC) analysis. This subtype accounts for 1.3% to 15% of all GC cases.^1^ AFP-GC patients have poor prognosis and exhibit histopathological features of hepatoid adenocarcinoma.^2^ Currently, no specific treatments for AFP-GC exist, and standard management follows guidelines for general GC, including first-line chemoimmunotherapy.^3^ Data indicate that GC patients with high AFP levels have poor outcomes after receiving programmed cell death 1 (PD-1) inhibitors plus chemotherapy.^4^ New treatments are crucially needed to enhance outcomes for AFP-GC patients.

Increased angiogenesis was observed in AFP-GC patients compared with non-AFP-GC.^5^ The anti-VEGFR2 agent, ramucirumab, has shown improved antitumor activity in AFP-GC patients compared to non-AFP-GC.^6^ Apatinib, another anti-VEGFR2 agent, has also demonstrated antitumor activity in AFP-GC patients.^7^ Additionally, AFP-GC patients have more immunosuppressive tumor microenvironment (TME), with a significant reduction in CD8 + T cell infiltration.^8^ As angiogenesis is a key determinant of CD8 + T cells infiltration and function in TME,^9^ the combination of anti-angiogenic drugs and PD-1 inhibitors has shown promise. This combination can synergistically enhance immune response and reverse resistance to immunotherapy by normalizing vascular-immune crosstalk.^10^ In preclinical models, AFP overexpression reduced the response to PD-1 antibody, and the addition of apatinib overcame resistance to PD-1 inhibitor.^11^ Thus, we presume that anti-VEGFR combination treatments might be particularly beneficial for AFP-GC.

Recent phase 2 trials have reported that combining anti-VEGFR agents with PD-1 inhibitors and chemotherapy (lenvatinib plus pembrolizumab and chemotherapy, and regorafenib plus nivolumab and chemotherapy) resulted in beneficial outcomes in gastric or gastro-esophageal junction (G/GEJ) cancer patients.^12,13^ Additionally, camrelizumab in combination with S-1 and oxaliplatin (SOX) plus apatinib or with capecitabine and oxaliplatin (CAPOX), then maintained with camrelizumab and apatinib have yielded promising objective response rate (ORR), progression-free survival (PFS) and tolerable adverse events in G/GEJ cancer patients.^14,15^ Nevertheless, clinical trial data for such a combination in AFP-GC patients remain scarce.

Camrelizumab (a PD-1 inhibitor) combined with apatinib (a VEGFR2-targeted tyrosine kinase inhibitor) has received approval in China as the first-line therapy for hepatocellular carcinoma. Apatinib monotherapy has granted approval in China as the third-line or above therapy for GC. For G/GEJ cancer patients, 250 mg daily of apatinib was previously recommended when administered with camrelizumab, and a higher dose of 375 mg increased adverse events (AEs).^16^ This dosage was also commonly used when apatinib was administered with camrelizumab and chemotherapy in patients with GC, esophageal cancer, or other solid tumors.^15,17–19^ In this single-arm trial, we selected an apatinib dose of 250 mg daily. Since data on the safety of combining anti-VEGFR agents with immunochemotherapy were limited at the time of study design, we chose 4 cycles of chemotherapy to reduce the potential toxicity risk associated with the combination. This trial aimed to assess the antitumor activity, safety, and predictive biomarkers of camrelizumab combined with apatinib and chemotherapy in previously untreated patients with AFP-producing G/GEJ (AFP-G/GEJ) adenocarcinoma.

## Results

### Patient characteristics

**Fig. 1:**
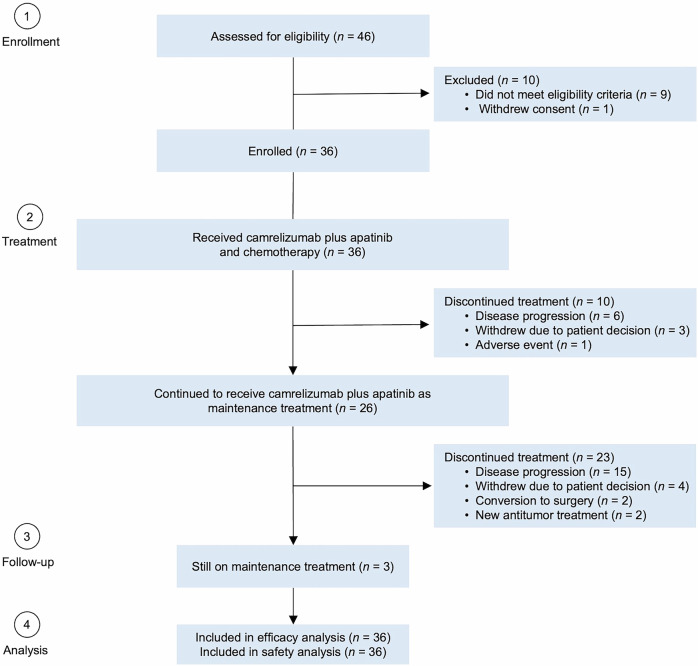
Patient flowchart

From December 4, 2020, to August 4, 2023, 36 patients were enrolled and all received camrelizumab plus apatinib and SOX. Of these, 10 patients discontinued combination treatment due to disease progression (n = 6), withdrawal of consent (n = 3), and adverse events (n = 1). Consequently, 26 patients (72.2%) continued to receive camrelizumab plus apatinib as maintenance treatment (Fig. 1). Antitumor activity and safety were analyzed in all 36 patients. The data cutoff for safety and efficacy analysis was March 27, 2024. Following the end of study treatment, 21 patients (58.3%) received at least one subsequent antitumor treatment (supplementary Table 1).

The median age was 63 years (range, 28-78), and 30 patients (83.3%) were male. Most patients had gastric cancer (n = 23, 63.9%) and were in stage IVB (n = 31, 86.1%). In addition, 18 patients (50.0%) had liver metastasis, 17 (47.2%) had distant lymph node metastasis, and 9 (25.0%) had peritoneal metastasis. Median serum AFP level was 739.8 ng/ml (range: 77.7-321,847.0). Programmed cell death ligand 1 (PD-L1) expression was observed as CPS ≥ 1 in 18 patients (50.0%), CPS ≥ 5 in 11 patients (30.6%), and CPS ≥ 10 in 5 patients (13.9%). The baseline characteristics were summarized in Table 1.

**Table 1:** Baseline characteristics

Characteristics	Patients (n = 36)
Age, years	63 (28-78)
Male	30 (83.3)
ECOG performance status	
0	20 (55.6)
1	15 (41.7)
2	1 (2.8)
Primary tumor location	
Gastric	23 (63.9)
GEJ	13 (36.1)
Lauren’s classification	
Intestinal	18 (50.0)
Diffuse	6 (16.7)
Mixed	7 (19.4)
Unknown	5 (13.9)
Clinical stage	
III	2 (5.6)
IVA	3 (8.3)
IVB	31 (86.1)
Prior gastrectomy	6 (16.7)
Prior radiotherapy	0
Organs with metastases	
1 or 2	30 (83.3)
>2	6 (16.7)
Metastasis sites	
Liver	18 (50.0)
Distant lymph nodes	17 (47.2)
Peritoneum	9 (25.0)
PVTT	4 (11.1)
Lung	1 (2.8)
Serum AFP level^a^	739.8 (77.7, 321847.0)
≥400 ng/ml	22 (61.1)
≥800 ng/ml	18 (50.0)
Mismatch repair status	
pMMR	34 (94.4)
Unknown	2 (5.6)
Epstein-Barr virus status	
Negative	32 (88.9)
Unknown	4 (11.1)
PD-L1 expression	
CPS ≥ 1	18 (50.0)
CPS ≥ 5	11 (30.6)
CPS ≥ 10	5 (13.9)
Unknown	4 (11.1)

Data are shown as n (%) or median (range)

*ECOG* Eastern Cooperative Oncology Group, *GEJ* gastroesophageal junction, *PVTT* portal vein tumor thrombus, *AFP* alpha-fetoprotein, *pMMR* proficient mismatch repair, *PD-L1* programmed cell death ligand 1, *CPS* combined positive score

^a^There was only one patient had AFP < 160 ng/ml

### Efficacy

**Fig. 2:**
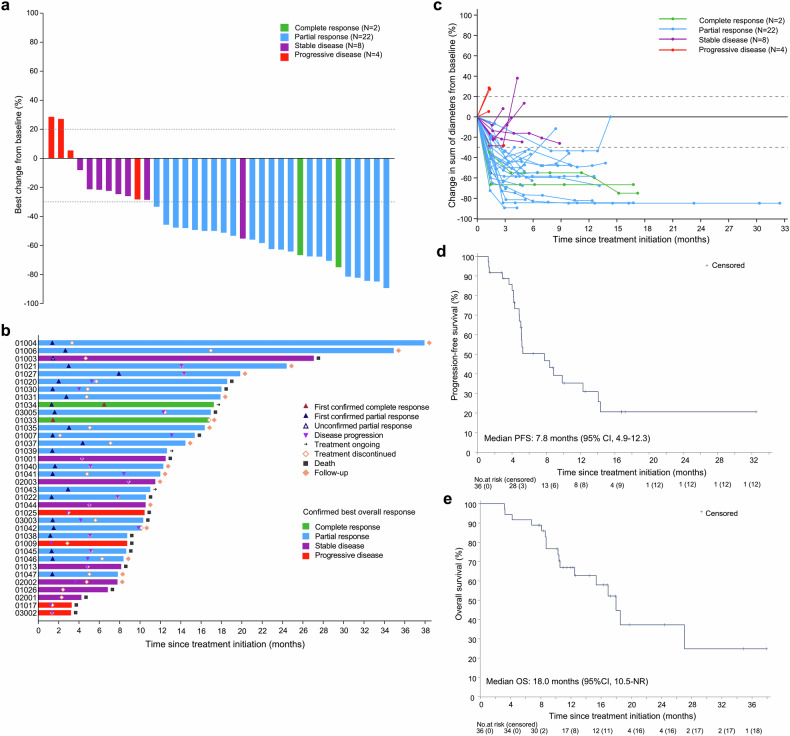
Antitumor response and survival outcomes. **a** Waterfall plot showing the percentage change in the sum of target lesion diameters from baseline. **b** Swimmer plot showing treatment exposure and response duration. Patients 1004 and 1047 had partial response, and subsequently underwent surgery. Both patients had R0 resection, with NCCN tumor regression grades of 1 and 3, respectively. As of the data cut-off date, both patients had no recurrence or death. **c** Spider plot showing the percentage change in the sum of target lesion diameters during treatment. Tumor response per RECIST v1.1 was evaluable in all patients. **d** Kaplan-Meier curve of progression-free survival. **e** Kaplan-Meier curve of overall survival

In the full analysis set, the overall confirmed ORR per RECIST v1.1 was 66.7% (95% CI: 49.0-81.4). The disease control rate (DCR) was 88.9% (95% CI: 73.9-96.9). Two patients had complete response (CR), 22 had partial response (PR), and 8 had stable disease (SD) (Table 2). The maximal tumor shrinkage of target lesion relative to baseline is depicted in a waterfall plot (Fig. 2a). Notably, all SD patients and one PD patient had tumor shrinkage from baseline, with one patient with unconfirmed PR (recorded as SD) showing 55.3% shrinkage. Treatment durations and tumor response are shown in swimmer and spider plots (Fig. 2b, c). The median time to response (TTR) was 1.4 months (95% CI: 1.4-2.0). The median duration of response (DoR) was 8.4 months (95% CI: 3.9-not reached [NR]).

**Table 2:** Antitumor response

	Patients (n = 36)
Confirmed best overall response	
Complete response	2 (5.6)
Partial response	22 (61.1)
Stable disease	8 (22.2)
Progressive disease	4 (11.1)
Confirmed objective response rate	24 (66.7)
95% CI	49.0–81.4
Disease control rate	32 (88.9)
95% CI	73.9–96.9
Time to response, months	1.4 (1.4–2.0)
Duration of response, months	8.4 (3.9–NR)

Data are shown as n (%) or median (95% CI). Tumor response per RECIST v1.1 was evaluable in all patients

During a median follow-up of 11.7 months (range: 3.2-37.9), 23 events (22 progressors and one death) occurred for PFS and 17 events for overall survival (OS). The median PFS was 7.8 months (95% CI: 4.9-12.3) (Fig. 2d). The 9-month and 12-month PFS rates were 39.2% (95% CI: 22.0-56.0) and 35.3% (95% CI: 18.7-52.3), respectively. The median OS was 18.0 months (95% CI: 10.5-NR) (Fig. 2e). The 9-month and 12-month OS rates were 76.7% (95% CI: 58.7-87.6) and 67.0% (95% CI: 48.2-80.3), respectively.

Subgroup analysis of ORR and PFS are shown in the supplementary Fig. 1 and supplementary Table 2. In patients with CPS ≥ 1, the ORR was 72.2%, compared to 71.4% in those with CPS < 1. In patients with CPS ≥ 5, the ORR was 81.8%, compared to 66.7% in those with CPS < 5. ORRs were similar across different AFP levels, with cutoffs at 400 or 800 ng/ml. Patients with GEJ tumor location, intestinal type, or with 1-2 metastatic organs showed a trend toward higher ORRs.

### Safety

Any grade treatment-related adverse events (TRAEs) were observed in 34 patients (94.4%) (supplementary Table 3). And 16 patients (44.4%) had grade ≥3 TRAEs, with decreased neutrophil count (6 [16.7%]), hypertension (3 [8.3%]), anemia (2 [5.6%]), decreased platelet count (2 [5.6%]), and diarrhea (2 [5.6%]) being the most frequently occurring events. No treatment-related deaths occurred. Any grade immune-related adverse events (irAEs) were reported in 18 patients (50.0%). The most frequent irAEs were RCCEP, increased amylase level, and hyperthyroidism (supplementary Table 3). Grade ≥3 irAEs occurred in 3 patients (8.3%), including diarrhea, decreased platelet count, and increased lipase level (n = 1 each). A detailed summary of TRAEs and irAEs is available in the supplement. Treatment-related serious adverse events (SAEs) were reported in 3 patients (8.3%), including diarrhea (2 [5.6%]), vomiting (one [2.8%]) and hyponatremia (one [2.8%]).

No patient discontinued all study drugs due to TRAEs. Three (8.3%) patients discontinued any study drug: one patient discontinued chemotherapy due to decreased neutrophil, platelet, and white blood cell counts; one patient discontinued camrelizumab due to decreased platelet count; and one patient discontinued apatinib due to fatigue. Thirteen (36.1%) patients required dose interruption or delay due to TRAEs, and 8 (22.2%) patients required dose reduction due to TRAEs. Details of dose interruption, delay, or reduction are shown in the supplementary Table 4.

### Exploratory outcomes

**Fig. 3:**
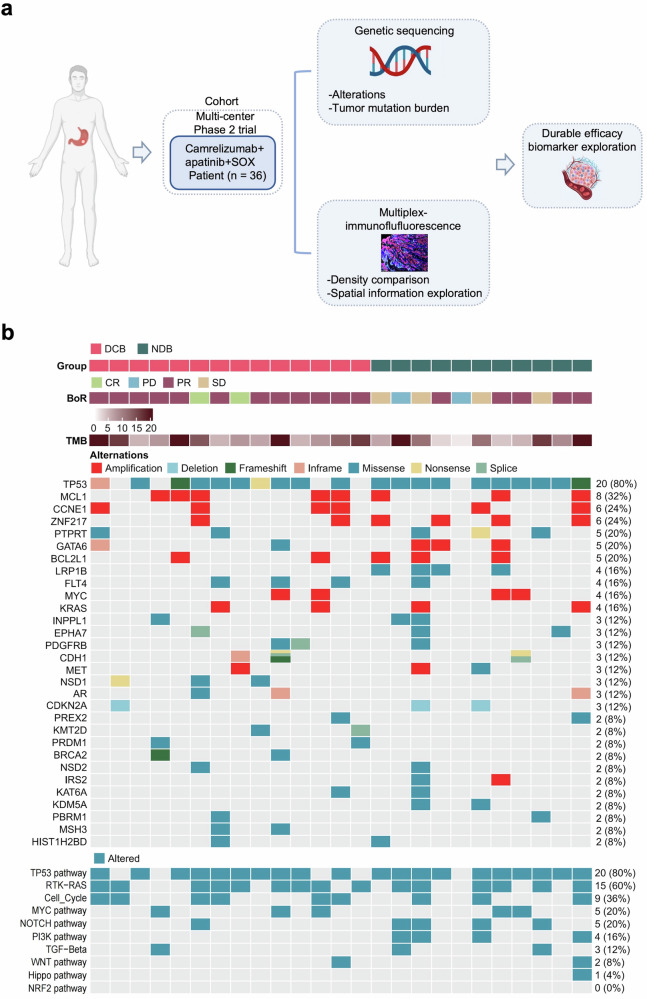
Exploratory study overview and genomic characteristic in study cohort. **a** Schematic summary of the design of exploratory analyses. **b** Gene sequencing of 25 patients with available pre-treatment tumor tissue from the study cohort

To identify potential biomarkers predictive of durable clinical response and further elucidate the molecular features of AFP-G/GEJ adenocarcinoma, targeted exome next-generation sequencing (NGS) and multiplex immunofluorescence (mIF) were performed (Fig. 3a). Patients with CR, PR, and SD lasting at least 6 months were classified in the durable clinical benefit (DCB) group. Patients with PD or PR and SD lasting less than 6 months were classified in the no durable benefit (NDB) group.

#### Genomic alterations

Gene sequencing with qualified tumor samples and matched normal tissues was performed in 25 patients (Fig. 3b). The most frequent disease-related somatic alterations were TP53 (80%), MCL1 (32%), CCNE1 (24%), and ZNF217 (24%). The frequency of TP53 mutation was significantly higher compared to both TCGA-STAD all-stage samples and TCGA-STAD advanced-stage samples only, while the frequency of mutation in ARID1A was significantly lower (supplementary Fig. 2a, b), which was consistent with previous studies.^20^ Further, we observed that LRP1B mutation and PI3K pathway alteration were only present in the NDB group (Fig. 3b; supplementary Fig. 3a, b), which revealed that LRP1B mutation and PI3K pathway alteration were significantly associated with NDB, i.e., poorer PFS. However, our cohort did not find any association between tumor mutation burden (TMB), copy number variants (CNV) burden, and clinical efficacy (supplementary Fig. 3c).

#### Association between the density of tumor-infiltrating cells and durable clinical benefit

**Fig. 4:**
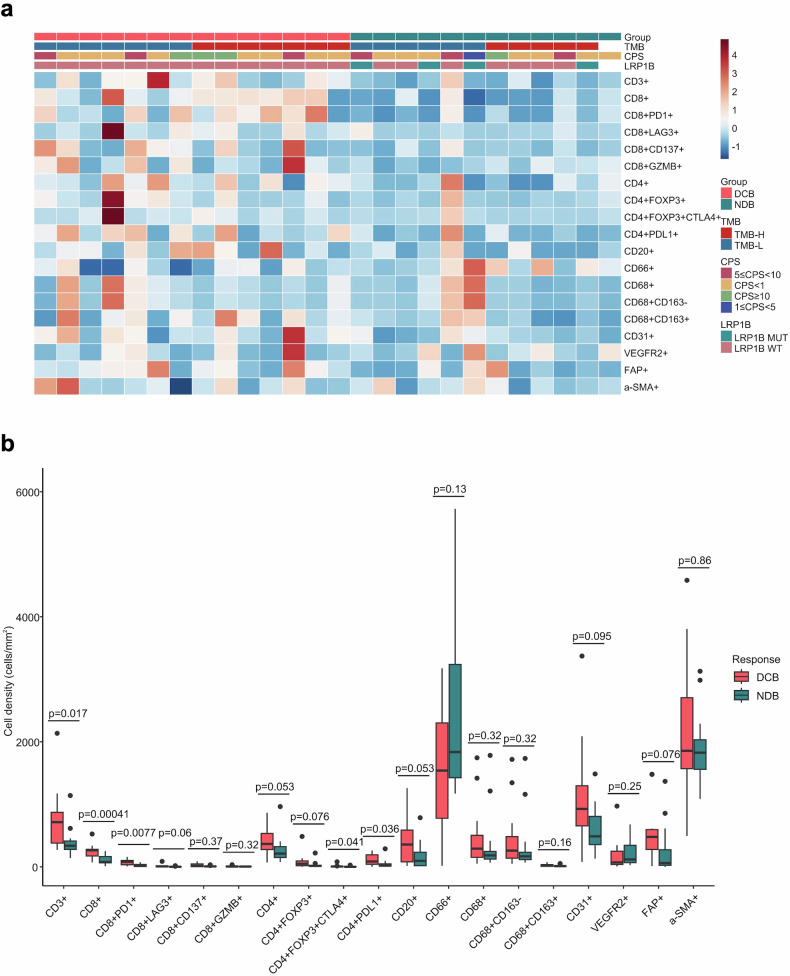
Overall TICs landscapes and comparison of TICs density between DCB and NDB groups. **a** TICs density grouped by subtypes. **b** Relationship of durable clinical benefit with TICs infiltration (DCB, n = 14; NDB, n = 12). *P* values were calculated using the two-sided Mann-Whitney U test. The line in the middle of the box represents the median. The lower and the upper edges of the box are the 1st and 3rd quartiles, respectively. The dots are considered outliers, which are more than 1.5*IQR beyond the lower or upper quartiles

We conducted a 4-panel mIF assay to provide a comprehensive and systematic presentation of the overall AFP-G/GEJ adenocarcinoma TME landscape (Fig. 4a; supplementary Fig. 4-7). The relationship between durable clinical efficacy and tumor-infiltrating cells (TICs) density was analyzed. We observed that patients with DCB had higher CD3^+^ T, CD4^+^ T and CD8^+^ T cells densities than patients with NDB (Fig. 4b). Further analyses revealed that immune checkpoints, including PD-1^+^ cells, FOXP3^+^ cells (supplementary Fig. 8a), and conventional immune checkpoint-positive T cells, such as PD-1^+^CD8^+^ T cells, PD-L1^+^CD4^+^ T cells, and CD4^+^FOXP3^+^CTLA4^+^ T cells, were significantly enriched in the DCB group (Fig. 4b). The cell density in the cohort was classified as “high” for densities falling within the top third of the group, and “low” for densities within the bottom third. A co-analysis of PD-1^+^ (CD8^+^) and FOXP3^+^ (CD4^+^) cells revealed that the proportion of patients exhibiting co-high expression of both FOXP3^+^ (CD4^+^) and PD-1^+^ (CD8^+^) cells, defined as (FOXP3^+^CD4^+^)^hi^(PD-1 + CD8 + )^hi^ or FOXP3^hi^PD-1^hi^, was higher in those who demonstrated DCB compared to the non-co-high expression group (83.3% vs 45.0, p = 0.170). Additionally, when patients with low expression of both markers, categorized as (FOXP3^+^CD4^+^)^low^(PD-1^+^CD8^+^)^low^ or FOXP3^low^PD-1^low^, were analyzed together, the proportion of DCB was 28.6% in those with (FOXP3^+^CD4^+^)^low^(PD-1^+^CD8^+^)^low^ (28.6% vs. 83.3%, p = 0.0079) and 21.4% in those with FOXP3^low^PD-1^low^ (21.4% vs. 91.7%, p = 0.0005) (supplementary Fig. 8b). Then, we compared the density of myeloid and stromal cells between the DCB and NDB groups, but no differences were observed (Fig. 4b). Tertiary lymphoid structures (TLSs) are key components of the TME,^21,22^ and we evaluated the predictive value of TLSs for ICIs-combined therapy in patients with AFP-G/GEJ adenocarcinoma. Compared with TLSs-negative patients, TLSs-positive patients had a better tendency for DCB (8 [66.7%] of 12 vs 6 [42.9%] of 14, p = 0.267) (supplementary Fig. 9). Additionally, TLSs were identified in 14 cases (46.6%), a rate considerably lower than the prevalence of TLSs observed in AFP-negative GC.^23,24^ These observations suggest that the TME of AFP-G/GEJ adenocarcinoma may be “colder” than that of AFP-negative gastric adenocarcinoma.

#### Association between molecular features and tumor-infiltrating cells

Considering the potential predictive significance of LRP1B and PI3K pathway status, we examined the association between LRP1B status and cell infiltration in the TME. The presence of LRP1B mutation was associated with a reduction in the infiltration of CD8^+^ T cells and PD-1^+^CD8^+^ T cells compared to tumors that did not contain LRP1B mutation (supplementary Table 5). Additionally, alterations in the PI3K pathway were associated with reduced infiltration of CD3^+^, CD4^+^, and CD8^+^ T cells (supplementary Table 6).

#### Association between spatial distribution characteristics and durable clinical benefit

**Fig. 5:**
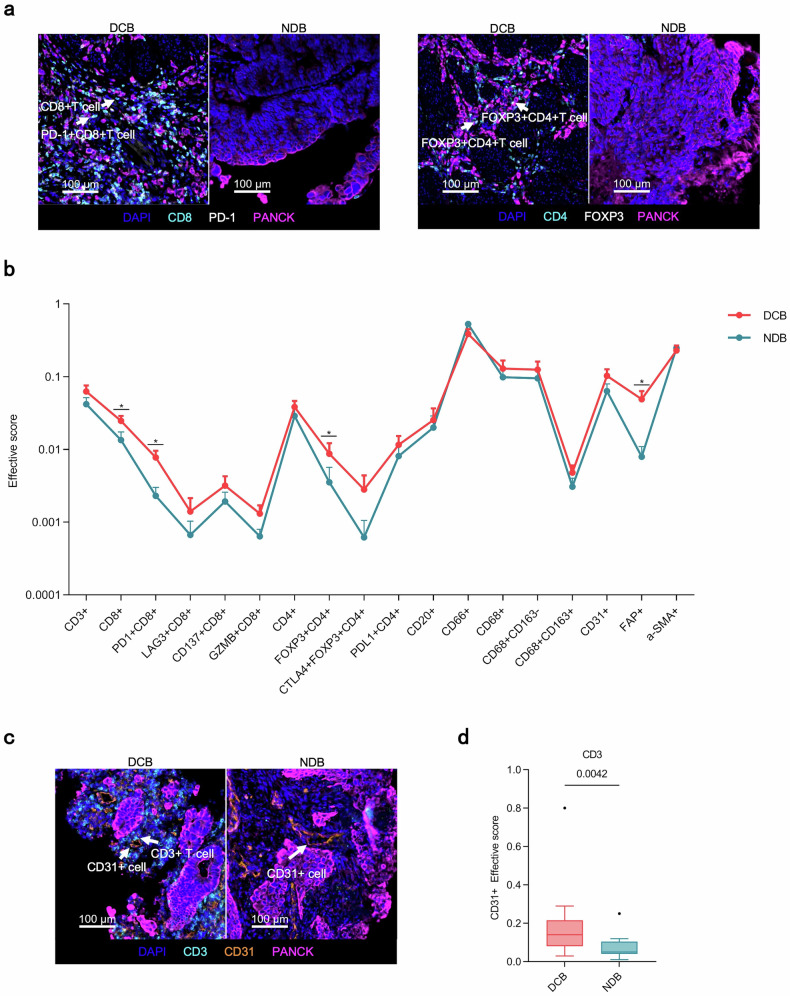
Spatial analysis of AFP-G/GEJ adenocarcinoma shows a hierarchy of organization of TICs in diffirent treating efficacy. **a** Representative multiplex immunofluorescence images of spatial analysis from DCB group and NDB group. Arrowheads in left rows denote CD8^+^ T and PD-1^+^CD8^+^ T cells while those in the right denote FOXP3^+^CD4^+^ T cells. **b** The distribution of the effective score of TICs populations in the tumor parenchyma in 10 µm increments between DCB (n = 14) and NDB (n = 12). Error bars represent mean ± SEM. **c** Representative multiplex immunofluorescence images of spatial analysis from DCB group and NDB group. Arrowheads denote CD3^+^ T cells and CD31^+^ cells. **d** The effective score of CD3^+^ T cell populations around CD31^+^ cells in the tumor parenchyma in 10 µm increments between DCB (n = 14) and NDB (n = 12). *P* values in b and d were calculated using the two-sided Mann-Whitney U-test. The line in the middle of the box represents the median. The lower and the upper edges of the box are the 1st and 3rd quartiles, respectively. The dots are considered outliers, which are more than 1.5*IQR beyond the lower or upper quartiles

We further analyzed the spatial distribution characteristics of TICs in AFP-G/GEJ adenocarcinoma. Lymphocytes including CD3^+^ T, CD4^+^ T, CD8^+^ T cells, and CD20^+^ B cells were mainly distributed in the stroma region, but myeloid cells such as CD66^+^ neutrophils, CD68^+^ macrophages were located more in the tumor parenchyma, highlighting the particular distribution of tumor-infiltrating immune cells in AFP-G/GEJ adenocarcinoma (supplementary Fig. 10). We then evaluated the prognostic value of the density of TICs by distribution region. The data indicated that CD8^+^ T cells and PD-1^+^CD8^+^ T cells exhibited a similar trend in both contexts. Specifically, the densities in the DCB were significantly higher than in the NDB (Supplementary Tables 7–8). With the precise localization of central cells and surrounding cells established, we then assessed the clinical relevance of the proximity between individual tumor cells (central cells) and TICs (surrounding cells) within the tumor parenchyma by the “effective score” parameter established previously.^25^ The radius (10μm) was preselected in order to identify surrounding cell populations that were likely capable of effective cell-to-cell interaction with central cells (supplementary Fig. 11). The effective scores of CD8^+^ T cells, PD-1^+^CD8^+^ T cells, FOXP3^+^CD4^+^ T cells and FAP^+^ cells, which represents cancer-associated fibroblasts (CAFs), were higher in the DCB than those in the NDB (Fig. 5a, b). Also, as our treatment regimen included the anti-angiogenic agent apatinib, we further investigated the effective scores of the cells surrounding the CD31^+^ cells within 10 μm radius to explore the interactions between TICs and blood vessels. Notably, the effective scores of CD3^+^ T cells and FAP^+^ cells were higher in the DCB group, whereas the effective scores of CD8^+^ T cells did not differ between the two groups (Fig. 5c, d; supplementary Fig. 12). Recent studies suggest that perivascular CAFs chemotactically recruit T cells into the tumor parenchyma.^26^ In this study, we observed a positive correlation between the effective score of CAFs around tumor blood vessels and the effective score of T cells. In contrast, no such correlation was observed for CAFs surrounding tumor cells (supplementary Fig. 13a, b).

#### Increased density of T cells upon treatment

Finally, we assessed the temporal dynamics of TME corresponding to the ICIs-combined treatment. Post-treatment, a significant increase in both CD3^+^ T cells and CD8^+^ T cells was observed, suggesting that camrelizumab plus apatinib and SOX could recruit T cells and remodel a more ‘inflamed’ TME in AFP-G/GEJ adenocarcinoma (supplementary Fig. 14).

## Discussion

As far as we know, this represents the first clinical trial specifically targeting AFP-G/GEJ cancer, a subtype of G/GEJ cancer, challenged by a more immunosuppressive TME.^8^ This trial achieved its primary endpoint with an ORR of 66.7%, markedly higher than the ORR of 30% for chemotherapy alone (historical control) in AFP-G/GEJ cancer patients observed in our previous study.^27^ Our regimen was tolerated, and the safety profile aligns with the SPACE study, with no new safety signals identified.^15^ Common TRAEs, mainly hematologic toxicities, were managed through dose modifications and supportive care, allowing most patients to continue treatment. No patients stopped the combination therapy because of TRAEs, and only 3 (8.3%) patients stopped any investigational drug because of TRAEs (one each for chemotherapy, camrelizumab, and apatinib). The survival outcomes of AFP-G/GEJ cancer were more favorable than previously reported, with 6 patients in our study even achieving long-term survival (PFS > 14 months).

In the design of this study, chemoimmunotherapy has not been established as the standard therapy for untreated G/GEJ cancer.^28^ Therefore, this trial did not include such a combination as a control arm. Currently, chemoimmunotherapy has become the recommended therapy for untreated G/GEJ cancer. Previously reported ORR and PFS for chemoimmunotherapy in general G/GEJ cancer ranged from 51% to 58% and 6.9 to 7.7 months, respectively.^29–31^ However, only two small-scale retrospective studies reported efficacy of chemoimmunotherapy for AFP-G/GEJ cancer, likely due to its rarity.^4,32^ A retrospective study compared 7 patients with AFP-GC receiving chemoimmunotherapy and 21 patients receiving chemotherapy alone. The study reported a median PFS of 22.0 months versus 4.3 months and a median OS that was not reached versus 14.0 months, respectively.^32^ Another retrospective study showed that higher levels of baseline AFP correlated with reduced efficacy of immunotherapy, with a DCR of 50.0% in patients with high AFP levels and 87.7% in those with low AFP levels.^4^ Comparing their efficacy data with ours is challenging due to the small patient number and the obvious differences in patient disease characteristics and the inclusion of those previously treated.^4,32^

In recent phase 3 trials, 6 cycles of chemotherapy plus immunotherapy showed survival benefits and tolerability in untreated advanced GC.^29,30,33^ In this study, we used a shorter chemo-combination duration of 4 cycles and observed that PD occurred mainly between 3 and 6 months during maintenance treatment, with most patients achieving a confirmed PR as their best response. These findings underscore the need to explore whether extending the chemo-combination to 6 or 8 cycles could further improve its efficacy in treating AFP-G/GEJ cancer.

The proportions of AFP-G/GEJ cancer cases exhibiting PD-L1 positivity in our trial were notably lower than in CheckMate 649 study (CPS ≥ 1 at 81%; CPS ≥ 5 at 60%), KEYNOTE-859 study (CPS ≥ 1 at 78%; CPS ≥ 10 at 35%) and ORIENT-16 study (CPS ≥ 1 at 84.1%; CPS ≥ 5 at 60.2%).^29–31^ These results somewhat support the previous observations that immunosuppression in AFP-GC is more pronounced than in non-AFP-GC, warranting cautious interpretation due to the small sample size and potential bias.^8^ Notably, our subgroup analysis indicated a trend toward improved ORR in patients with CPS ≥ 5, consistent with PD-L1’s value in predicting immunotherapy efficacy in general GC.^34^

Biomarker analyses indicated that LRP1B mutations and PI3K pathway changes correlated with reduced durable clinical efficacy and lower T cells densities. LRP1B, a common tumor suppressor in solid tumors, could influence infiltration of immune cells, and its mutation correlated with poor prognosis in gastric cancer.^35–38^ The PI3K pathway, involved in key cancer hallmarks, regulated the tumor immune microenvironment.^39,40^ In dMMR/MSI-H gastric cancer, PI3K pathway mutations correlated with lower immune cell infiltration and poorer outcomes.^41^ These findings suggested that modifications in the intrinsic characteristics of tumor cells may potentially modulate the response to treatment by influencing the TME.

Our study offered the first comprehensive characterization of the TME in AFP-G/GEJ adenocarcinoma, using TICs density and spatial distribution as key insights. Pretreatment density or effective score of PD-1^+^CD8^+^ T cells predicted response to immune-combination therapy.^42,43^ This study further underscored the predictive value of PD-1^+^CD8^+^ T cells as biomarkers for immunotherapy durable efficacy in AFP-G/GEJ adenocarcinoma. While Tregs had traditionally been considered immunosuppressive, studies like CheckMate649 and SPACE showed that high FOXP3^+^ Treg levels correlated with improved survival.^15,44^ Furthermore, a recent study found that different Tregs subsets were associated with distinct survival outcomes.^45^ Our study found the density and effective score of (CTLA4^+^)FOXP3^+^CD4^+^ T cells were linked to durable responses, further emphasizing the need for additional analysis of Treg subsets. CAFs were key pro-tumor components in the TME and were contributing to poor prognosis.^46–48^ However, some studies suggested that CAFs may acquire an anti-tumor phenotype and counteract tumor progression.^49,50^ Recent research emphasized that CAFs signaled immune cells in tumor niches, with perivascular CAFs recruiting T cells into the tumor.^26^ Our finding supported the aforementioned conclusions, suggesting that AFP-G/GEJ cancer may harbor a CAF subpopulation around blood vessels that recruited T cells into the tumor parenchyma. Our study found that tumors in patients with durable clinical benefit exhibited increased accumulation of T cells within perivascular regions. Aberrant tumor vasculature often leads to hypoxia and immunosuppression, hindering the efficacy of immunotherapy.^51^ Tumor vascular normalization can mitigate these challenges.^51^ Stimulated CD4^+^ T lymphocytes are capable of mediating vessel normalization by localizing to the vicinity of tumor endothelial cells and altering the cytokine milieu.^52^ Furthermore, accumulation of CD8^+^ T cells and increased interferon-γ production enhance vessel perfusion and bolster anti-tumor immunity.^53^ Building on these findings, we hypothesize that combination therapy may be able to activate T cells enriched around tumor-associated blood vessels (CD31^+^ endothelial cells), promote tumor vasculature normalization, and induce durable clinical benefit. The detailed mechanisms of the T cell-tumor vasculature interactions induced by anti-VEGFR agent plus ICI treatments in AFPGC warrant further investigation.

The current trial is confined by its non-randomized design and a small cohort size, which inherently limits the generalizability of the findings, especially the conclusions drawn from the subgroup analysis. The exploratory nature of this study necessitates cautious interpretation of the data. The lack of a control arm for immunotherapy plus chemotherapy precludes factorial analysis, which is crucial to isolate the antitumor response attributable to the addition of apatinib. The results of our biomarker analysis are preliminary and further validation is required.

The combination of camrelizumab, apatinib, and SOX represents a viable treatment choice for untreated AFP-G/GEJ adenocarcinoma. Our exploratory analysis suggests that those with greater pre-treatment number of (PD-1^+^ ) CD8^+^ T cells and effective scores have a greater likelihood of deriving benefit from the treatment in the long term. Future randomized controlled trials should evaluate the benefits of adding apatinib to chemoimmunotherapy compared with the current standard chemoimmunotherapy in AFP-G/GEJ adenocarcinoma.

## Materials and methods

This multi-center, single-arm trial was conducted at 3 study sites in China and was registered on ClinicalTrials.gov (Identifier: NCT04609176). It was executed in alignment with the Declaration of Helsinki and Good Clinical Practice guidelines, with approval from the ethics committees of Peking University Cancer Hospital & Institute (2020YJZ54). Each participant provided informed consent.

### Patients

Eligible patients were aged ≥18 years with histological diagnosis of G/GEJ adenocarcinoma; had locally advanced or metastatic disease with stage III-IV (AJCC 8th edition TNM stage); had unresectable disease assessed by multidisciplinary teams; had not received previous systemic treatments for metastatic disease, except for neoadjuvant therapies or recurrence occurred more than 6 months after adjuvant therapies; had Eastern Cooperative Oncology Group performance status of 0-2; had measurable lesions per RECIST v1.1; had serum AFP levels > 2× upper limit of normal or AFP-positive by IHC staining; had adequate organ function. Exclusion criteria were HER2-positive status (IHC3+ or IHC2 + /ISH + ); had prior anti-PD-1/PD-L1 therapies or apatinib exposure.

### Procedures

Eligible patients were administered 4 cycles of SOX combined with camrelizumab (200 mg, iv, d1, q3w) and apatinib (250 mg, po, qd). Patients who did not experience disease progression continued treatment with camrelizumab and apatinib for a maximum of 24 months, or upon progression, intolerance, consent withdrawal, loss to follow-up, or death. Each treatment cycle was 21 days. Treatment details and dose adjustments are provided in the supplement.

Tumor assessments were evaluated per RECIST v1.1 and conducted every 6 to 8 weeks, with at least a four-week interval for confirmation, until radiological progression, initiation of new anticancer treatment, consent withdrawal, loss of visits, or deaths. We continuously monitored AEs and assessed AEs per the National Cancer Institute Common Terminology Criteria Adverse Events Version 5.0.

### Outcomes

The primary endpoint was confirmed ORR as per RECIST v1.1. Secondary endpoints were DCR, DoR, TTR, PFS, OS, and safety. ORR was defined as the proportion of patients achieving CR or PR as best overall response per RECIST v1.1. Definitions of secondary endpoints are detailed in the supplement. Exploratory endpoints included biomarker analysis to investigate the correlation between biomarker and treatment response or clinical benefit.

### Exploratory analysis

Tumor specimens were acquired from 26 pre-treatment tumor biopsies and 6 paired post-treatment tumor biopsies for mIF staining (see supplement). Tumor parenchyma and stroma region were differentiated by CK staining and a pathologist. The density of diverse cell subsets was quantified as the count of positively stained cells per mm^2^.

Targeted exome NGS is detailed in the supplement. Panel TMB was determined by measuring the number of nonsynonymous somatic mutations within the targeted gene sequencing region.

### Statistical analysis

In this single-arm trial, Simon’s two-stage optimal approach (Simon, 1989) was used to estimate sample size. A retrospective review of 105 AFP-G/GEJ cancer patients (AFP ≥ 160 ng/ml) who received first-line chemotherapy at our center showed an ORR of 30%.^27^ We assumed that our new treatment regimen could increase the ORR to 55% in AFP-G/GEJ cancer patients. Assuming a one-sided type-I-error of 5% and statistical power of 80%, the first stage required 9 patients and if at least 4 patients achieved confirmed CR or PR as per RECIST v1.1, the study would proceed to the second stage and continue to enroll another 26 evaluable patients. In total, 35 evaluable patients were required. To establish a significant result, it was required to have at least 15 patients achieving confirmed CR or PR out of 35 evaluable patients at the end of the second stage. Patients who received study treatment were included in the efficacy and safety assessments.

Continuous variables were summarized using the median (range), and categorical variables were presented as counts and percentages (n [%]). ORR and DCR were presented along with their corresponding 95% Clopper-Pearson confidence intervals. PFS, DoR, and OS were analyzed using Kaplan-Meier method, the 95% CIs for the survival probability at specific timepoints and for the median time were calculated based on Greenwood method and Brookmeyer-Crowley method respectively. All statistical analyses were performed using SAS software (v.9.4), RStudio (v.4.2.1) and GraphPad Prism (v.9.4.1). GraphPad Prism (v.9.4.1) and the R packages including ggplot2 (v.3.4.4), ComplexHeatmap (v.2.18.0) and pheatmap (v.1.0.12) were used for plotting. Figure 3a and supplementary Fig. 12 were created with the permission of BioRender.com.

## Supplementary information

Supplement-clean version

Study protocol

## Data Availability

The corresponding author can provide the clinical data from this study upon reasonable request. The raw sequencing data generated in this study have been deposited in the Genome Sequence Archive (GSA) under the accession code HRA010458. In accordance with data privacy laws and patient consent requirements for data sharing, access to these data is restricted and managed through controlled mechanisms. Researchers who request access to raw data are required to submit a formal request to the Data Access Committee of the GSA-human database.^54,55^
