# Supplementary material for: PD-1 antibody camrelizumab plus apatinib and SOX as first-line treatment in patients with AFP-producing gastric or gastro-esophageal junction adenocarcinoma (CAP 06): a multi-center, single-arm, phase 2 trial
Source: Signal Transduct Target Ther. 2025 Mar 14;10:100. doi: 10.1038/s41392-025-02193-z (PMC11906745; doi:10.1038/s41392-025-02193-z)
Supplement: Supplementary file 1 — Supplement-clean version [file 41392_2025_2193_MOESM1_ESM.docx]

Supplementary Materials for

PD-1 antibody camrelizumab plus apatinib and SOX as first-line treatment in patients with AFP-producing gastric or gastro-esophageal junction adenocarcinoma (CAP 06): a multi-center, single-arm, phase 2 trial

Yakun Wang^1*^, Jialin Lu^2*^, Xiaoyi Chong^2*^, Chang Wang^3*^, Xiaofeng Chen^4,5,6*^, Zhi Peng^1^, Yanhong Gu^4,5^, Yizhuo Wang^3^, XichengWang^2^, Jian Li^1^, Jifang Gong^1^, Changsong Qi^7^, Jiajia Yuan^2^, Zhihao Lu^2^, Ming Lu^1^, Jun Zhou^2^, Yanshuo Cao^2^, Yang Chen^2^, Cheng Zhang^1^, Zhiguo Hou^8^, Hongyi Kou^8^, Lin Shen^1#^, Xiaotian Zhang^1,9#^

Correspondence to:

Prof. Lin Shen, Email: shenlin@bjmu.edu.cn

Prof. Xiaotian Zhang, Email: zhangxiaotian@bjmu.edu.cn

**This PDF file includes:**

Supplementary methods

Figures. S1 to S14

Tables S1 to S8

**Supplementary Methods**

**Treatment details and dose adjustments**

The initial dose of S-1 was tailored by body surface area (BSA): 40 mg bid for BSA <1.25m^2^, 50 mg bid for BSA 1.25-1.5 m^2^, and 60 mg bid for BSA >1.5 m^2^, given orally on days 1 to 14 at each cycle. Oxaliplatin was given intravenously at 130 mg/m^2^ for 2-4 hours on day 1 at each cycle. Camrelizumab was administered prior to oxaliplatin, with a minimum 30-minute interval. Dose modifications (reduction, delay, or discontinuation) for S-1, oxaliplatin, and apatinib were permitted to manage adverse events (AEs). For camrelizumab, dose delay or discontinuation, but not dose reduction, were allowed. Any AEs related to S-1 or oxaliplatin unresolved within 42 days necessitated the discontinuation of the implicated agent.

**Definition** **of secondary endpoints**

Disease control rate (DCR) was defined as the proportion of patients achieving CR, PR or SD. DoR was defined as the time from the date of the first documented CR/PR to the date of documented disease progression or death due to any cause, whichever occurred first. Time to response (TTR) was defined as the time from treatment initiation to the first documented CR/PR. Progression-free survival (PFS) was defined as the time from treatment initiation to the documented disease progression or death due to any cause, whichever occurred first. Overall survival (OS) was defined as the time from treatment initiation to the date of death from any cause.

After treatment completion, patients were followed every three months to record data on survival status and subsequent anti-cancer treatments until death or loss to follow-up.

**Multiplex immunofluorescence (mIF) staining**

Tumor specimens were acquired from 26 pre-treatment tumor biopsies and 6 paired post-treatment tumor biopsies for mIF staining. All tissues were formalin-fixed, paraffin-embedded, and sectioned as slides of 4-μm thickness. The slides were deparaffinized 30 mins in xylene further rehydrated 5 mins in absolute ethyl alcohol, 5 mins in 95% ethyl alcohol and 2 mins in 75% ethyl alcohol immediately. Then, slides were washed three times and submersed in boiling EDTA buffer (ZSGB Biotech) 15 mins for epitope retrieval. Antibody diluent/blocking (Alpha X Biotech) was used for blocking. The mIF experiments were performed and analyzed with antibodies against CD3 (ZM0417, ZSGB Biotech), CD8 (ZA0508, ZSGB Biotech), PD-1 (ZM0381, ZSGB Biotech), CD137 (M1701-11, Huabio), LAG3 (ab209236, Abcam), GZMB (ab134933, Abcam), CD4(ET1609-52, Huabio), FOXP3 (ab20034, Abcam), CTLA4 (ZM0035, ZSGB Biotech), PD-L1 (13684S, CST), CD20 (ab78237, Abcam), CD66 (ab197678, Abcam), CD68 (ZM0060, ZSGB Biotech), CD163 (ZM0428, ZSGB Biotech), CD31 (66065-2-Ig, Proteinech), VEGFR2 (26415-1-AP, Thermofisher), FAP (ab207178, Abcam), α-SMA (ab124964, Abcam) and PANCK (ZM0069, ZSGB Biotech). All primary antibodies were cultured at 37 °C (60 mins) then slides were cultured at 37 °C (10 mins). The AlphaTSA Multiplex IHC Kits (AXT37100041, Alpha X Bio) were then used for visualization. After each staining cycle, both primary and secondary antibodies were removed through heat-induced epitope retrieval. Nuclei of slides were finally counterstained (5 mins) through DAPI and enfolded in a mounting medium. Multispectral images were scanned with Axioscan 7 Microscopy (Zeiss), while cells of interest were quantified through HALO image analysis (Indica Labs). Tumor parenchyma and stroma region were differentiated by CK staining and a pathologist. The density of various cell subsets was quantified as the number of positively stained cells per mm^2^. Immune cell subsets in tumor parenchyma and stromal region were identified by detecting signals for markers including CD3^+^, CD8^+^, PD-1^+^CD8^+^, LAG3^+^CD8^+^, CD137^+^CD8^+^, GZMB^+^CD8^+^, CD4^+^, FOXP3^+^CD4^+^, CTLA4^+^FOXP3^+^CD4^+^, PDL1^+^CD4^+^, CD20^+^, CD66^+^, CD68^+^, CD68^+^CD163^-^, CD68^+^CD163^+^, CD31^+^, VEGFR2^+^, FAP^+^, α-SMA^+^. Co-occurrence of CD3^+^ T cells and CD20^+^ B cells indicates tertiary lymphoid structure (TLS) formation.

**Tumor Genomic Profiling**

Genomic DNA from FFPE tissues was extracted using the Maxwell RSC FFPE plus DNA kit (Promega, Cat no. AS1720). For DNA extraction from peripheral blood lymphocytes, the Blood gDNA purification kit (Concert, Cat: RC1001) was utilized. Subsequently, 100ng of genomic DNA was subjected to shearing, targeting fragment sizes of 200 bp, using the Covaris E210 system (Covaris, Inc.). We performed next-generation sequencing of tumor and gDNA-matched germline DNA for library preparation using KAPA HyperPrep Kit (Roche,07962312001) and Agilent SureSelect XT kit (Agilent, G9702C). Following library preparation, library quantification was conducted using the Qubit 3.0 Fluorometer (Life Technologies, Inc.), and assessment of quality and fragment size was performed using the Agilent 2100 Bioanalyzer (Agilent Technologies, Inc.). Subsequently, the samples underwent paired-end sequencing on an Illumina Nova Seq 6000 platform (Illumina Inc., USA), with reads spanning 150 base pairs. The raw Illumina sequence data underwent demultiplexing and subsequent conversion into fastq files. Post-adaptor removal and trimming of low-quality sequences, the obtained qualified reads were employed for somatic variants and mutational signature analysis as outlined below.

The raw sequencing data underwent alignment against the reference human genome (UCSC hg19) using the Burrows-Wheeler Aligner (BWA). Subsequently, duplicate reads were eliminated, and local realignment procedures were executed. The Genome Analysis Toolkit (GATK) was utilized for calling single nucleotide variations (SNVs), insertions, and deletions (indels). Following this, germline alterations were removed by comparing the matched blood samples, resulting in the identification of somatic alterations. To annotate the variants, the ANNOVAR software tool was employed.

Somatic variants displaying allele frequencies (AF) exceeding 0.5% were derived from each tumor genomic DNA samples by excluding germline variants. These identified somatic variants were subsequently annotated using Ensembl Variant Effect Predictor (VEP)^1^. The functional categorization of each somatic mutation adhered to the interpretation and reporting standards and guidelines set forth by the Association for Molecular Pathology, American Society of Clinical Oncology, and College of American Pathologists (ASCO/CAP). Copy number analysis was conducted using CNVkit. Altered genes were also classified according to Kyoto Encyclopedia of Genes and Genomes (KEGG) pathways. Panel TMB was assessed by quantifying the number of nonsynonymous somatic mutations within the targeted gene sequencing region.

**Reference**

1 McLaren, W. *et al.* The Ensembl Variant Effect Predictor. *Genome Biol* **17**, 122 (2016).

**Supplementary Results**

**Objective response rate**

Among all 36 patients, the 90% binomial confidence interval (CI) of confirmed objective response rate in our trial was 51.7% to 79.5%.

**Overall survival for patients who did not undergo surgery**

In this study, two patients (Patient 1004 and 1047; both had stage IVa before treatment) underwent surgery after achieving partial response and MDT discussion. The median overall survival (OS) for patients who did not undergo surgery (n=34) was 16.9 months (95%CI: 10.5-27.0). The 9-month and 12-month OS rates were 75.7% (95%CI: 57.2-87.1) and 65.7% (46.5-79.4), respectively.

**Intervention group 2**

In the intervention group 2, AFP-GC patients who had failed first-line chemotherapy were administered with camrelizumab (200 mg, iv, d1, q3w) plus apatinib (250 mg, po, qd). Between December 2020 and April 2022, 6 patients were enrolled. Considering the slow recruitment rate, enrollment for intervention group 2 was terminated early. As only 6 patients were enrolled, the Simon two-stage design requirement of 10 patients in the first stage was not met, and formal analysis was not conducted. We retrospectively reviewed the data of these 6 patients, the best of response was 3 stable disease and 3 disease progression. As of the cutoff date, these 6 patients discontinued study treatment due to disease progression; 5 patients had died, and one patient remained alive and under follow-up.


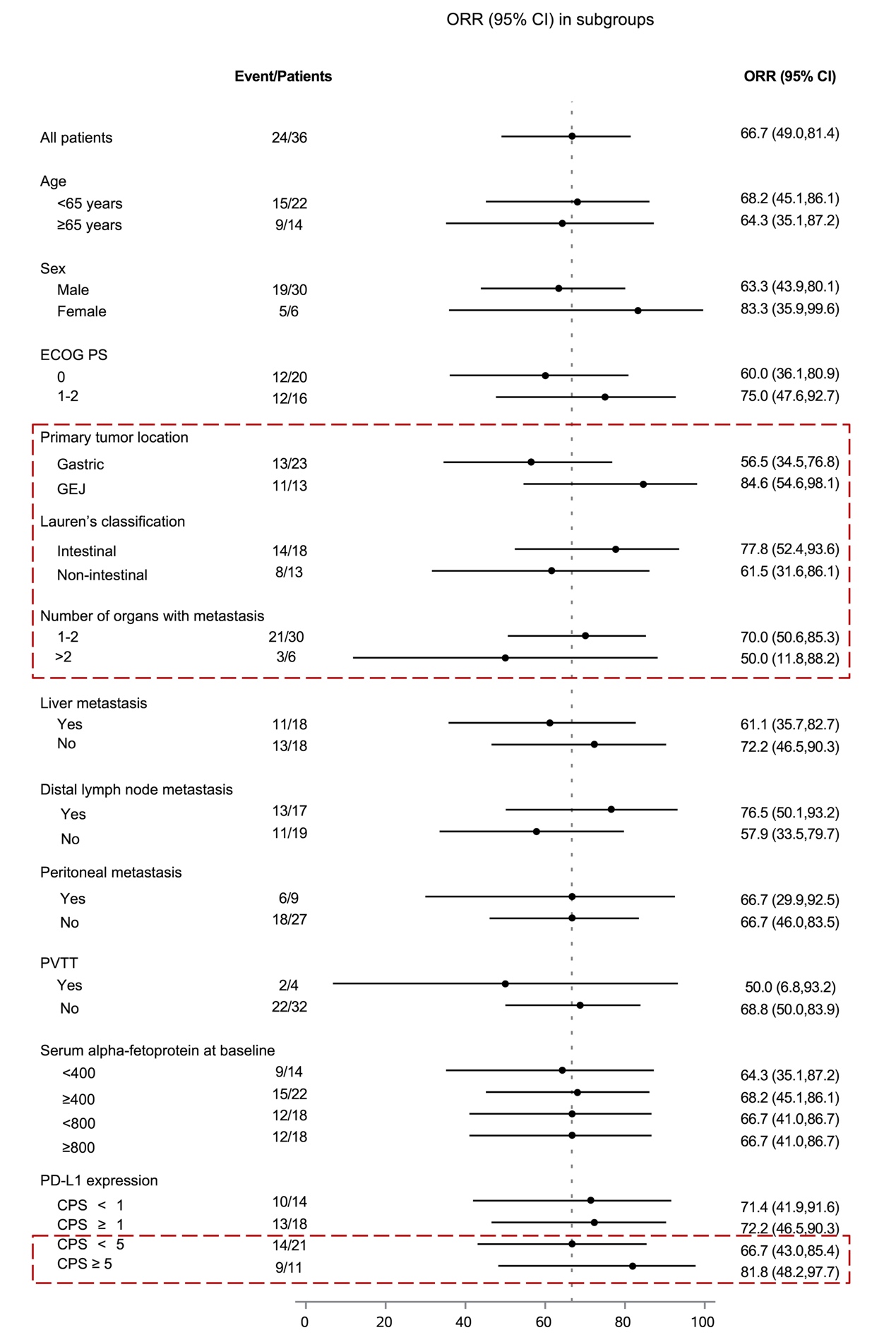


**Figure S1. Subgroup analysis of objective response rate based on baseline characteristics**

**
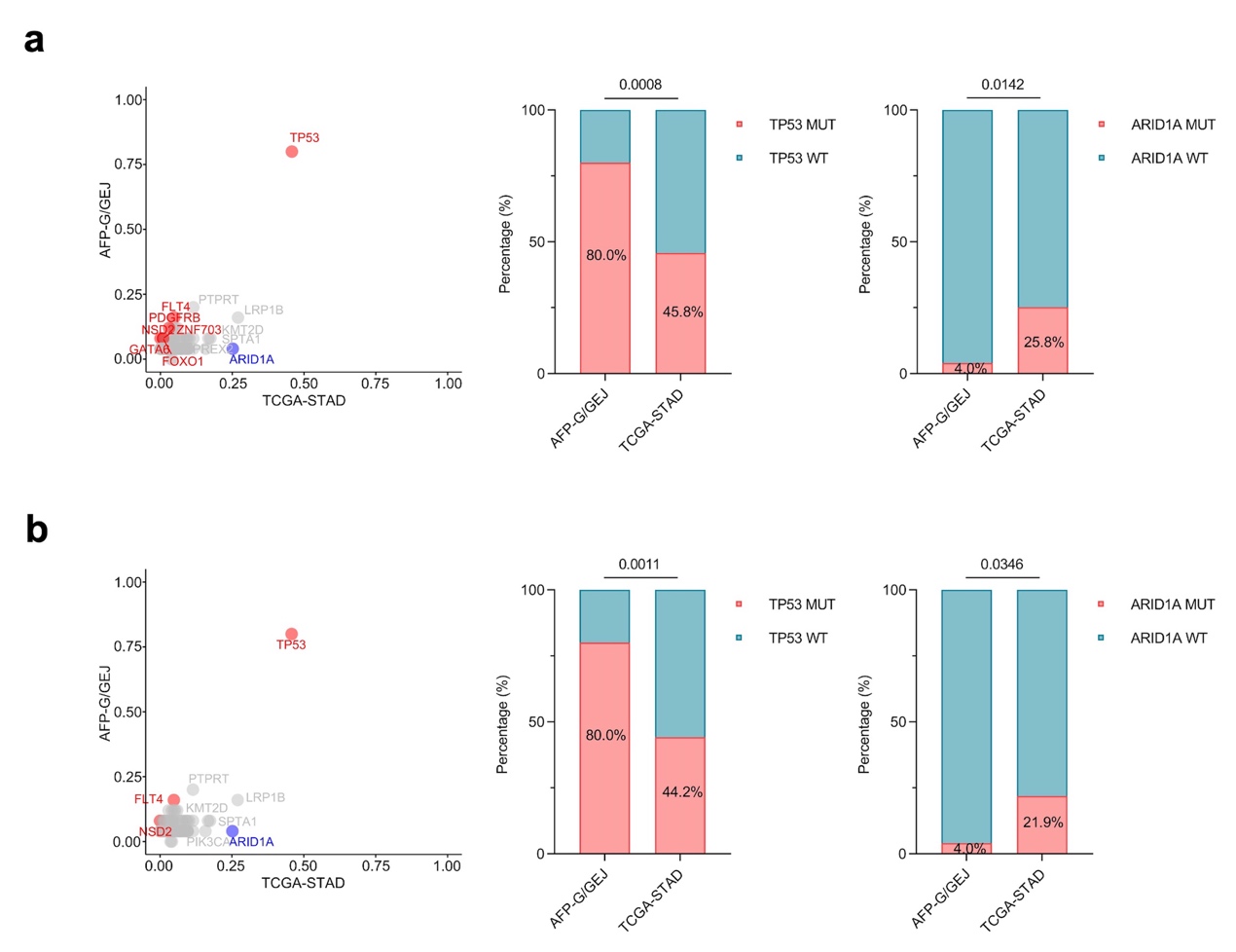
**

**Figure S2. Comparison of the molecular characteristics of AFP-G/GEJ adenocarcinoma and TCGA-STAD. a-b,** Gene mutation rates of AFP-G/GEJ adenocarcinoma in comparison with TCGA-STAD **(a)** or stage III and IV of TCGA-STAD **(b)**. Red dots, genes with significantly higher mutation rate in AFP-G/GEJ adenocarcinoma; blue dots, genes with significantly lower mutation rate in AFP-G/GEJ adenocarcinoma. P values in a and b were calculated using the Fisher’s exact test. WT, wildtype; MUT, mutation; TP53, Tumor Protein P53; ARID1A, AT-Rich Interaction Domain 1A.

**
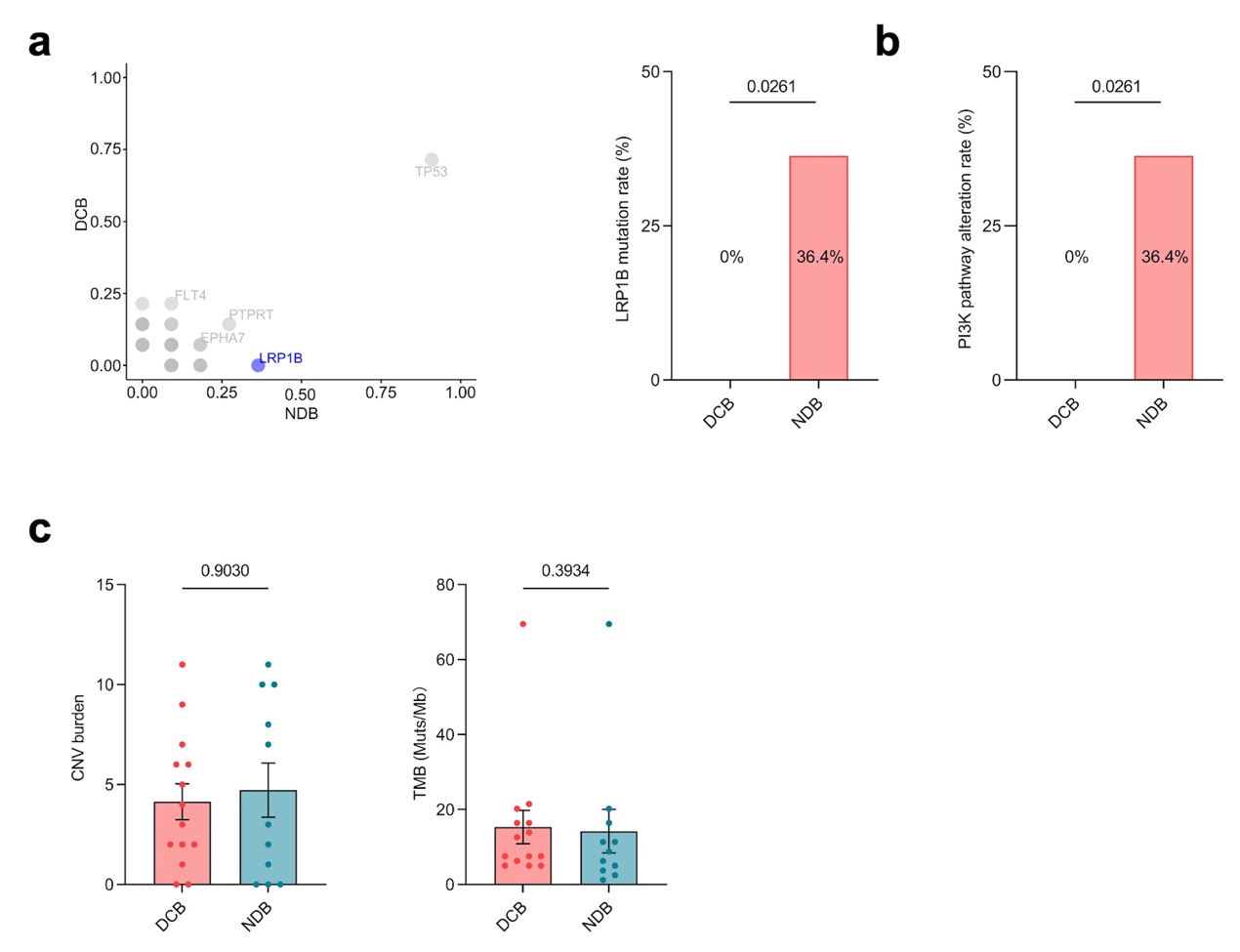
**

**Figure S3. Comparison of the molecular characteristics of DCB and NDB. a,** Comparison of the gene mutation rates of DCB (n = 14) and NDB (n = 11). Blue dots, genes with significantly lower mutation rate in DCB. **b,** Comparison of PI3K pathway alteration rates of DCB (n = 14) and NDB (n = 11). **c,** Differences in TMB and copy number variants burden between DCB (n = 14) and NDB (n = 11). P values in a and b were calculated using the Fisher’s exact test. P values in c were calculated using the two-sided Mann-Whitney U test. The line in the middle of the box represents the median. The lower and the upper edges of the box are the 1st and 3rd quartiles, respectively. The dots are considered outliers, which are more than 1.5*IQR beyond the lower or upper quartiles. LRP1B, Low Density Lipoprotein Receptor-Related Protein 1B; PI3K, Phosphoinositide 3-kinases; TMB, tumor mutation burden; CNV, copy number variants.

**
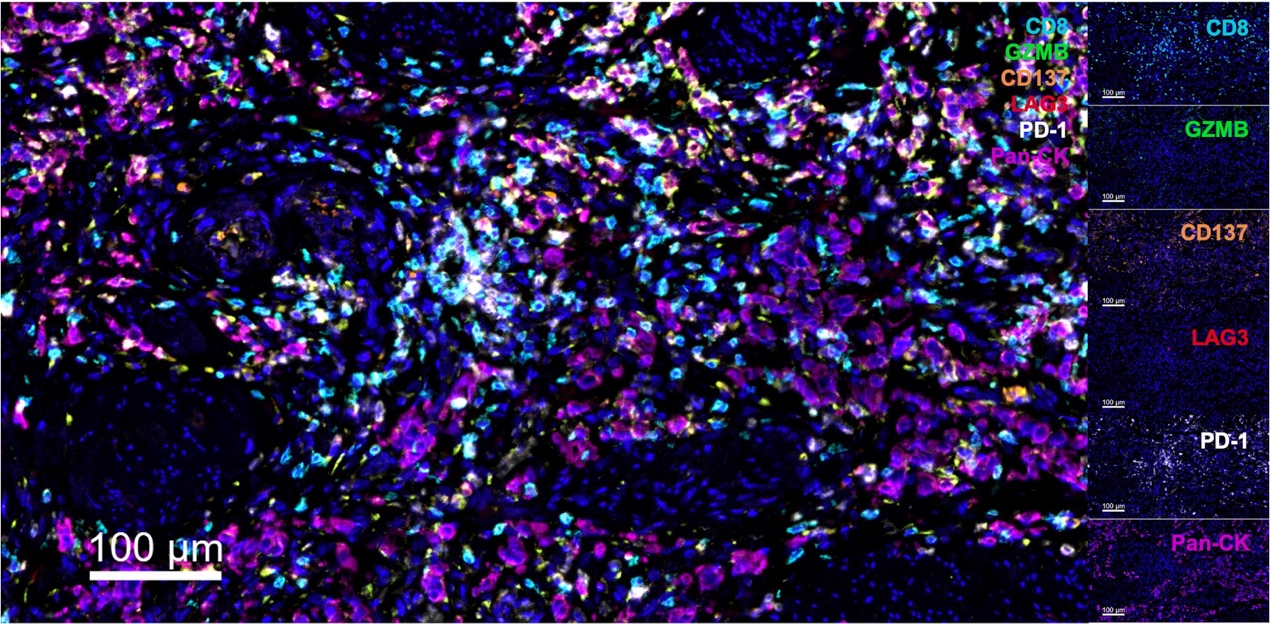
**

**Figure S4.** **Multiplex immunofluorescence staining for CD8, GZMB, CD137, LAG3, PD-1 and Pan-CK (Panel 1)**

**
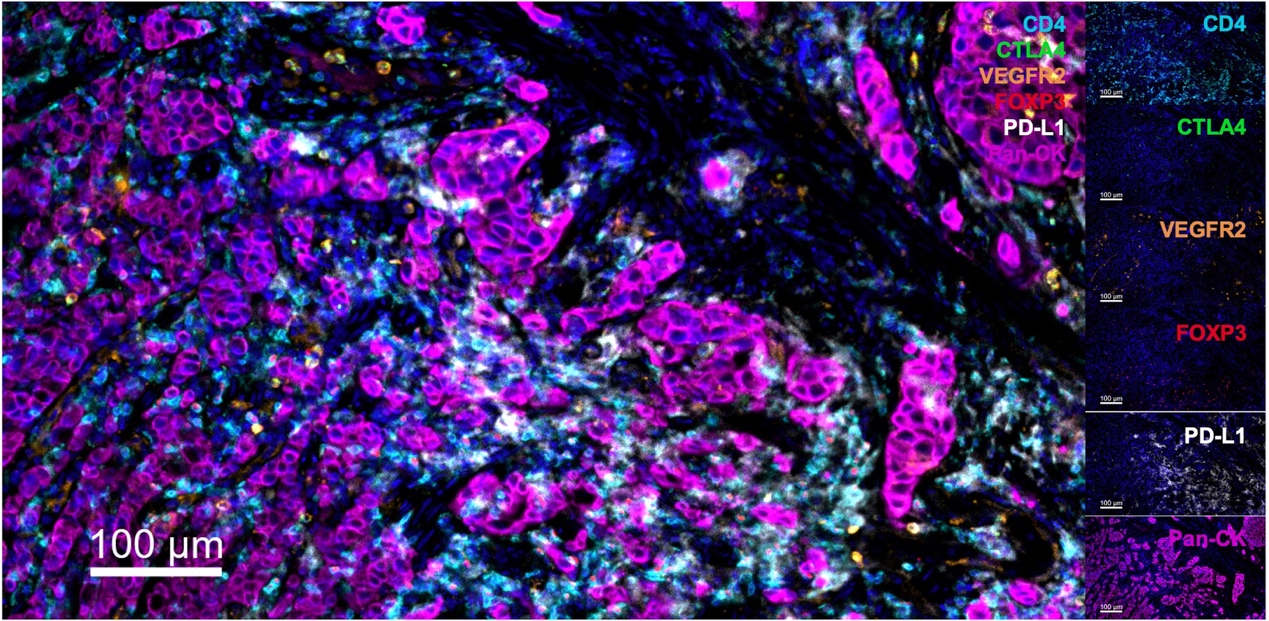
**

**Figure S5. Multiplex immunofluorescence staining for CD4, CTLA4, VEGFR2, FOXP3, PD-L1, and Pan-CK (Panel 2)**

**
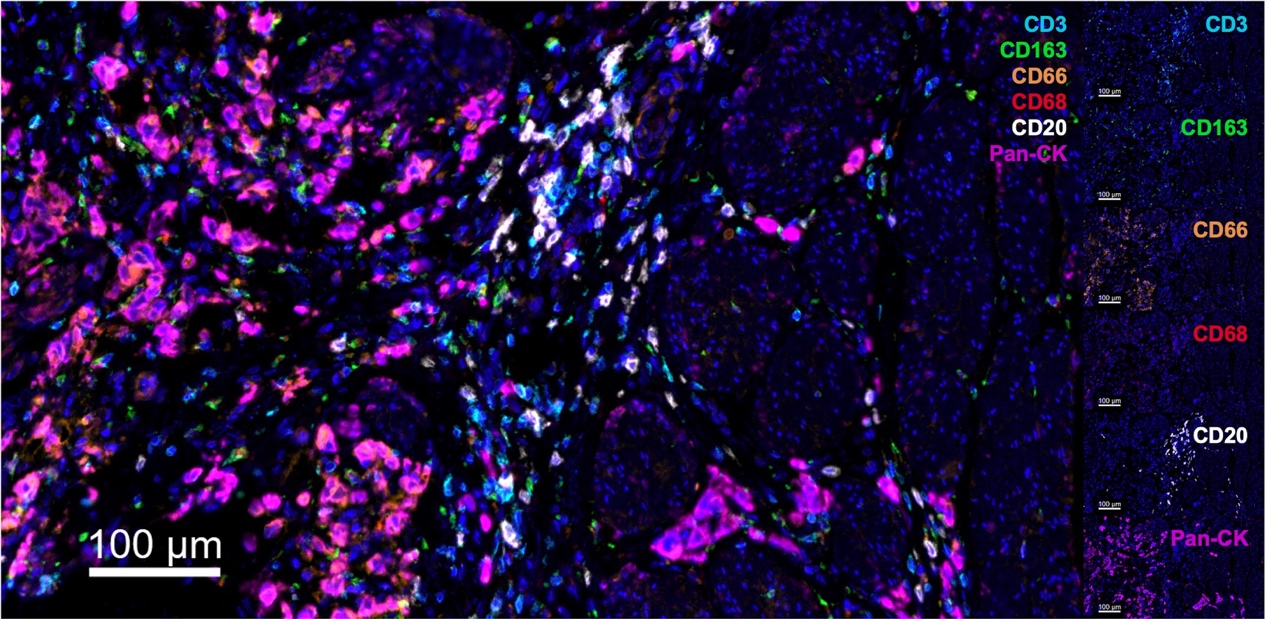
**

**Figure S6. Multiplex immunofluorescence staining for CD3, CD163, CD66, CD68, CD20 and Pan-CK (Panel 3)**

**
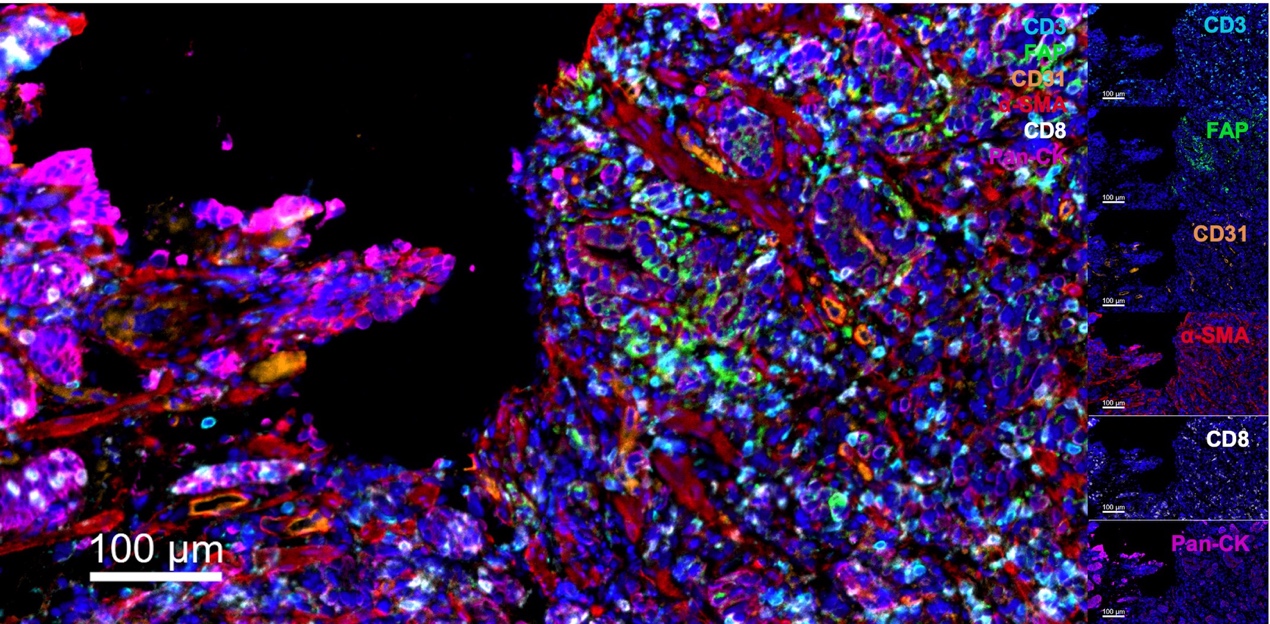
**

**Figure S7. Multiplex immunofluorescence staining for CD3, CD8, CD31, FAP, α-SMA and Pan- CK (Panel 4)**


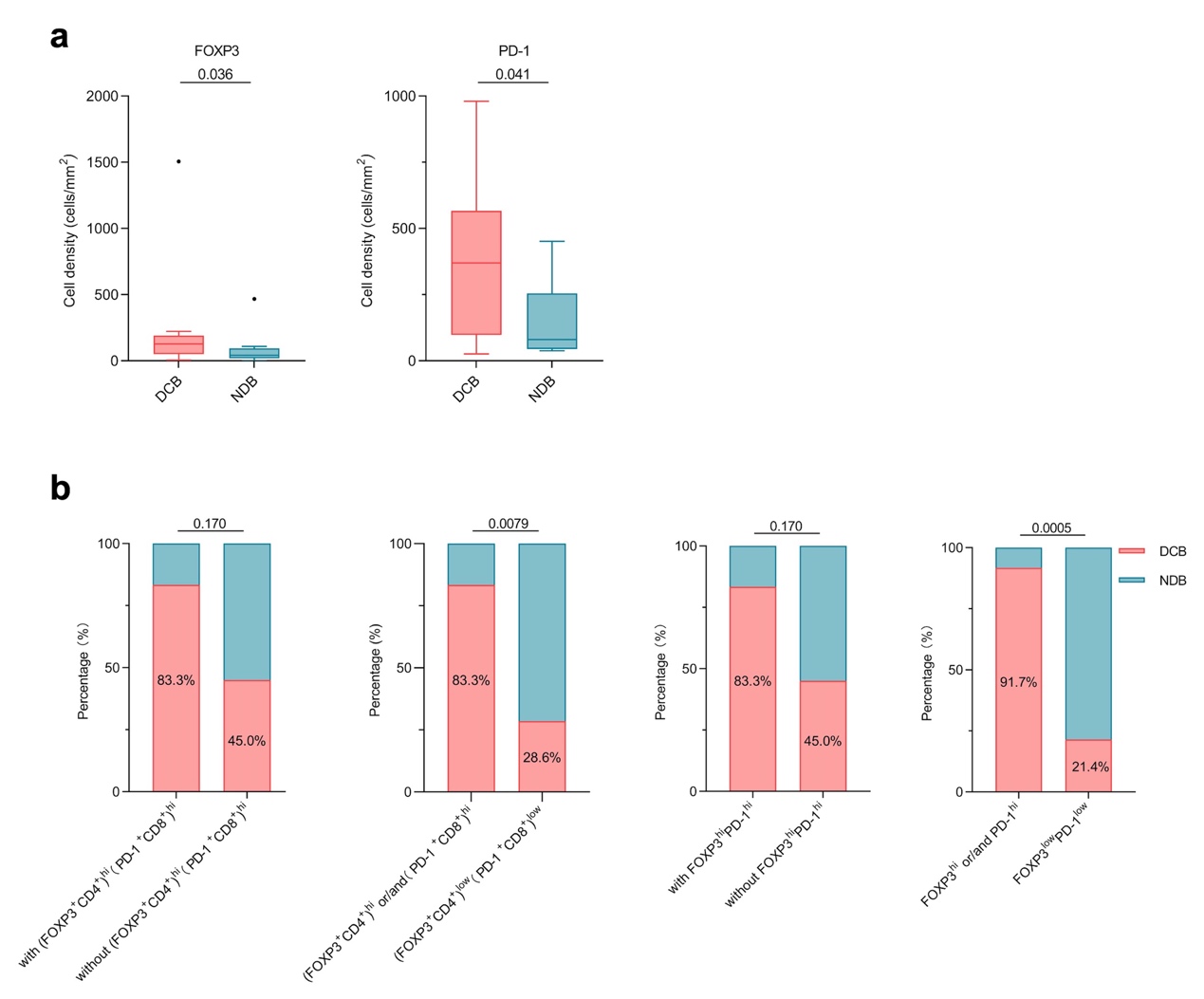


**Figure S8. Correlation of the expression of FOXP3^+^(CD4^+^) and PD-1^+^(CD8^+^) cells with therapeutic efficacy in AFP-G/GEJ adenocarcinoma. a,** Comparison of PD-1^+^ and FOXP3^+^ cells density between DCB (n=14) and NDB (n=12). **b,** Association between durable clinical benefit and the expression of FOXP3^+^(CD4^+^) and PD-1^+^(CD8^+^) cells from pre-treatment (DCB, n=14; NDB, n=12). P values in a were calculated using the two-sided Mann–Whitney U-test. The line in the middle of the box represents the median. The lower and the upper edges of the box are the 1st and 3rd quartiles, respectively. The dots are considered outliers, which are more than 1.5*IQR beyond the lower or upper quartiles. P values in b were calculated using the Fisher’s exact test.

**
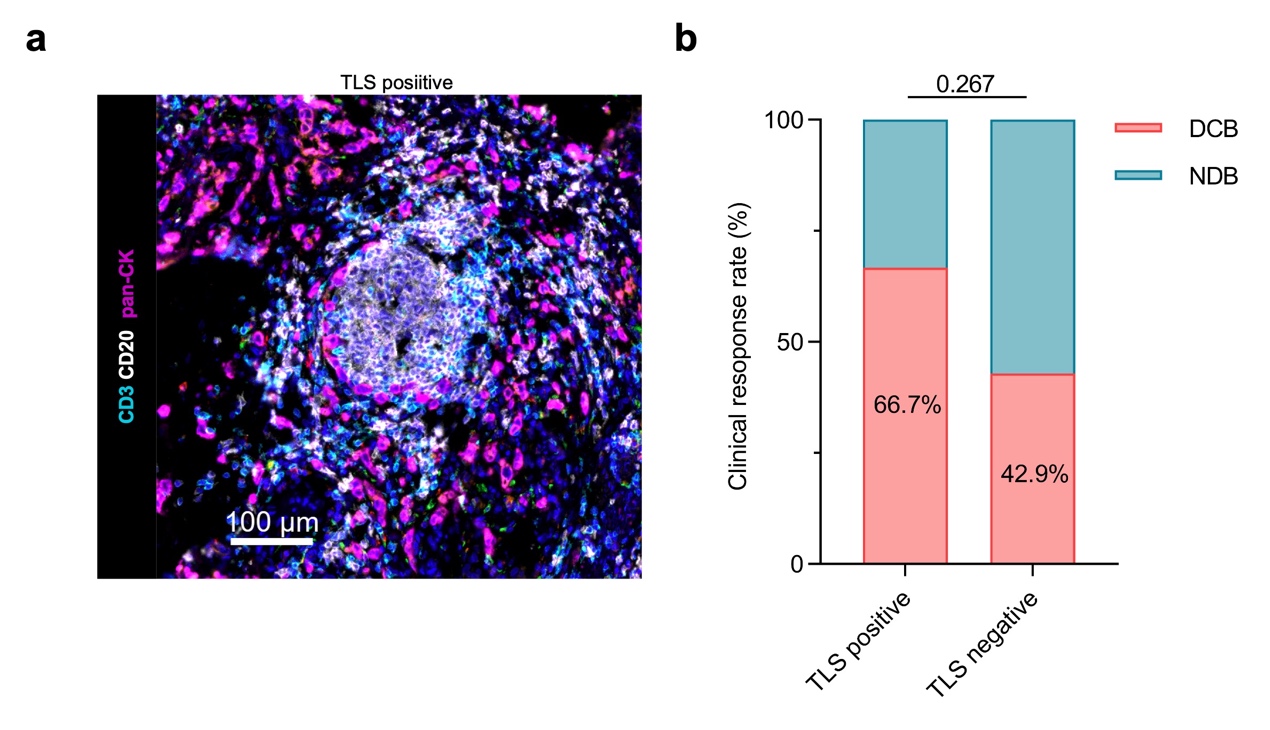
Figure S9. Correlation of tertiary lymphatic structures with therapeutic efficacy in AFP-G/GEJ adenocarcinoma. a,** Representative multiplex immunofluorescence images of TLSs. **b,** Association between durable clinical benefit and TLSs positive rate from pre-treatment (DCB, n=14; NDB, n=12). P values were calculated using the Fisher’s exact test.


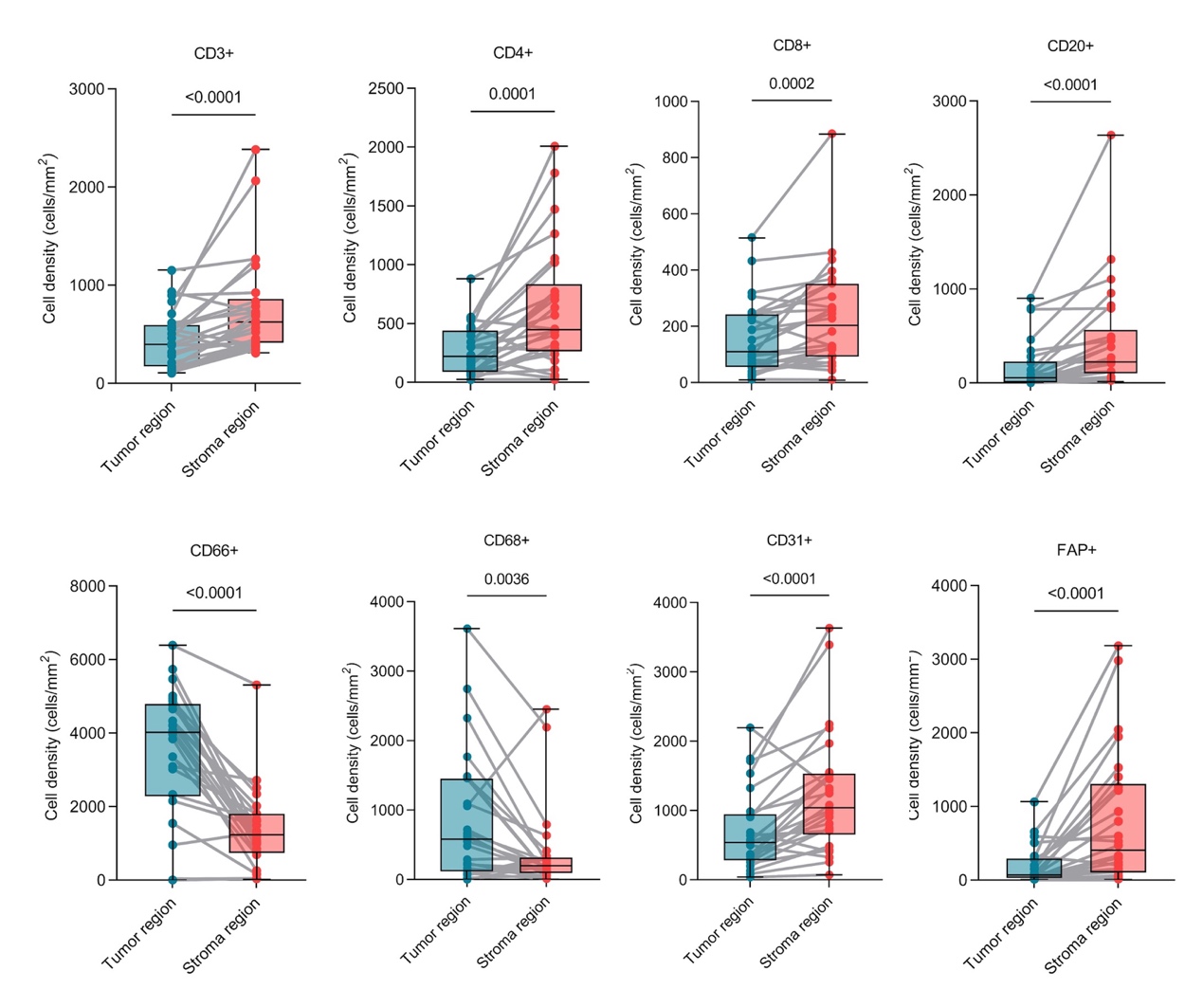


**Figure S10. The spatial distribution characteristics of AFP-G/GEJ adenocarcinoma.** The difference of density of tumor infiltrating cells between the tumor parenchyma and stroma region (n=26). P values were calculated using a paired two-sided Wilcoxon rank-sum test. The line in the middle of the box represents the median. The lower and the upper edges of the box are the 1st and 3rd quartiles, respectively. The dots are considered outliers, which are more than 1.5*IQR beyond the lower or upper quartiles.

**
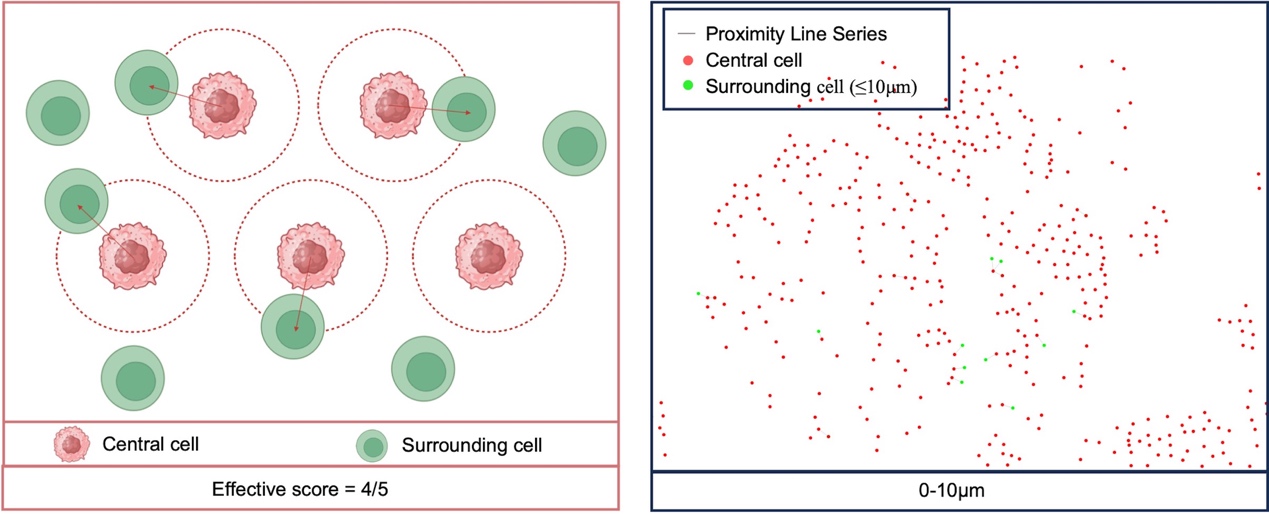
**

**Figure S11. Illustration of the distance analysis involving central cells and surrounding cells.** Red dots: central cells; green dots: surrounding cells. The red circle represents the radius. Effective score = number of paired central cells and surrounding cells/number of central cells.


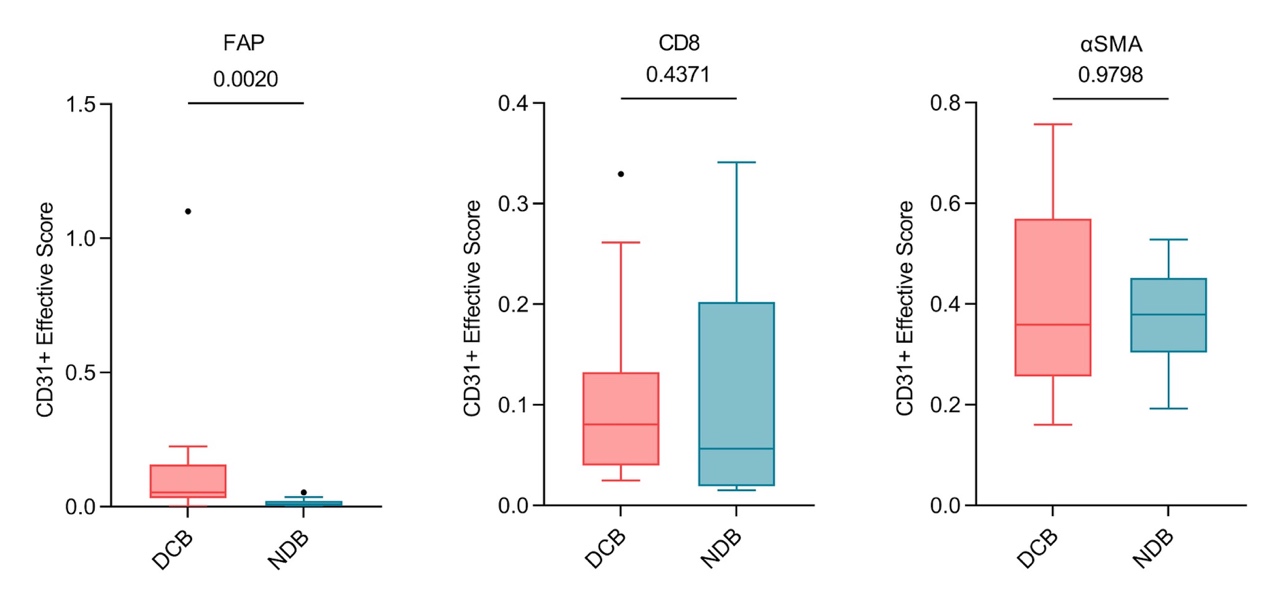


**Figure S12. The effective score of FAP^+^, CD8^+^ and α-SMA^+^ cells populations around CD31^+^cells in the tumour parenchyma in 10µm increments between DCB (n=14) and NDB (n=12).** P values were calculated using the two-sided Mann–Whitney U-test. The line in the middle of the box represents the median. The lower and the upper edges of the box are the 1st and 3rd quartiles, respectively. The dots are considered outliers, which are more than 1.5*IQR beyond the lower or upper quartiles.


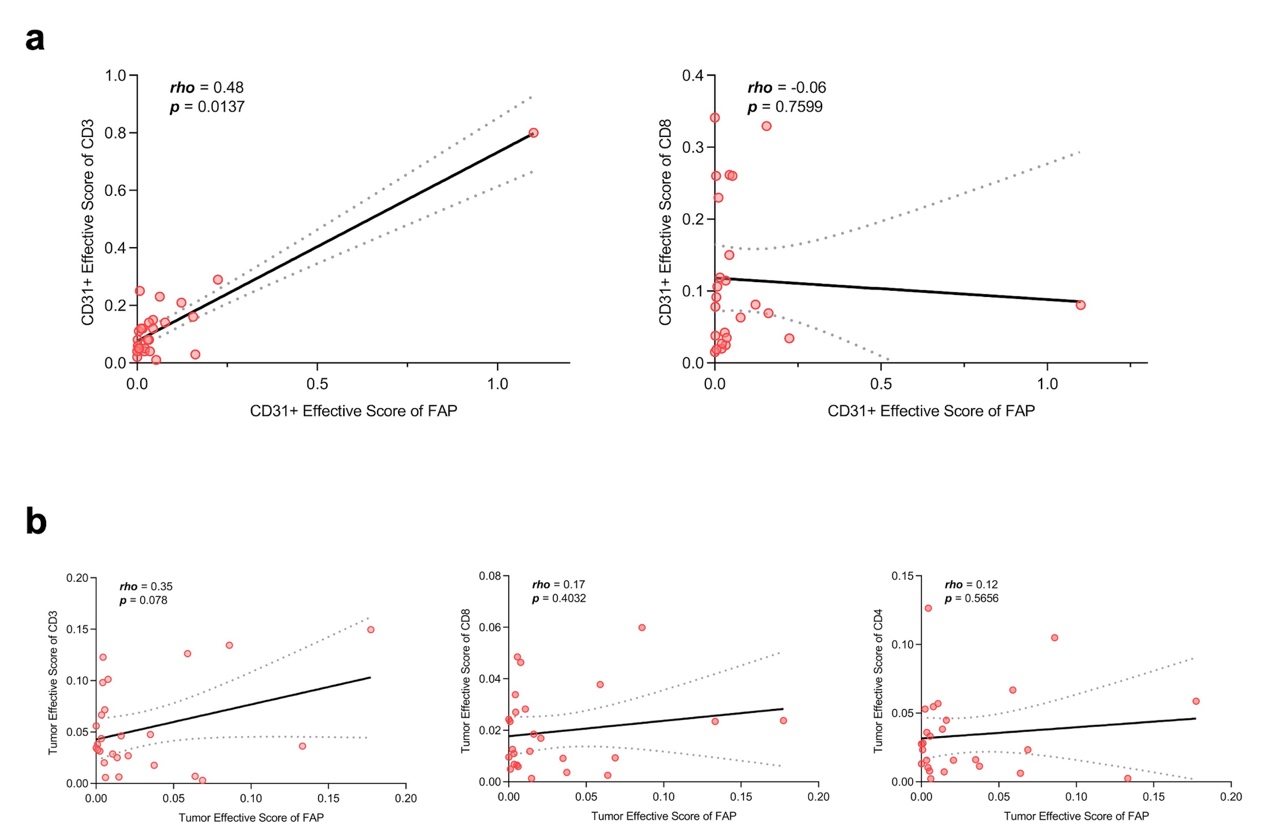


**Figure S13. Correlations between effective score of FAP and T cells.** **a,** Correlation between pretreatment CD31^+^ effective score of FAP and CD31^+^ effective score of CD3 and CD8 (n=26). **b,** Correlation between pretreatment tumor cells effective score of FAP and tumor cells effective score of CD3, CD8 and CD4 (n=26). The rho and p values of Spearman’s correlation as indicated.


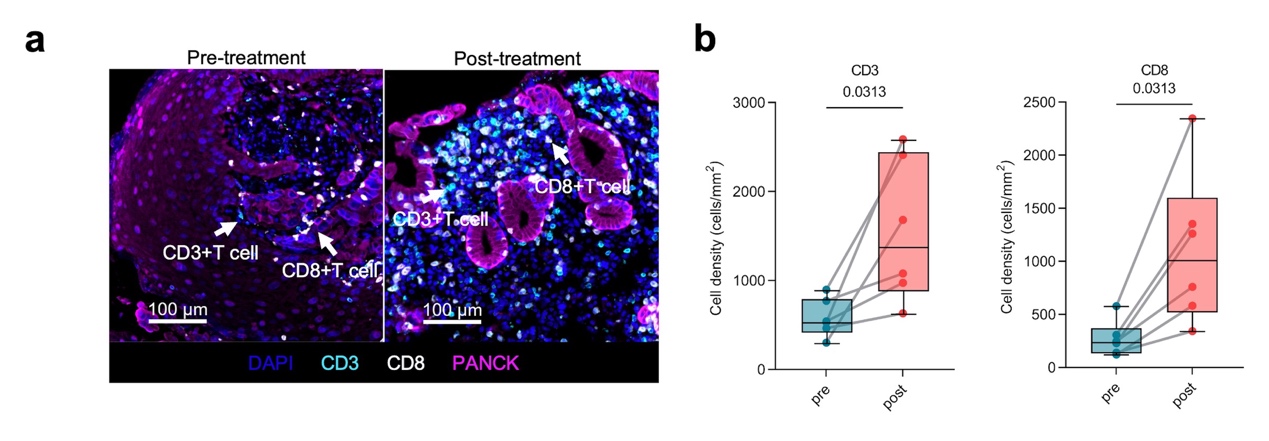


**Figure S14. Camrelizumab plus apatinib and SOX reshape the TME status of AFP-GC adenocarcinoma. a,** Representative multiplex immunofluorescence images of patients before and after treatment. **b,** Comparison of CD3^+^ and CD8^+^T cells density before and after treatment (n=6). P values were calculated using a paired two-sided Wilcoxon rank-sum test. The line in the middle of the box represents the median. The lower and the upper edges of the box are the 1st and 3rd quartiles, respectively. The dots are considered outliers, which are more than 1.5*IQR beyond the lower or upper quartiles.

**Table S1.** Subsequent anti-tumor therapies after the last dose of the study treatment.

|  | **All patients (n=36)** |
| --- | --- |
| **Any subsequent therapy** | 21 (58.3) |
| Chemotherapy | 16 (44.4) |
| Targeted therapy | 8 (22.2) |
| Anti-PD-1/PD-L1 therapy | 6 (16.7) |
| Radiotherapy | 2 (5.6) |
| Surgery | 2 (5.6) |
| Others* | 2 (5.6) |

Data are n (%). *One patient received second-line treatment with Claudin 18.2 ADC in a clinical trial, and another patient received second-line treatment with Disitamab Vedotin.

**Table S2.** Subgroup analysis of progression-free survival based on baseline characteristics

|  | Events/Patients | Median PFS (95% CI) |
| --- | --- | --- |
| Age |  |  |
| <65 years | 17/22 | 5.26 (4.83,12.29) |
| ≥65 years | 6/14 | 8.41 (4.17, NE) |
| Sex |  |  |
| Male | 18/30 | 8.84 (4.83, 14.06) |
| Female | 5/6 | 4.98 (1.28, NE) |
| ECOG PS |  |  |
| 0 | 13/20 | 8.84 (4.01, 14.32) |
| 1-2 | 10/16 | 5.16 (4.30, 12.29) |
| Primary tumor location |  |  |
| Gastric | 16/23 | 5.13 (4.17, 9.86) |
| GEJ | 7/13 | 12.29 (4.30, NE) |
| Lauren's classification |  |  |
| Intestinal | 13/18 | 7.79 (4.86, 14.06) |
| Non-intestinal | 5/13 | 5.16 (2.86, NE) |
| Number of organs with metastasis |  |  |
| 1-2 | 17/30 | 8.41 (5.03, 14.32) |
| >2 | 6/6 | 4.50 (1.22, NE) |
| Liver metastasis |  |  |
| Yes | 14/18 | 4.96 (3.65, 12.29) |
| No | 9/18 | 8.41 (5.03, NE) |
| Distal lymph node metastasis |  |  |
| Yes | 9/17 | 8.84 (5.13, NE) |
| No | 14/19 | 5.03 (4.01, 12.29) |
| Peritoneal metastasis |  |  |
| Yes | 8/9 | 5.13 (1.22, 7.79) |
| No | 15/27 | 8.84 (4.86, NE) |
| PVTT |  |  |
| Yes | 2/4 | NR (1.22, NE) |
| No | 21/32 | 7.79 (4.86, 12.29) |
| Serum AFP level |  |  |
| <400 | 11/14 | 8.41 (4.21, 14.06) |
| ≥400 | 12/22 | 5.26 (5.03, NE) |
| <800 | 13/18 | 6.77 (4.30, 14.06) |
| ≥800 | 10/18 | 6.52 (3.65, NE) |
| PD-L1 expression |  |  |
| CPS<1 | 8/14 | 10.92 (4.30, NE) |
| CPS≥1 | 12/18 | 5.26 (4.17, 12.29) |
| CPS<5 | 12/21 | 7.79 (4.86, NE) |
| CPS≥5 | 8/11 | 5.26 (4.01, NE) |

**Table S3.** Treatment-related adverse events occurring in ≥10% of all patients and immune-related adverse events occurring in all patients.

| Events, n (%) |  | All patients (n=36) | | | |
| --- | --- | --- | --- | --- | --- |
|  | Any grade | Grade 1-2 | | Grade≥3 | |
| Any treatment-related adverse events | 34 (94.4) | 18 (50.0) | 16 (44.4) | |  |
| Neutrophil count decreased | 20 (55.6) | 14 (38.9) | 6 (16.7) | |  |
| White blood cell count decreased | 18 (50.0) | 17 (47.2) | 1 (2.8) | |  |
| Anemia | 12 (33.3) | 10 (27.8) | 2 (5.6) | |  |
| Platelet count decreased | 11 (30.6) | 9 (25.0) | 2 (5.6) | |  |
| Alanine aminotransferase increased | 9 (25.0) | 9 (25.0) | 0 | |  |
| Reactive cutaneous capillary endothelial proliferation | 8 (22.2) | 8 (22.2) | 0 | |  |
| Diarrhea | 8 (22.2) | 6 (16.7) | 2 (5.6) | |  |
| Aspartate aminotransferase increased | 8 (22.2) | 8 (22.2) | 0 | |  |
| Hypertension | 7 (19.4) | 4 (11.1) | 3 (8.3) | |  |
| Proteinuria | 5 (13.9) | 5 (13.9) | 0 | |  |
| Amylase increased | 5 (13.9) | 5 (13.9) | 0 | |  |
| Bilirubin increased | 5 (13.9) | 5 (13.9) | 0 | |  |
| Nausea | 4 (11.1) | 4 (11.1) | 0 | |  |
| Hyperthyroidism | 4 (11.1) | 4 (11.1) | 0 | |  |
| Protein detected in urine | 4 (11.1) | 4 (11.1) | 0 | |  |
| Any immune-related adverse events | 18 (50.0) | 15 (41.7) | 3 (8.3) | |  |
| Reactive cutaneous capillary endothelial proliferation | 8 (22.2) | 8 (22.2) | 0 | |  |
| Amylase increased | 5 (13.9) | 5 (13.9) | 0 | |  |
| Hyperthyroidism | 4 (11.1) | 4 (11.1) | 0 | |  |
| Hypothyroidism | 3 (8.3) | 3 (8.3) | 0 | |  |
| Diarrhea | 2 (5.6) | 1 (2.8) | 1 (2.8) | |  |
| Creatine kinase increased | 2 (5.6) | 2 (5.6) | 0 | |  |
| Rash | 2 (5.6) | 2 (5.6) | 0 | |  |
| Alanine aminotransferase increased | 1 (2.8) | 1 (2.8) | 0 | |  |
| Blood thyroid stimulating hormone decreased | 1 (2.8) | 1 (2.8) | 0 | |  |
| Blood thyroid stimulating hormone increased | 1 (2.8) | 1 (2.8) | 0 | |  |
| Bilirubin increased | 1 (2.8) | 1 (2.8) | 0 | |  |
| Platelet count decreased | 1 (2.8) | 0 | 1 (2.8) | |  |
| Lipase increased | 1 (2.8) | 0 | 1 (2.8) | |  |

**Table S4**. Treatment-related adverse events leading to dose adjustment

| Events, n (%) | All patients (n=36) | | |
| --- | --- | --- | --- |
|  | Any grade | Grade 1-2 | Grade ≥3 |
| TRAEs leading to treatment discontinuation of chemotherapy | 1 (2.8) | 0 | 1 (2.8) |
| White blood cell count decreased | 1 (2.8) | 1 (2.8) | 0 |
| Platelet count decreased | 1 (2.8) | 1 (2.8) | 0 |
| Neutrophil count decreased | 1 (2.8) | 0 | 1 (2.8) |
| TRAEs leading to treatment discontinuation of camrelizumab | 1 (2.8) | 0 | 1 (2.8) |
| Platelet count decreased | 1 (2.8) | 0 | 1 (2.8) |
| TRAEs leading to treatment discontinuation of apatinib | 1 (2.8) | 1 (2.8) | 0 |
| Fatigue | 1 (2.8) | 1 (2.8) | 0 |
| TRAEs leading to delay or interruption of chemotherapy | 12 (33.3) | 6 (16.7) | 6 (16.7) |
| Platelet count decreased | 7 (19.4) | 6 (16.7) | 1 (2.8) |
| Neutrophil count decreased | 6 (16.7) | 4 (11.1) | 2 (5.6) |
| White blood cell count decreased | 3 (8.3) | 2 (5.6) | 1 (2.8) |
| Anemia | 2 (5.6) | 1 (2.8) | 1 (2.8) |
| Gamma-glutamyltransferase increased | 1 (2.8) | 0 | 1 (2.8) |
| Alanine aminotransferase increased | 1 (2.8) | 1 (2.8) | 0 |
| Fatigue | 1 (2.8) | 1 (2.8) | 0 |
| Abdominal pain | 1 (2.8) | 1 (2.8) | 0 |
| Diarrhea | 1 (2.8) | 0 | 1 (2.8) |
| Abdominal distention | 1 (2.8) | 1 (2.8) | 0 |
| Vomiting | 1 (2.8) | 0 | 1 (2.8) |
| Numbness in limbs | 1 (2.8) | 1 (2.8) | 0 |
| Dizziness | 1 (2.8) | 1 (2.8) | 0 |
| TRAEs leading to dose delay of camrelizumab | 10 (27.8) | 3 (8.3) | 7 (19.4) |
| Platelet count decreased | 5 (13.9) | 4 (11.1) | 1 (2.8) |
| Neutrophil count decreased | 5 (13.9) | 2 (5.6) | 3 (8.3) |
| White blood cell count decreased | 2 (5.6) | 1 (2.8) | 1 (2.8) |
| Gamma-glutamyltransferase increased | 1 (2.8) | 0 | 1 (2.8) |
| Alanine aminotransferase increased | 1 (2.8) | 1 (2.8) | 0 |
| Vomiting | 1 (2.8) | 0 | 1 (2.8) |
| Anemia | 1 (2.8) | 0 | 1 (2.8) |
| TRAEs leading to dose interruption of apatinib | 7 (19.4) | 2 (5.6) | 5 (13.9) |
| White blood cell count decreased | 3 (8.3) | 2 (5.6) | 1 (2.8) |
| Neutrophil count decreased | 3 (8.3) | 1 (2.8) | 2 (5.6) |
| Platelet count decreased | 2 (5.6) | 2 (5.6) | 0 |
| Gamma-glutamyltransferase increased | 1 (2.8) | 0 | 1 (2.8) |
| Hypoalbuminemia | 1 (2.8) | 1 (2.8) | 0 |
| Abdominal pain | 1 (2.8) | 1 (2.8) | 0 |
| Diarrhea | 1 (2.8) | 0 | 1 (2.8) |
| Abdominal distention | 1 (2.8) | 1 (2.8) | 0 |
| Hypertension | 1 (2.8) | 0 | 1 (2.8) |
| Vomiting | 1 (2.8) | 0 | 1 (2.8) |
| Rash | 1 (2.8) | 1 (2.8) | 0 |
| Anemia | 1 (2.8) | 0 | 1 (2.8) |
| TRAEs leading to dose reduction of chemotherapy | 7 (19.4) | 4 (11.1) | 3 (8.3) |
| Platelet count decreased | 3 (8.3) | 3 (8.3) | 0 |
| Neutrophil count decreased | 2 (5.6) | 0 | 2 (5.6) |
| White blood cell count decreased | 1 (2.8) | 0 | 1 (2.8) |
| Hyponatremia | 1 (2.8) | 0 | 1 (2.8) |
| Nausea | 1 (2.8) | 1 (2.8) | 0 |
| TRAEs leading to dose reduction of apatinib | 2 (5.6) | 2 (5.6) | 0 |
| Gingival bleeding | 1 (2.8) | 1 (2.8) | 0 |
| PPES | 1 (2.8) | 1 (2.8) | 0 |

TRAEs, treatment-related adverse events. PPES, palmar-plantar erythrodysesthesia syndrome.

**Table S5.** The distribution of tumor-infiltrating cells density across LRP1B status

|  | LRP1B status | |  |
| --- | --- | --- | --- |
|  | LRP1B mutation | LRP1B wild type | *P* value |
|  | (N=4) | (N=21) |  |
| CD3^+^ | 327.7±45.9 | 642.9±459.9 | 0.15 |
| **CD8^+^** | **60.8±38.0** | **204.3±117.7** | **0.01** |
| **CD8^+^PD1^+^** | **10.0±8.5** | **56.4±46.1** | **0.04** |
| CD8^+^LAG3^+^ | 4.7±8.9 | 9.8±19.1 | 0.16 |
| CD8^+^CD137^+^ | 4.9±1.3 | 23.0±25.6 | 0.26 |
| CD8^+^GZMB^+^ | 3.4±2.2 | 7.2±8.6 | 0.59 |
| CD4^+^ | 222.6±96.3 | 378.7±267.3 | 0.33 |
| CD4^+^FOXP3^+^ | 18.4±8.7 | 70.7±111.3 | 0.59 |
| CD4^+^FOXP3^+^CTLA4^+^ | 1.9±1.2 | 8.2±17.8 | 0.69 |
| CD4^+^PDL1^+^ | 29.0±34.8 | 101.7±96.1 | 0.11 |
| CD20^+^ | 103.5±103.8 | 362.2±372.7 | 0.11 |
| CD66^+^ | 2927±1954 | 1763±1186 | 0.20 |
| CD68^+^ | 638.9±771.1 | 419.0±472.4 | 0.37 |
| CD68^+^CD163^-^ | 616.3±753.5 | 396.1±459.5 | 0.45 |
| CD68^+^CD163^+^ | 22.6±18.2 | 22.9±21.9 | 0.80 |
| CD31^+^ | 742.3±338.1 | 916.5±759.7 | 0.86 |
| VEGFR2^+^ | 329.7±289.6 | 158.5±218.0 | 0.13 |
| FAP^+^ | 410.7±388.6 | 426.5±479.2 | 0.59 |
| a-SMA^+^ | 2073±626.5 | 2053±957.8 | 0.75 |

^a^Two-sided Mann-Whitney U test. ^b^Data presented with Mean ± SD.

**Table S6.** The distribution of tumor-infiltrating cells density across PI3K pathway status

|  | PI3K pathway status | |  |
| --- | --- | --- | --- |
|  | PI3K pathway altered | PI3K pathway wild type | *P* value |
|  | (N=4) | (N=21) |  |
| **CD3^+^** | **324.0±204.0** | **643.6±452.9** | **0.047** |
| **CD8^+^** | **72.9±16.5** | **202.0±121.2** | **0.02** |
| CD8^+^PD1^+^ | 17.2±13.6 | 55.0±47.2 | 0.20 |
| CD8^+^LAG3^+^ | 0.9±0.8 | 10.5±19.1 | 0.13 |
| CD8^+^CD137^+^ | 5.7±2.5 | 22.8±25.7 | 0.41 |
| CD8^+^GZMB^+^ | 5.3±2.4 | 6.9±8.7 | 0.45 |
| **CD4^+^** | **145.7±80.2** | **393.4±256.3** | **0.02** |
| CD4^+^FOXP3^+^ | 12.8±12.7 | 71.8±110.6 | 0.16 |
| **CD4^+^FOXP3^+^CTLA4^+^** | **0.3±0.4** | **8.5±17.7** | **0.046** |
| CD4^+^PDL1^+^ | 34.2±40.3 | 100.7±96.6 | 0.23 |
| CD20^+^ | 123.1±207.1 | 358.4±368.8 | 0.15 |
| CD66^+^ | 3056±1213 | 1738±1302 | 0.06 |
| CD68^+^ | 151.1±55.2 | 511.9±546.0 | 0.15 |
| CD68^+^CD163^-^ | 143.0±50.2 | 486.2±533.2 | 0.20 |
| CD68^+^CD163^+^ | 8.1±5.3 | 25.7±21.7 | 0.07 |
| CD31^+^ | 566.0±117.4 | 950.0±756.7 | 0.50 |
| VEGFR2^+^ | 156.1±131.2 | 191.5±249.4 | 0.69 |
| FAP^+^ | 412.7±640.1 | 426.1±436.8 | 0.75 |
| a-SMA^+^ | 1704±506.1 | 2124±953.1 | 0.50 |

^a^Two-sided Mann-Whitney U test. ^b^Data presented with Mean ± SD.

**Table S7.** The distribution of tumor-infiltrating cells density in tumor parenchyma across efficacy

|  | Efficacy | |  |  |  |
| --- | --- | --- | --- | --- | --- |
|  | DCB | NDB |  | *P* value |  |
|  | (N=14) | （N=12) |  |  |  |
| CD3^+^ | 529.8±257.3 | 348.5±297.7 |  | 0.07 |  |
| **CD8^+^** | **200.8±129.1** | **115.0±120.8** |  | **0.04** |  |
| **CD8^+^PD1^+^** | **57.9±48.1** | **22.8±25.4** |  | **0.02** |  |
| CD8^+^LAG3^+^ | 10.2±19.5 | 5.1±9.7 |  | 0.14 |  |
| CD8^+^CD137^+^ | 23.2±28.0 | 14.5±17.4 |  | 0.35 |  |
| CD8^+^GZMB^+^ | 9.3±9.1 | 4.5±3.6 |  | 0.38 |  |
| CD4^+^ | 297.1±198.4 | 247.5±235.0 |  | 0.37 |  |
| CD4^+^FOXP3^+^ | 60.1±72.0 | 29.9±52.1 |  | 0.09 |  |
| CD4^+^FOXP3^+^CTLA4^+^ | 13.6±26.5 | 3.9±9.0 |  | 0.14 |  |
| CD4^+^PDL1^+^ | 79.9±72.0 | 62.9±90.4 |  | 0.40 |  |
| CD20^+^ | 186.3±291.0 | 158.2±251.1 |  | 0.21 |  |
| CD66^+^ | 2956±1919 | 4118±1456 |  | 0.12 |  |
| CD68^+^ | 951.6±1070 | 722.9±771.0 |  | 0.78 |  |
| CD68^+^CD163^-^ | 915.8±1045 | 699.8±749.5 |  | 0.78 |  |
| CD68^+^CD163^+^ | 35.7±34.8 | 23.1±23.9 |  | 0.44 |  |
| CD31^+^ | 810.4±631.8 | 558.7±485.8 |  | 0.23 |  |
| VEGFR2^+^ | 197.5±217.1 | 345.6±420.2 |  | 0.56 |  |
| **FAP^+^** | **288.3±312.3** | **65.1±83.4** |  | **0.04** |  |
| a-SMA^+^ | 1821±1227 | 1995±577.4 |  | 0.32 |  |

^a^Two-sided Mann-Whitney U test. ^b^Data presented with Mean ± SD.

**Table S8.** The distribution of tumor-infiltrating cells density in stroma region across efficacy

|  | Efficacy | |  |  |
| --- | --- | --- | --- | --- |
|  | DCB | NDB |  | *P* value |
|  | (N=14) | (N=12) |  |  |
| CD3^+^ | 908.4±632.3 | 597.9±254.5 |  | 0.21 |
| **CD8^+^** | **303.5±208.1** | **153.3±120.1** |  | **0.03** |
| **CD8^+^PD1^+^** | **88.1±71.0** | **22.8±25.4** |  | **0.01** |
| **CD8^+^LAG3^+^** | **15.9±25.9** | **1.8±2.6** |  | **0.01** |
| CD8^+^CD137^+^ | 22.8±28.9 | 10.4±11.3 |  | 0.82 |
| CD8^+^GZMB^+^ | 3.7±4.0 | 2.8±3.0 |  | 0.74 |
| CD4^+^ | 705.6±621.4 | 534.9±404.9 |  | 0.60 |
| CD4^+^FOXP3^+^ | 150.2±245.7 | 73.9±99.6 |  | 0.59 |
| CD4^+^FOXP3^+^CTLA4^+^ | 13.4±21.4 | 3.7±4.4 |  | 0.21 |
| CD4^+^PDL1^+^ | 164.9±201.2 | 97.3±111.5 |  | 0.43 |
| CD20^+^ | 562.4±686.8 | 314.7±389.3 |  | 0.19 |
| CD66^+^ | 1063±827.2 | 1716±1295 |  | 0.23 |
| CD68^+^ | 408.0±557.6 | 351.4±669.3 |  | 0.37 |
| CD68^+^CD163^-^ | 381.4±556.1 | 326.7±660.0 |  | 0.40 |
| CD68^+^CD163^+^ | 26.6±26.7 | 24.7±23.5 |  | 0.90 |
| CD31^+^ | 1366±1064 | 1095±621.1 |  | 0.74 |
| VEGFR2^+^ | 170.9±216.6 | 204.3±194.6 |  | 0.25 |
| FAP^+^ | 923.0±926.3 | 630.1±917.4 |  | 0.18 |
| a-SMA^+^ | 2624±1056 | 2708±1254 |  | 0.78 |

^a^Two-sided Mann-Whitney U test. ^b^Data presented with Mean ± SD.
