# Supplementary material for: PD-1 antibody camrelizumab plus apatinib and SOX as first-line treatment in patients with AFP-producing gastric or gastro-esophageal junction adenocarcinoma (CAP 06): a multi-center, single-arm, phase 2 trial
Source: Signal Transduct Target Ther. 2025 Mar 14;10:100. doi: 10.1038/s41392-025-02193-z (PMC11906745; doi:10.1038/s41392-025-02193-z)
Supplement: Supplementary file 2 — Study protocol [file 41392_2025_2193_MOESM2_ESM.docx]

A prospective, nonrandomized, multicenter phase II clinical trial of camrelizumab combined with apatinib for unresectable/recurrent or metastatic AFP-producing gastric/gastroesophageal junction adenocarcinoma

**Study protocol**

Project/Protocol ID: MA-GC-II-007

Version number: version 2.0

Date: December 2, 2022

Sponsor: Peking University Cancer Hospital

Organization of clinical trial team leader: Peking University Cancer Hospital

Project leader: Xiaotian Zhang

Department: Department of Digestive Oncology

Tel: 010-88196747

Project execution period: June 2020 to March 2024

**Version history/Revision history**

| File version | Version date | Revision record |
| --- | --- | --- |
| v1.1 | September 16, 2020 | None |
| v2.0 | December 2, 2022 | Modify the sample size calculation  Adjust study execution time according to progress |

**Protocol signature page**

We have read and confirmed this clinical trial protocol (protocol number: MA-GC-II-007, version number: 2.0, date: 2022/12/02). I agree to perform relevant responsibilities in accordance with ICH-GCP, any applicable laws and regulations, and this trial protocol.

**Applicant:** Beijing Cancer Hospital

| Xiaotian Zhang |  |  |
| --- | --- | --- |
| Principal Investigator (print) | Principal investigator (signature) | Date of signature (year/month/day) |

**Principal investigator’s signature page (team leader unit)**

I will conscientiously perform my duties as an investigator in accordance with the ICH-GCP regulations and will personally participate or directly guide this clinical study. I have received the Investigator’s brochure for the drug in this clinical trial; I have known and read the preclinical study of this trial drug and the study protocol of this clinical trial. I agree to perform relevant responsibilities in accordance with ICH-GCP, the Declaration of Helsinki, any applicable laws and regulations, and this trial protocol. Unless measures must be taken to protect the safety, rights, and interests of the subjects, I will only modify the protocol after notifying the sponsor and obtaining consent and then implement the changes after obtaining approval from the ethics committee. I will be responsible for making clinically relevant medical decisions, ensuring that subjects can receive timely and appropriate treatment when adverse events occur during the trial, and recording and reporting these adverse events in accordance with relevant national regulations. I promise to record the data in a true, accurate, complete, and timely manner. I will accept the supervision and audit by the monitor or inspectors dispatched by the sponsor and the inspection by the drug regulatory department to ensure the quality of clinical trials. I promise to keep the subject’s personal information and related matters confidential. I agree to disclose my full name and occupation to the sponsor, agree to disclose the expenses related to clinical trial upon request, and agree to the prohibition of commercial and economic behaviors related to this trial. I agree with the use of the trial results in drug registration and publication.

**Research organization:** Peking University Cancer Hospital

| Xiaotian Zhang |  |  |
| --- | --- | --- |
| Principal Investigator (print) | Principal investigator (signature) | Date of signature (year/month/day) |

**Principal investigator’s signature page (participating units)**

I will conscientiously perform my duties as an investigator in accordance with the ICH-GCP regulations and will personally participate or directly guide this clinical study. I have received the Investigator’s brochure for the drug in this clinical trial; I have known and read the preclinical study of this trial drug and the study protocol of this clinical trial. I agree to perform relevant responsibilities in accordance with ICH-GCP, the Declaration of Helsinki, any applicable laws and regulations, and this trial protocol. Unless measures must be taken to protect the safety, rights, and interests of the subjects, I will only modify the protocol after notifying the sponsor and obtaining consent and then implement the changes after obtaining approval from the ethics committee. I will be responsible for making clinically relevant medical decisions, ensuring that subjects can receive timely and appropriate treatment when adverse events occur during the trial, and recording and reporting these adverse events in accordance with relevant national regulations. I promise to record the data in a true, accurate, complete, and timely manner. I will accept the supervision and audit by the monitor or inspectors dispatched by the sponsor and the inspection by the drug regulatory department to ensure the quality of clinical trials. I promise to keep the subject’s personal information and related matters confidential. I agree to disclose my full name and occupation to the sponsor, agree to disclose the expenses related to clinical trial upon request, and agree to the prohibition of commercial and economic behaviors related to this trial. I agree with the use of the trial results in drug registration and publication.

**Research organization:** __________________________________________

|  |  |  |
| --- | --- | --- |
| Principal Investigator (print) | Principal investigator (signature) | Date of signature (year/month/day) |

**Table of** **Contents**

[Trial flow chart 9](#_Toc170141755)

[Protocol summary 17](#_Toc170141756)

[Abbreviations 29](#_Toc170141757)

[1. Research Background and Scientific Evidence 32](#_Toc170141758)

[1.1. Research Background 32](#_Toc170141759)

[1.1.1. Overview of gastric cancer (GC) 32](#_Toc170141760)

[1.1.2. Alpha-fetoprotein-producing gastric cancer (AFPGC) 32](#_Toc170141761)

[1.1.3. Exploration of apatinib and programmed cell death protein 1 (PD-1) monoclonal antibody (mAb) in AFPGC 33](#_Toc170141762)

[1.1.4. Risk/benefit assessment of this trial 36](#_Toc170141763)

[2. Research objectives and study endpoints 37](#_Toc170141764)

[3. Clinical trial design 39](#_Toc170141765)

[3.1. Description of the clinical trial design 39](#_Toc170141766)

[3.1.1. Determination of sample size 39](#_Toc170141767)

[3.2. Overall design 40](#_Toc170141768)

[3.2.1. Study Treatment 40](#_Toc170141769)

[3.2.2. Imaging evaluation 41](#_Toc170141770)

[3.2.3. Survival follow-up 42](#_Toc170141771)

[3.2.4. Safety follow-up 42](#_Toc170141772)

[3.2.5. Sample collection 42](#_Toc170141773)

[3.2.6. Trial design chart 43](#_Toc170141774)

[4. Subject selection and withdrawal from the trial 44](#_Toc170141775)

[4.1. Inclusion criteria 44](#_Toc170141776)

[4.2. Exclusion criteria 45](#_Toc170141777)

[4.3. Withdrawal from the trial or termination of the treatment 48](#_Toc170141778)

[4.3.1. Criteria for termination of trial treatment 48](#_Toc170141779)

[4.3.2. Criteria for withdrawal from the trial 48](#_Toc170141780)

[4.3.3. Steps to withdraw from the trial or terminate the treatment 49](#_Toc170141781)

[4.3.4. Loss to follow-up 50](#_Toc170141782)

[4.4. Early termination or suspension of the trial 50](#_Toc170141783)

[4.5. Definition of end of the trial 50](#_Toc170141784)

[5. Trial drugs 51](#_Toc170141785)

[5.1. Overview of the trial drugs 51](#_Toc170141786)

[5.1.1. Drug storage and stability 51](#_Toc170141787)

[5.1.2. Dosage adjustment 52](#_Toc170141788)

[5.2. Drug management, distribution and return 62](#_Toc170141789)

[5.3. Combined medication/concomitant treatment 62](#_Toc170141790)

[5.3.1. Prohibited drugs and treatments and drugs allowed with caution during the clinical trial 63](#_Toc170141791)

[5.3.2. Combined drugs and treatments allowed during the trial 64](#_Toc170141792)

[5.3.3. Drugs that should be used with caution in patients who received apatinib treatment during the trial 65](#_Toc170141793)

[5.4. Suggestions for symptomatic treatment of common adverse reactions to camrelizumab 66](#_Toc170141794)

[5.4.1. Immuno-oncological drug safety management rules 67](#_Toc170141795)

[5.4.2. Infusion reactions 67](#_Toc170141796)

[5.4.3. Grading criteria and therapy recommendations for camrelizumab-induced reactive cutaneous capillary endothelial proliferation 70](#_Toc170141797)

[6. Research procedures 70](#_Toc170141798)

[6.1. Screening period 71](#_Toc170141799)

[6.2. Treatment period and end of treatment 73](#_Toc170141800)

[6.3. Follow-up period 75](#_Toc170141801)

[6.4. Unplanned visits 76](#_Toc170141802)

[7. Evaluation 77](#_Toc170141803)

[7.1. Efficacy evaluation and analysis 77](#_Toc170141804)

[7.2. Safety evaluation 79](#_Toc170141805)

[7.2.1. Adverse events 79](#_Toc170141806)

[7.2.2. Pregnancy test 79](#_Toc170141807)

[7.2.3. ECOG PS score 79](#_Toc170141808)

[8. Adverse event report 79](#_Toc170141809)

[8.1. Adverse events (AEs) 79](#_Toc170141810)

[8.1.1. Definition of AEs 79](#_Toc170141811)

[8.1.2. Criteria for determining the severity of AEs 80](#_Toc170141812)

[8.1.3. Criteria for determining the relationship between AEs and trial drugs 81](#_Toc170141813)

[8.2. SAEs 81](#_Toc170141814)

[8.2.1. Definition of SAEs 81](#_Toc170141815)

[8.2.2. Hospitalization 81](#_Toc170141816)

[8.2.3. Disease progression and death 82](#_Toc170141817)

[8.2.4. Other new antitumor treatments 83](#_Toc170141818)

[8.2.5. Abnormal liver function test 83](#_Toc170141819)

[8.2.6. Reporting of SAEs 84](#_Toc170141820)

[8.3. Collection and follow-up period of AEs/SAEs 84](#_Toc170141821)

[8.4. Pregnancy 85](#_Toc170141822)

[8.5. Reporting of nonserious AEs and other special conditions 85](#_Toc170141823)

[9. Trial management 86](#_Toc170141824)

[9.1. Ethics standards and informed consent 86](#_Toc170141825)

[9.2. Protocol revision 87](#_Toc170141826)

[9.3. Quality assurance of clinical trials 87](#_Toc170141827)

[9.4. Data management 87](#_Toc170141828)

[9.4.1. Data collection 87](#_Toc170141829)

[9.4.2. Data management and quality control 88](#_Toc170141830)

[9.4.3. Maintenance of trial records 88](#_Toc170141831)

[10. Data analysis/Statistical methods 89](#_Toc170141832)

[10.1. Analysis Population 89](#_Toc170141833)

[10.2. Statistical methods 89](#_Toc170141834)

[10.2.1. General method 89](#_Toc170141835)

[10.2.2. Subject disposition 90](#_Toc170141836)

[10.2.3. Demographics and baseline characteristics 90](#_Toc170141837)

[10.2.4. Primary Efficacy Analysis 90](#_Toc170141838)

[10.2.5. Secondary Efficacy Analysis 90](#_Toc170141839)

[10.2.6. Safety Analysis 91](#_Toc170141840)

[10.2.7. Exploratory endpoint analysis: 91](#_Toc170141841)

[10.2.8. Previous and concomitant medications 92](#_Toc170141842)

[11. Data management methods 92](#_Toc170141843)

[11.1. Data recording 92](#_Toc170141844)

[11.1.1. Filling in of original medical records and records 92](#_Toc170141845)

[11.1.2. Fill-in eCRFs 92](#_Toc170141846)

[11.1.3. eCRF audit 92](#_Toc170141847)

[11.2. Data management 93](#_Toc170141848)

[11.2.1. Establishing EDC database 93](#_Toc170141849)

[11.2.2. Data auditing and database locking 93](#_Toc170141850)

[11.2.3. Data archiving 93](#_Toc170141851)

[12. Raw data and raw documents 93](#_Toc170141852)

[13. Quality Assurance and quality Control 94](#_Toc170141853)

[14. Regulatory ethics, informed consent and protection of subjects 95](#_Toc170141854)

[14.1. Regulatory considerations 95](#_Toc170141855)

[14.2. Ethical norms 95](#_Toc170141856)

[14.3. Independent ethics committee 96](#_Toc170141857)

[14.4. Informed consent 96](#_Toc170141858)

[14.4.1. Informed consent and other written information required by the subjects 96](#_Toc170141859)

[14.4.2. Informed consent process and records 96](#_Toc170141860)

[14.5. Confidentiality of subject information 97](#_Toc170141861)

[15. Publication of trial results 98](#_Toc170141862)

[16. Clinical trial progress 98](#_Toc170141863)

[17. References 98](#_Toc170141864)

# Trial flow chart

| **Project/assessment time** | **Screening period** | | **Treatment period**  **（21 days for 1 treatment cycle）** | | | | | **Treatment**  **End** | **Follow-up period** | |
| --- | --- | --- | --- | --- | --- | --- | --- | --- | --- | --- |
|  | **-21d** | **-7d** | **Cycle 1^[1]^** | **Cycle 2** | **Cycle 3** | **Cycle 4** | **Cycle n** |  | **Safety follow-up** | **Tumor progression and survival follow-up** |
|  |  |  | **Day 1** | **Day 1** | **Day 1** | **Day 1** | **Day 1** |  |  |  |
|  |  |  |  | （±3d） | （±3d） | （±3d） | （±3d） | （±7d） |  |  |
| Baseline information | | | | | | | | | | |
| Sign informed consent | **×** |  |  |  |  |  |  |  |  |  |
| Demographic information | **×** |  |  |  |  |  |  |  |  |  |
| Medical history of the tumor^[2]^ | **×** |  |  |  |  |  |  |  |  |  |
| Other medical history^[3]^ | **×** |  |  |  |  |  |  |  |  |  |
| Medication history^[4]^ | **×** |  |  |  |  |  |  |  |  |  |
| Combined medication and concomitant therapy^[5]^ | **×** | **×** | **×** | **×** | **×** | **×** | **×** | **×** | **×** |  |
| Laboratory examination | | | | | | | | | | |
| Complete blood count^[6]^ |  | **×** | **×** | **×** | **×** | **×** | **×** | **×** |  |  |
| Blood biochemistry ^[7]^ |  | **×** | **×** | **×** | **×** | **×** | **×** | **×** |  |  |
| AFP^[8]^ |  | **×** | **×** | **×** | **×** | **×** | **×** | **×** |  |  |
| Serum amylase test ^[9]^ |  | **×** | **×** | **×** | **×** | **×** | **×** | **×** |  |  |
| Urinalysis ^[10]^ |  | **×** | **×** | **×** | **×** | **×** | **×** | **×** |  |  |
| Fecal occult blood test ^[11]^ |  | **×** | when necessary | | | | | | |  |
| Coagulation function test ^[12]^ |  | **×** | **×** | **×** | **×** | **×** | **×** | **×** |  |  |
| Thyroid function test ^[13]^ | **×** |  |  | **×** | **×** | **×** | **×** | **×** |  |  |

| **Project/assessment time** | **Screening period** | | **Treatment period**  **（21 days for 1 treatment cycle）** | | | | | **Treatment**  **End** | **Follow-up period** | |
| --- | --- | --- | --- | --- | --- | --- | --- | --- | --- | --- |
|  | **-21d** | **-7d** | **Cycle 1^[1]^** | **Cycle 2** | **Cycle 3** | **Cycle 4** | **Cycle n** |  | **Safety follow-up** | **Tumor progression and survival follow-up** |
|  |  |  | **Day 1** | **Day 1** | **Day 1** | **Day 1** | **Day 1** |  |  |  |
|  |  |  |  | （±3d） | （±3d） | （±3d） | （±3d） | （±7d） |  |  |
| Hepatitis B and C screening ^[14]^ | **×** |  |  |  |  |  |  |  |  |  |
| HIV test | **×** |  |  |  |  |  |  |  |  |  |
| Pregnancy test ^[15]^ |  | **×** | when necessary | | | | | |  |  |
| Myocardial enzyme ^[16]^ |  | **×** | **×** | **×** | **×** | **×** | **×** | **×** | **×** |  |
| Adverse event ^[17]^ | **×** | **×** | **×** | **×** | **×** | **×** | **×** | **×** | **×** |  |
| Vital signs ^[18]^ |  | **×** | **×** | **×** | **×** | **×** | **×** | **×** | **×** |  |
| Physical examination ^[19]^ |  | **×** | **×** | **×** | **×** | **×** | **×** | **×** | **×** |  |
| Height and weight ^[20]^ |  | **×** | **×** | **×** | **×** | **×** | **×** | **×** | **×** |  |
| ECOG performance status score |  | **×** | **×** | **×** | **×** | **×** | **×** | **×** | **×** |  |
| 12-lead electrocardiogram ^[21]^ |  | **×** | when necessary | | | | | | |  |
| Echocardiography ^[22]^ |  | **×** | when necessary | | | | | | |  |
| Blood pressure monitoring ^[23]^ |  | **×** | **×** | **×** | **×** | **×** | **×** | **×** | **×** |  |

| **Project/assessment time** | **Screening period** | | **Treatment period**  **(21 days for 1 treatment cycle)** | | | | | **Treatment**  **End** | **Follow-up period** | |
| --- | --- | --- | --- | --- | --- | --- | --- | --- | --- | --- |
|  | **-21d** | **-7d** | **Cycle 1^[1]^** | **Cycle 2** | **Cycle 3** | **Cycle 4** | **Cycle n** |  | **Safety follow-up** | **Tumor progression and survival follow-up** |
|  |  |  | **Day 1** | **Day 1** | **Day 1** | **Day 1** | **Day 1** |  |  |  |
|  |  |  |  | (±3d) | (±3d) | (±3d) |  | (±7d) |  |  |
| Trial drugs | | | | | | | | | | |
| Administration of apatinib ^[24]^ |  |  | **×** | **×** | **×** | **×** | **×** |  |  |  |
| Administration of camrelizumab ^[25]^ |  |  | **×** | **×** | **×** | **×** | **×** |  |  |  |
| Administration of oxaliplatin ^[26]^ |  |  | **×** | **×** | **×** | **×** |  |  |  |  |
| Administration of S-1^[27]^ |  |  | **×** | **×** | **×** | **×** |  |  |  |  |
| Apatinib distribution/return^[28]^ |  |  | **×** | **×** | **×** | **×** | **×** | **×** |  |  |
| S-1 distribution/return ^[29]^ |  |  | **×** | **×** | **×** | **×** |  | **×** |  |  |
| Efficacy evaluation | | | | | | | | | | |
| Radiologic evaluation^[30]^ | **×** |  |  |  | **×** |  | **Every 6-8 weeks** | **×** |  |  |
| Biomarker^[31]^ |  | **×** | **×**  **×**  **×** | | | | | **Progressive disease** |  |  |
| Follow-up after the end of treatment | | | | | | | | | | |
| Time to disease progression |  |  |  |  |  |  |  |  |  | **×** |
| Time of death^[32]^ |  |  |  |  |  |  |  |  |  | **×** |
| Subsequent antitumor therapy ^[32]^ |  |  |  |  |  |  |  |  |  | **×** |

[1] If the follow-up examination planned before the first medication has already been conducted during the screening period and within 7 days before the first medication, a repeat examination is not necessary before the medication in the first cycle.

[2] Tumor history: tumor diagnosis, surgical history, treatment history, and radiotherapy history are included. Tumor diagnosis includes histological classification, histological grade, Lauren classification, clinical staging, and the time of initial diagnosis.

[3] Other medical histories: drug allergy history, diagnosis and treatment history of other diseases, and history of tumors other than gastric carcinoma (GC) and gastroesophageal junction carcinoma (GEJC).

[4] Medication history: Medication history within 4 weeks before signing informed consent.

[5] Combined medication and concomitant therapy: Information on combined medication and concomitant therapy from the signing of the informed consent form to the end of the safety follow-up period is recorded. Once the subjects discontinued the trial treatment, only the combined medications and concomitant treatments used for the new or unresolved adverse events related to the trial treatment are recorded.

[6] Complete blood count: white blood cell (WBC), neutrophil (NEU), lymphocyte (LYM), red blood cell (RBC), hemoglobin (Hb), and platelet (PLT) counts within 7 days before the first medication, before medication on the 1st day of each treatment cycle, and at the end of the trial.

[7] Blood biochemistry: total bilirubin (TBIL), direct bilirubin (DBIL), glutamate-alanine aminotransferase (ALT), aspartate aminotransferase (AST), alkaline phosphatase (AKP), γ-glutamyltransferase (γ-GT), total protein, albumin, urea/blood urea nitrogen (BUN), creatinine (Cr)/endogenous creatinine clearance rate, uric acid, fasting blood glucose, triglycerides, cholesterol, potassium, sodium, chlorine, calcium, and phosphorus within 7 days before the first medication, before medication on the 1st day of each treatment cycle and at the end of the study treatment.

[8] AFP: Alpha-fetoprotein (AFP) is measured within 7 days before the first medication, at each efficacy evaluation, and at the end of the trial.

[9] Serum amylase test: This test should only be performed within 7 days before the first medication. For subjects receiving camrelizumab treatment, the test should also be performed before medication on the 1st day of each treatment cycle and at the end of the trial. Serum lipase tests should be performed when serum amylase is abnormal and has clinical significance.

[10] Urinalysis: urine protein, urine glucose, urine occult blood, urine red blood cell, and white blood cells. If 2 consecutive semiquantitative methods showed protein ≥ 2+, a quantitative test of 24-hour urine protein is performed; the test is performed within 7 days before the first medication, before the medication on the 1st day of each treatment cycle, and at the end of the trial.

[11] Fecal occult blood: If the fecal occult blood test is positive, it is retested. If fecal occult blood is positive the second time, based on the clinical judgment, gastroscopy is performed if necessary; this can be performed within 7 days before the first medication, and during treatment at the discretion of the investigator.

[12] Coagulation function tests: including the international normalized ratio (INR), activated partial thromboplastin time (APTT), prothrombin time (PT), and fibrinogen (FIB). The tests are performed within 7 days before the first medication, before the medication on the 1st day of each treatment cycle, and at the end of the trial.

[13] Thyroid function tests: including serum-free triiodothyronine (FT3), free thyroxine (FT4), and serum thyroid-stimulating hormone (TSH), which are only performed within 21 days before the first medication. For subjects receiving camrelizumab, the tests should also be performed on the 1st day of each treatment cycle (except C1D1) and at the end of the trial treatment.

[14] Five liver function tests: if the result of hepatitis B surface antigen (HBsAg) is abnormal, the quantitative detection of HBV DNA viral load testing should be performed. If the anti-HCV antibody and HCV antibody results are positive, the HCV RNA viral load testing is detected within 21 days before the first medication.

[15] Female subjects who can become pregnant underwent blood pregnancy tests within 72 h before the first medication. If necessary, other tests are performed to rule out pregnancy. The investigators conducted the tests during the test and at the end of the study when necessary based on the conditions of the subjects.

[16] Myocardial enzyme tests: including creatine kinase MB isoenzyme (CK-MB), cardiac troponin I (cTnI), and lactate dehydrogenase (LDH); only one examination is performed within 7 days before the first medication. For subjects treated with camrelizumab, this test should also be performed on the 1st day of each treatment cycle, before the medication, and at the end of the trial.

[17] Adverse events: Adverse events are recorded from the signing of informed consent to the end of the safety follow-up period and are followed up until the end of the safety follow-up period or remission, relieved to the baseline level or ≤ Grade 1, reached a stable state, or reasonably explained (such as loss to follow-up or death). The safety follow-up period is 90 days after the last use of camrelizumab or 30 days after the last use of apatinib (whichever is longer).

[18] Vital signs：including temperature, pulse, respiratory rate, and blood pressure, which are measured within 7 days before the first medication, before the medication on the first day of each treatment cycle, and at the end of the trial.

[19] Physical examination: A comprehensive physical examination (general condition, head and face, skin, lymph node, eye, ear, nose and throat, oral cavity, respiratory system, cardiovascular system, abdomen, reproductive-urinary system, musculoskeletal system, nervous system, mental status, etc.) is performed within 7 days before the first medication and at the end of treatment/withdrawal from the trial. Before the medication on the first day of each treatment cycle, , a targeted physical examination is performed if clinically indicated.

[20] Height and weight: The measurements are performed within 7 days before the first medication, before medication on the 1st day of each treatment cycle, and at the end of the trial.

[21] 12-lead echocardiogram (ECG): this examination is performed within 7 days before the first medication, this examination should be supplemented if necessary according to the judgment of the investigator. If an abnormal ECG (with clinical significance) is found, two additional ECGs should be performed, and other tests, such as a 24-hour Holter ECG, should be performed to confirm the diagnosis.

[22] Echocardiography: LVEF (%) must be included. The examination should be performed within 7 days before the first medication. This examination should be supplemented when there are clinically significant ECG abnormalities during the trial.

[23] Blood pressure monitoring: The subjects’ blood pressure is measured during the screening period. Smoking and caffeine are prohibited within 30 minutes before each blood pressure measurement. Blood pressure is taken after the subjects had rested quietly for at least 10 minutes. The subjects are seated during the measurement, with the elbow placed at the level of the heart, and each blood pressure measurement is taken on the ipsilateral side. During the clinical trial period, blood pressure monitoring is completed by the subjects themselves, and the results are recorded on the subjects’ diary cards. During the first two periods, blood pressure is measured at least three times a week. If the blood pressure is abnormal, the blood pressure is followed up every day; if the blood pressure is normal, the blood pressure is tested at least twice a week after the second cycle. In addition, blood pressure is measured at each follow-up.

According to the *2018 Chinese Guidelines for Prevention and Treatment of Hypertension*, it is recommended that blood pressure be measured at least twice, with an interval of 1 to 2 minutes. If the difference is ≤ 5 mmHg, the average value of the two measurements is taken; if the difference is >5 mmHg, the measurement is performed again. Then the average value of the three measurements is taken.

[24] Apatinib (250 mg, orally, Qd) is administered at 0.5 h after a meal, and the time of daily medication is as consistent as possible. Twenty-one days is regarded as one cycle. Apatinib is given for 24 months or until disease progression, intolerable toxicity, or other causes warranting stoppage arise.

[25] Camrelizumab (200 mg, intravenous drip) is administered for more than 30 minutes (no less than 20 minutes and no more than 60 minutes, including the washout period) once every 3 weeks. One cycle lasted 21 days, and the interval between the two doses is no less than 12 days. Treatment is administered for 24 months or until disease progression, intolerable toxicity, or other reasons warranting stoppage arise.

[26] Oxaliplatin (130 mg/m^2^) is administered via intravenous infusion between 2 and 4 hours on Day 1, with 21 days as one cycle. Intervention group 1 is treated for a maximum of four cycles or until disease progression or intolerable toxicity.

[27] S-1: the initial dose of S-1 is determined based on body surface area (BSA< 1.25 m^2^, 40mg bid；1.25 m^2^ ≤ BSA ≤ 1.5 m^2^, 50 mg bid; BSA>1.5 m^2^, 60 mg bid), and is administered orally twice a day, after breakfast and dinner (d1-14), with 21 days as one cycle. S-1 is given for a maximum of four cycles or until disease progression, intolerable toxicity, or other reasons warranting stoppage arise.

[28] Apatinib distribution/return: Apatinib is distributed on the 1st day of the first cycle. Starting from the 1st day of the second cycle, the distribution and return of apatinib are performed on the first day of each cycle. First, the leftover drugs are returned. After verifying the actual dosage of the drugs, a new trial drug is issued. The dosage should be determined according to the dose adjustment principle before drug dispensing for each cycle.

[29] S-1 distribution/return: For patients in intervention group 1 who have not previously received late systemic chemotherapy, S-1 is distributed on the 1st day of the first cycle, and from the 2nd to the 4th cycle, the distribution and return of S-1 are performed on the first day of each cycle. First, the remaining drugs are returned. After verifying the actual dosage of the drugs, a new trial drugs is issued. The dosage should be determined according to the dose adjustment principle before drug dispensing for each cycle.

[30] Imaging evaluation: All lesions are recorded and evaluated according to the Response Evaluation Criteria in Solid Tumors (RECIST) 1.1. Imaging examinations include chest CT and enhanced CT scans of the abdomen and pelvis (CT scan thickness ≤ 5 mm). If the patient is allergic to the contrast agent for enhanced CT, the patient can undergo a plain CT scan of the chest + MRI scan of the abdomen and pelvis. If brain metastases are suspected, contrast-enhanced MRI or CT scan of the brain is required to rule them out. Bone scan is required when definite or clinically suspected bone metastasis occur. The allowed window for imaging examinations is ±7 days unless otherwise stated. Unplanned imaging examinations may be done when disease progression (such as worsening symptoms) is suspected.

- During the screening period, the baseline tumor assessment can be relaxed to no more than 3 weeks before treatment, and the CT/MRI scan results obtained before signing informed consent can be used in the tumor assessment during the screening period, as long as they met the requirements;
- During the treatment period, imaging examinations should be performed under the same conditions as the baseline examination (layer thickness of the scan, use of contrast agent, etc.) every 2 cycles during the treatment period and at the end of treatment;
- If treatment is discontinued during the treatment period due to reasons other than progression of imaging studies, imaging examination should be performed at the end of trial treatment, unless imaging examination is performed within 28 days;
- For the subjects who still did not have radiological progression at the end of trial, the follow-up frequency during the treatment period should be maintained during the tumor progression and survival follow-up period, and imaging assessments should be performed every 6-8 weeks until the radiological progression, the receipt of other antitumor treatments, the withdrawal of informed consent, the loss to follow-up, or the death of the patients.

[31] Specimen collection: Blood samples are collected from the subjects during the screening period (-7 d to -1 d), each imaging evaluation during the treatment period, and at the time of disease progression, with 2 mL of blood (with anticoagulation) and 5 mL (without anticoagulation) collected each time. The AFP level in the blood is detected, and the efficacy of the predictive markers is explored by next-generation sequencing (NGS). In addition, 10 paraffin-embedded pathological tissue sections are collected from the subjects before the first medication and at the time of disease progression, whenever possible, to measure the expression levels of HER2, EGFR, VEGFR2, and PD-L1, as well as blood vessel density and other indicators.

[32] After the termination of trial treatment, the survival status and subsequent antitumor treatment information will be collected through clinical follow-up or telephone follow-up every 3 months until the death of the subject, loss to follow-up, termination of the study by the sponsor, or other end-of-study criteria are met (whichever occurs first).

# Protocol summary

| **Title** | A prospective nonrandomized multicenter phase II clinical trial of camrelizumab combined with apatinib for unresectable/recurrent or metastatic alpha-fetoprotein-producing gastric/gastroesophageal junction adenocarcinoma |
| --- | --- |
| **Protocol ID** | MA-GC-II-007 |
| **Version number** | 2.0 |
| **Sponsor** | Peking University Cancer Hospital |
| **Principal investigator** | Professor Xiaotian Zhang |
| **Nature of research** | Investigator-initiated clinical trial |
| **Research subjects** | Unresectable, recurrent, or metastatic gastric/gastroesophageal junction adenocarcinoma (AFPGC) with alpha-fetoprotein (AFP) greater than 2 times the upper limit of the normal or positive AFP by immunohistochemistry |
| **Research objectives** | **Primary objectives**   - To evaluate the objective response rate (ORR) of patients receiving camrelizumab combined with apatinib and SOX as the first-line treatment of AFPGC. - To evaluate the ORR of patients receiving camrelizumab combined with apatinib as the second-line and above treatment of AFPGC.   **Secondary objectives**   - To evaluate the progression-free survival (PFS), disease control rate (DCR), duration of remission (DoR), time to response (TTR), overall survival (OS), and safety of patients receiving camrelizumab combined with apatinib and SOX as the first-line treatment of AFPGC. - To evaluate the PFS, DCR, DoR, TTR, OS, and safety of patients receiving camrelizumab combined with apatinib as the second-line and above treatment of AFPGC.   **Exploratory objectives**   - To explore the correlation of changes in biomarkers such as human epidermal growth factor receptor-2 (HER2), epidermal growth factor receptor (EGFR), vascular epidermal growth factor receptor 2 (VEGFR2), vessel density, programmed death ligand 1 (PD-L1), and radiomic features with treatment efficacy. - To explore the correlation between the increase in AFP from baseline or the decreasing level of AFP and the efficacy endpoints such as ORR, PFS, and OS. |
| **Study endpoints** | **Primary endpoints**   - ORR evaluated by the investigator based on RECIST 1.1.   ORR refers to the percentage of subjects with complete response (CR) or partial response (PR). A first assessment of CR or PR should be confirmed by a subsequent tumor assessment at least 4 weeks later.  **Secondary endpoints**  Efficacy:   - ORR evaluated by the investigator based on iRECIST. - DCR, DoR, TTR, and PFS evaluated based on RECIST 1.1.   DCR: proportion of subjects with confirmed CR, PR, or stable disease.  DoR: the time from the date of the first confirmed CR/PR to the date of the first disease progression or death due to any cause.  TTR was defined as the time from treatment initiation to the first confirmed CR/PR.  PFS: the time from the date of treatment initiation to the date of the first disease progression or death due to any cause, whichever occurs earlier.   - DCR, DoR, and PFS evaluated based on iRECIST. - OS: the time from the date of treatment initiation to the date of death due to any cause.   Safety:   - Adverse events (AE), including type, incidence, toxicity grading (based on NCI-CTCAE v5.0 standard), duration, and correlation with the study drugs. - Abnormal values of laboratory indicators, type, incidence, grading (based on NCI-CTCAE v5.0 criteria), and duration. - Vital signs, including blood pressure, pulse, and respiratory rate, electrocardiography, and Eastern Cooperative Oncology Group (ECOG) performance status (PS).   Tolerability:   - Proportion of dose interruption/delay, dose reduction and termination during the trial due to trial drug-related toxicity.   **Exploratory endpoints:**   - Correlation of changes in biomarkers such as HER2, EGFR, VEGFR2, vessel density, PD-L1 and radiomics with efficacy. - Correlation of increase from baseline value and decrease proportion of AFP with efficacy endpoints such as ORR, PFS, and OS. |
| **Study design** | This study is a prospective, open-label, multicenter nonrandomized phase II clinical trial.  This study is divided into two intervention groups. Intervention group 1 includes patients with unresectable gastric/gastroesophageal junction adenocarcinoma who have not received advanced systemic treatment before and are given camrelizumab combined with apatinib and SOX (S-1 capsule in combination with oxaliplatin) as the first-line treatment. Intervention group 2 includes patients with gastric/gastroesophageal junction adenocarcinoma who fail first-line and above treatment and are given second-line and above treatment consisting of camrelizumab combined with apatinib. The efficacy and safety of both regimens will be observed. The study will also monitor the dynamic changes in AFP in serum and/or tissues, the changes in biomarkers such as HER2, EGFR, VEGFR2, and radiomics to explore the predictive value of changes in AFP for treatment efficacy and the correlations between biomarkers and efficacy. The results will provide guidance for the clinical management of this special type of GC and evidence for a phase III randomized controlled clinical trial. |
| **Treatment** | **Intervention group 1 (first-line treatment of AFPGC patients with camrelizumab combined with apatinib and SOX):**  Camrelizumab: 200 mg, IV, d1; 21 days as one cycle.  Apatinib: 250 mg orally within 0.5 h after meal, qd; 21 days as one cycle.  S-1 capsule: the initial dose of tigeol capsule is determined by body surface area (BSA <1.25 m^2^, 40 mg bid; 1.25 m^2^ ≤ BSA ≤ 1.5 m^2^, 50 mg bid; BSA > 1.5 m^2^, 60 mg bid), and is administered orally twice daily, after breakfast and dinner, d1-14, for 21 days.  Oxaliplatin: 130 mg/m^2^, 2-4-hour IV infusion, d1; 21 days as one cycle.  While receiving camrelizumab in combination with chemotherapy, the subjects are given intravenous infusion of camrelizumab on the first day of each cycle, followed by an interval of at least 30 minutes, and then intravenous infusion of oxaliplatin.  If there is no definite disease progression after four cycles, the patient will start maintenance therapy with camrelizumab combined with apatinib at the same dose as before and will be treated until disease progression or other events meeting the end-of-treatment criteria.  **Intervention group 2 (AFPGC patients treated with camrelizumab combined with apatinib as second-line or above):**  Camrelizumab: 200 mg, IV, d1; 21 days as one cycle.  Apatinib: 250 mg orally within 0.5h after meal, Qd, 21 days as one cycle.  Patients are treated until disease progression or other events that met the end-treatment criteria. |
| **Inclusion criteria** | Patients must meet all the following inclusion criteria to be included in this study:   1. Patients voluntarily joined the study and signed informed consent; 2. Male or female, age ≥18 years; 3. ECOG PS 0-2; 4. Pathologically confirmed gastric/gastroesophageal junction adenocarcinoma; 5. Clinical staging is performed based on enhanced CT/MRI examination (combined with ultrasound gastroscopy and diagnostic laparoscopy when necessary). Patients with unresectable locally advanced or advanced disease with stage III-IV (AJCC 8th edition of TNM staging of gastric cancer), and the possibility of radical surgery is discussed and determined by the MDT; 6. Patients who had not received advanced systemic treatment (intervention group 1); patients who had received at least first-line systemic treatment (intervention group 2);   Note: Neoadjuvant therapy is not included in the number of treatment lines; for patients with relapse within 6 months after the end of adjuvant therapy, the adjuvant therapy part is defined as first-line treatment; for patients with relapse after more than 6 months after the end of adjuvant therapy, the adjuvant therapy part is not included in number of treatment lines.   1. Extragastric measurable lesions (RECIST 1.1 criteria); 2. AFP >2 times the upper limit of the normal value or positive AFP in immunohistochemistry; 3. The baseline complete blood count and biochemical indicators of the subjects meet the following criteria (without blood transfusion or blood products within 14 days before the first medication, and the patients are not corrected by G-CSF and other hematopoietic stimulatory factors):   hemoglobin ≥90 g/L;  absolute neutrophil count（ANC）≥1.5×10^9^/L;  platelet≥80×10^9^/L;  ALT and AST ≤2.5 times the normal upper limit; if patients are accompanied by liver metastasis, ALT and AST ≤5 times the normal upper limit;  serum total bilirubin ≤1.5 times the upper limit of normal;  serum Cr ≤ 1.5 times the upper limit of normal or endogenous creatinine clearance > 50 ml/min (male: endogenous creatinine clearance = ((140-age) × body weight)/(72×serum Cr); female: endogenous creatinine clearance = ((140-age) × body weight)/(72×serum Cr) × 0.85; body weight in kg; serum Cr in mg/mL);  serum albumin ≥30 g/L;   1. No serious concomitant disease that makes the survival <5 years; 2. Ability to swallow medication normally; 3. Good compliance and adherence to the regimen during the study; 4. Female subjects who can get pregnant need to have a negative serum pregnancy test result within 72 h before the first medication and agree to use highly effective contraception during the trial and for 90 days after the end of trial. Male subjects whose partner is a woman of childbearing potential should agree to use highly effective contraception during the trial and for 90 days after the end of trial; 5. Blood and/or tissue specimens. |
| **Exclusion criteria** | Patients with any of the following can not be included in this study:   1. Pregnant or breastfeeding women; 2. Known HER2 positivity (intervention group 1; HER2 positivity defined as IHC 3+ or IHC2+/ ISH+); 3. Prior treatment-induced adverse events (other than alopecia) that did not recover to ≤ grade 1 (CTCAE v5.0); 4. History of other malignant diseases in the past 5 years or at the same time, other than cured basal cell carcinoma of the skin and carcinoma in situ of the cervix; 5. For patients with a history of uncontrolled epilepsy, central nervous system diseases, or mental disorders, the investigators should determine whether the clinical severity prevents the signing of informed consent or affects the patient’s compliance with oral medications; 6. Not well-controlled, clinically serious (i.e., active) heart disease such as (1) symptomatic coronary artery disease; (2) New York Heart Association class II or worse congestive heart failure or severe arrhythmia requiring pharmacologic intervention; (3) myocardial infarction within the last 12 months; (4) QTc interval ≥450ms in male and ≥470ms in female; or (5) LVEF <50%; 7. Arterial/venous thrombosis events, such as cerebrovascular accidents (including transient ischemic attack, cerebral hemorrhage, and cerebral infarction), deep venous thrombosis and pulmonary embolism, that occurred within the 6 months before enrollment; 8. For patients who have clinically significant bleeding symptoms or have a clear bleeding tendency within the previous 3 months, such as gastrointestinal bleeding, hemorrhagic gastric ulcer, etc., if the fecal occult blood is positive during the screening period, re-examination can be performed; if the re-examination is still positive, gastroscopy is performed based on clinical judgment (except for patients who had received gastroscopy within 3 months before enrollment, and this condition is excluded). 9. Known hereditary or acquired bleeding and thrombocytopenia (such as hemophilia, coagulation disorders, thrombocytopenia, etc.); 10. Upper gastrointestinal obstruction or abnormal physiological function or malabsorption syndrome, which may affect the absorption of oral drugs; 11. Previous gastrointestinal perforation, abdominal abscess, or intestinal obstruction in the past 3 months, or imaging findings and clinical symptoms suggesting intestinal obstruction; 12. Abnormal coagulation function (INR>2.0 or prothrombin time >16 s), a bleeding tendency, or current thrombolytic or anticoagulant therapy (the preventive use of small doses of aspirin, low-molecular-weight heparin, etc., is permitted); 13. Patients with neurotoxicity caused by chemotherapy, who are determined by the investigators to be not suitable for the use of oxaliplatin, can not be included in intervention group 1; but patients who only exhibit disappearance of the deep tendon reflex do not need to be excluded; 14. Need for immunosuppressive therapy for organ transplantation, or use of immunosuppressive drugs or systemic corticosteroids within 14 days before medication for the purpose of immunosuppression (such as >10 mg/day prednisone or other drug equivalent doses); 15. Active ulcers, unhealed wounds, or fractures; 16. Hypertension that is not well controlled by antihypertensive drug treatment (systolic blood pressure ≥140 mmHg or diastolic blood pressure ≥90 mmHg); 17. Known dihydropyrimidine dehydrogenase deficiency (intervention group 1 only); 18. Known allergies to any trial drugs or excipients; 19. Previous treatment with PD1/PD-L1 monoclonal antibody or apatinib; 20. Urinalysis that suggests urinary protein ≥ ++ and confirmed 24-hour urine protein volume >1.0 g; 21. Serosal effusion with clinical symptoms (including ascites, pleural effusion, and pericardial effusion) that required symptomatic treatment. Asymptomatic patients with serous effusion can enroll; symptomatic serous effusion will be treated after drainage and other active treatments. For patients with good control, the investigators will determine if the enrollment is allowed; 22. Active hepatitis (hepatitis B reference: HBsAg positive and HBV DNA ≥ 500 IU/ml; hepatitis C reference: anti-HCV positive and HCV viral copy number > upper limit of the normal value); or active infection stage that requires antimicrobial treatment (e.g., antibacterial drug therapy, antifungal drug therapy); 23. Current interstitial pneumonia or interstitial lung disease; other diseases such as active tuberculosis, pulmonary fibrosis, organizing pneumonia, pneumoconiosis, drug-associated pneumonia, idiopathic pneumonia, or active pneumonia; or severe lung function impairment as seen on CT during the screening period; 24. Active autoimmune diseases or patients with a history of autoimmune diseases and possible relapse [including but not limited to immune hepatitis, interstitial pneumonia, uveitis, enteritis, hypophysitis, vasculitis, nephritis, hyperthyroidism, hypothyroidism (subjects controllable by hormone replacement therapy can be included)]. Skin diseases that do not require systemic treatment (such as vitiligo, psoriasis, alopecia), type I diabetes that is controlled by insulin therapy, and childhood asthma that is in complete remission without intervention are allowed. Asthma requiring bronchodilator intervention is not allowed. 25. As determined by the investigators, other factors that may affect the trial results or cause the trial to be stopped halfway, such as alcohol abuse, drug abuse, and other serious diseases (including mental illness) that require combined treatment, as well as abnormal laboratory results and family or social factors that can affect the safety of the patient, are exclusion criteria. |
| **Criteria for termination of treatment** | Termination of study treatment does not imply withdrawal from the trial. Subjects who stop the trial treatment must come to the remaining visits as required by the protocol. If the subject met any of the following criteria, the trial treatment must be terminated:   1. The subject voluntarily withdraws from treatment, stops the trial medication, or withdraws informed consent; 2. The disease progresses based on iRECIST assessment; 3. The patient cannot tolerate a drug toxicity; 4. Significant protocol deviations such as the ineligibility of the subjects are found after enrollment (the decision will be discussed with the sponsor); 5. The subject does not comply with a protocol; 6. The patient is lost to follow-up; 7. The investigator perceives other circumstances where it is necessary to stops the study medication; 8. The subject gets pregnant. |
| **Criteria for withdrawal from the study** | The reasons for a subject’s withdrawal from the study may include:   1. The subject withdraws informed consent for participation and refuses further follow-up; 2. The investigator believes that it is necessary to withdraw a subject from the trial; for example, the subject loses the ability to freely express his will due to imprisonment or isolation; 3. The subject is lost to follow-up; 4. The subject dies; 5. The trial itself is terminated; 6. The subject gets pregnant. |
| **Study termination criteria** | The termination criteria for the trial include but are not limited to the following:  1.Unexpected, significant, or unacceptable risks to patients are found;  2. A major error in the protocol is discovered during the trial;  3. The study drug/trial treatment is not effective, or it is pointless to continue the trial;  4. Due to reasons such as severe lag in patient enrollment or frequent protocol deviations, the study is terminated. |
| **Safety evaluation** | The severity of adverse events is determined according to the CTCAE v5.0 standard. During the trial, the adverse event record form will be truthfully filled in, including the time of occurrence, severity, correlation with the study drugs, duration, measures adopted, and outcomes of the adverse events. |
| **Evaluation of efficacy** | According to RECIST 1.1, the investigators perform tumor imaging evaluation every 6-8 weeks (±7 d) until radiological disease progression, administration of other antitumor treatments, withdrawal of informed consent, loss to follow-up, or death of the patient. If treatment is discontinued during the treatment period for reasons other than radiological progression, imaging evaluation should be performed at the end of trial treatment (unless imaging evaluation has been performed within 28 days) and every 6-8 weeks during the follow-up period for tumor progression and survival until the patient has radiological disease progression, starts other antitumor treatment, withdraws informed consent, lost to follow-up, or dies. Unplanned imaging examinations may be performed when disease progression is suspected (such as symptoms worsening). |
| **Sample size** | This is a prospective, open label, multicenter, phase II nonrandomiz- controlled clinical trial with two prospective intervention groups. The primary endpoint of this study is the ORR, and the Simon two-stage optimal design (Simon, 1989) is used.  Intervention group 1: A previous retrospective analysis of 105 patients showed an ORR of 30% in first-line chemotherapy for AFP-G/GEJ cancer patients with AFP concentration ≥ 160 ng/ml. The null hypothesis H_0_ is that ORR = 30%. The alternative hypothesis H_1_ is that ORR = 55%. The one-sided type-I-error is 5% and statistical power is 80%. Nine subjects will be enrolled in the first stage. Among these 9 subjects, if at least four patients achieve a confirmed objective response (CR or PR per RECIST 1.1), the study will proceed to the second stage and continue to enroll another 26 subjects. Among all 35 subjects, if at least 15 patients achieve confirmed CR or PR in total, the null hypothesis will be rejected.  Intervention group 2: The standard second-line treatment for GC is paclitaxel/docetaxel/irinotecan monotherapy. Previous analysis showed an ORR of 15-25% approximately. Since AFP elevation is an unfavorable prognostic factor in GC, null hypothesis H_0_ is set as ORR= 10%, and alternative hypothesis H_1_ is ORR = 30%. The one-sided type-I-error is 5% and the statistical power is 80%. Ten patients will be enrolled in the first stage. If among these 10 patients at least two achieve confirmed CR or PR per RECIST 1.1, the study will proceed to the second stage and continue to enroll another 19 subjects. Among all 29 subjects, if at least 6 patients achieve CR or PR, the null hypothesis will be rejected.  A total of 64 patients are expected to be enrolled in the two groups, with 35 subjects in intervention group 1 and 29 subjects in intervention group 2. A total of 19 subjects will be enrolled in the two intervention groups in the first stage, and 45 subjects will be enrolled in the two intervention groups in the second stage. |
| **Data statistical analysis** | The efficacy analysis will be performed mainly in FAS for each intervention group. For ORR and DCR, the 95% confidence interval will be calculated using Clopper-Pearson method based on the binomial distribution. The Kaplan‒Meier method will be used for the analyses of PFS, DoR, TTR and OS, the median time to event and the corresponding 95% confidence intervals are to be calculated based on , and the Kaplan‒Meier curves will also be presented. The efficacy analysis of the subgroups is performed based on age, sex, primary tumor location, metastatic locations, and AFP levels of the subjects.  All AEs in this study are coded according to the latest version of MedDRA, and frequency distribution, charts, or other descriptive indicators are analyzed in accordance with the National Cancer Institute Common Terminology Criteria for Adverse Events (NCI-CTCAE) version 5.0. The number and incidence of all AEs, AEs related to the study treatment, and serious AEs (SAEs) will be calculated, and the severity of the AEs and their relationship with the trial treatment will further be summarized in descriptive statistics. |
| **Trial time** | First subject enrollment: 2020.9  Last subject enrollment: 2023.03  Trial end: 2024.03 |

# Abbreviations

| **Abbreviation** | **Full English name** |
| --- | --- |
| AEs | Adverse event |
| AFP | Alpha-fetoprotein |
| AFPGC | AFP-producing gastric cancer |
| AKP | Alkaline phosphatase |
| ALT | Alanine aminotransferase |
| APTT | Activated partial thromboplastin time |
| AST | Aspartate aminotransferase |
| BUN | Blood urea nitrogen |
| CFR | Code of Federal Regulations |
| CR | Complete response |
| Cr | Creatinine |
| CT | Computed tomography |
| DCR | Disease control rate |
| DOR | Duration of overall response |
| eCRF | Electronic case report form |
| EDC | Electronic data capture |
| EGFR | Epidermal growth factor receptor |
| FAS | Full analysis set |
| FIB | Fibrinogen |
| GCP | Good Clinical Practice |
| G-CSF | Granulocyte colony-stimulating factor |
| HBsAg | Hepatitis B surface antigen |
| HBV | Hepatitis B virus |
| HCG | Human chorionic gonadotropin |
| HCV | Hepatitis C virus |
| HER2 | Human epidermal growth factor receptor-2 |
| HIV | Human immunodeficiency virus |
| ICH | International Council for Harmonization |
| ICI | Immune checkpoint inhibitors |
| irAE | Immune-related adverse events |
| INR | International normalized ratio |
| IU | International unit |
| LDH | Lactate dehydrogenase |
| LEU | Leukocytes in urine |
| LVEF | Left ventricular ejection fraction |
| MRI | Magnetic Resonance Imaging |
| NCI-CTCAE v5.0 | National Cancer Institute - Common Terminology Criteria for Adverse Events Version 5.0 |
| NYHA | New York Heart Association |
| ORR | Objective response rate |
| OS | Overall survival |
| PFS | Progression-free survival |
| PPS | Per-Protocol Set |
| PT | Prothrombin time |
| RECIST v1.1 | Response Evaluation Criteria in Solid Tumors |
| iRECIST | Immune-related Response Evaluation  Criteria in Solid Tumors |
| SAE | Serious adverse event |
| SS | Safety set |
| TBIL | Total bilirubin |
| TTR | Time to response |
| ULN | Upper Limit of Normal |
| γ-GT | γ-glutamyl transpeptidase |
| VEGFR2 | Vascular epidermal growth factor receptor 2 |

# Research Background and Scientific Evidence

## Research Background

### Overview of gastric cancer (GC)

GC is one of the most common malignant tumors of the digestive system. Originating from the mucosal epithelial cells of the most surface layer of the stomach wall, 95% of these tumors are adenocarcinomas. They can occur in all parts of the stomach and can invade different depths and widths of the stomach wall. Globally, the incidence of GC ranks fifth among all malignant tumors, and its mortality ranks third [1]. In 2014, there were 410,000 new cases of GC in China, with 294,000 deaths, making it the second most common malignant tumor in China and the third leading cause of death due to malignant tumors [2]. The pathogenesis of GC may be associated with a variety of factors, such as living habits, diet, environmental factors, genetic factors, and mental factors. It is also related to chronic gastritis, gastric polyps, dysplasia of the gastric mucosa and intestinal epithelial hyperplasia, postsurgical gastric remnants, as well as *Helicobacter pylori* infection.

### Alpha-fetoprotein-producing gastric cancer (AFPGC)

Clinically, AFPGC is characterized by the detection of AFP as its main feature and diagnostic standard, specifically, abnormally increased serum AFP or histologically abnormal expression of AFP. AFP is a glycoprotein mainly produced by the fetal liver and yolk sac and is often used as a marker of liver tumors or yolk sac tumors in clinical practice. The serum AFP level is increased in 1.3-15% of patients with GC, and these patients have distinctive clinicopathological features. As a result, the concept of serum AFP-elevated GC has gradually developed in clinical practice. After elevated serum AFP, AFP expression in tissues and hepatoid adenocarcinoma with features of hepatocellular carcinoma-like differentiation are thought to be the most common pathological types of AFP-producing GC. Although the definition of AFP production varies between different studies, AFPGC is generally more invasive, has a higher rate of liver metastasis, is mostly diagnosed in the advanced stage, and has a poorer prognosis than AFP-negative GC [3, 4].

Previous studies and clinical practice have shown that the response of AFPGC to traditional two-drug combination chemotherapy (platinum combined with fluorouracil) is poorer than that of AFP-negative GC, with an effective rate of only approximately 30%. Although triple-drug combination chemotherapy regimen can increase the effective rate to 60% in first-line treatment, serious adverse reactions can reduce the quality of life of patients and make later treatment very difficult [5]. Therefore, finding effective drugs has become a key step in improving the prognosis of AFPGC patients.

### Exploration of apatinib and programmed cell death protein 1 (PD-1) monoclonal antibody (mAb) in AFPGC

#### Data on apatinib in AFPGC

Apatinib, a small-molecule kinase inhibitor that targets vascular endothelial growth factor receptor 2 (VEGFR2), has become a promising drug with some gradually accumulated evidence in the treatment of AFPGC. Early basic research revealed that AFP-positive GC cell lines had active proliferation, reduced cell apoptosis, and abundant neovascularization compared to AFP-negative GC cell lines [6]. A few small observational studies all showed that the efficacy of apatinib in the treatment of advanced AFPGC patients who failed standard treatment was significantly higher than that in the overall GC population, with an objective response rate (ORR) of approximately 10% and a disease control rate (DCR) of 60-70% [7-10]. In addition, the efficacy of ramucirumab, an anti-VEGFR2 monoclonal antibody, in primary liver cancer was better in liver cancer patients with an AFP higher than 400 [11]. Therefore, high serum AFP may be a potential marker for the efficacy of apatinib in the treatment of GC. Prospective studies with large samples are still needed for further validation, and the specific mechanism of action remains to be further explored.

#### Exploration of a PD-1 mAb in AFPGC

Immunotherapy, represented by PD-1 mAb, is a type of drug that has been studied extensively in recent years. Blocking PD-1/programmed cell death ligand 1 (PD-L1) modulates the antitumor activity of T lymphocytes to improve the immune response to tumors, thereby killing the tumor. Currently, the subset of GC patients who benefit from PD-1/PD-L1 inhibitors are those receiving them as third-line treatment. The results of studies on second-line and first-line treatments have not surpassed those of traditional chemotherapy. Although PD-1 blockade can have long-term benefits for patients, the main problem with PD-1 mAb is the low effective rate of treating advanced GC, only 10-20%. At present, the effective population is mainly characterized by Epstein–Barr virus (EBV) positivity, high PD-L1 expression, and microsatellite instability-high (MSI-H) status [12]. Because AFP itself has the biological activity of immunosuppression, AFPGC has low immunogenicity and is weaker in the positive immune regulation ability of the immune microenvironment than AFP-negative GC [13, 14]. It is speculated that the efficacy of PD-1 mAb monotherapy treatment is poor. The combination of antiangiogenic drugs has become a major research direction for improving treatment efficacy. Antiangiogenic drugs can remodel the immune microenvironment of tumors by regulating immune cell function and normalizing blood vessels, which can turn “cold tumors” into “hot tumors” to some extent [15]. A number of exploratory phase I clinical trials of anti-PD-1/PD-L1 antibodies combined with antiangiogenic targeted drugs for the treatment of advanced GC/gastroesophageal junction cancer (GEJC) have been launched in China and abroad, and these trials have shown outstanding results. A phase I clinical trial of camrelizumab plus apatinib for the treatment of GC/GEJC saw an ORR of 17.4% and a DCR of 78.3% [16]. This combination has already entered the phase III clinical trial stage. A multicenter phase II clinical trial of camrelizumab combined with apatinib and the XELOX (oxaliplatin + capecitabine) regimen as the first-line treatment of advanced GC/GEJC recorded an ORR of 58.7% and a DCR of 78.3% [17]. These two trials were both aimed at the entire population of patients with advanced GC, so their results are not sufficient to guide clinical practice. Considering the heterogeneity of the immune microenvironment in GC, screening for reliable efficacy-predictive markers has become the key for accurate treatment of GC.

#### Potential mechanisms and preliminary studies of PD-1 mAb treatment in combination with chemotherapy or antiangiogenic drugs

There are two methods for improving the efficacy of the immune checkpoint inhibitors PD-1/PD-L1 mAb. We have explored molecular markers for the prediction of treatment efficacy. In our study, the expression level of PD-L1 in tumor tissues, the tumor mutation burden (TMB), and molecular markers such as MSI-H and dMMR were higher in patients with better PD-1/PD-L1 treatment efficacy. Second, suitable combination regimens for combination immune therapy should be identified. Many clinical trials of PD-1/PD-L1 inhibitors combined with other immune checkpoint inhibitors, traditional chemotherapeutic drugs, or targeted antitumor drugs have been performed. There is a growing body of evidence showing that the combination of PD-1/PD-L1 inhibitors and chemotherapeutic drugs or antiangiogenic drugs has a synergistic effect on treatment efficacy.

Regarding the combination of PD-1/PD-L1 inhibitors with chemotherapeutic drugs, basic research has shown that chemotherapeutic drugs may activate the immune system through various pathways [18] . Cytotoxic drugs cause tumor cell death and release large amounts of antigens, stimulating and activating the immune system. Certain chemotherapeutic drugs, such as cyclophosphamide, can inhibit Treg cells and weaken their immunosuppressive effect. Some chemotherapeutic drugs can inhibit myeloid suppressor cells and interferon-γ [18].

PD-1/PD-L1 inhibitors have been combined with antiangiogenic drugs. The VEGFR signaling pathway plays an important role in mediating tumor immune evasion, and inhibition of this pathway may enhance the tumor immune activation effect of PD-1 antibodies.

Preclinical data from Hengrui suggested that camrelizumab combined with apatinib could significantly reduce the level of regulatory T cells (T_reg_) in peripheral blood and increase the ratio of effector T cells (T_eff_) to T_reg_.

Animal studies have shown that camrelizumab combined with apatinib can significantly enhance the tumor growth-inhibitory effect of camrelizumab without increasing toxicity (no significant change in animal weight) (Figure 1).

Preclinical results suggest that the use of camrelizumab combined with apatinib may improve the ORR while maintaining the efficacy of immunotherapy.

Figure 1 The effect of camrelizumab combined with apatinib on MC38 colorectal cancer was evaluated in mice expressing human PD-1 Tg.

Given our growing understanding of AFPGC, the outstanding efficacy of apatinib in treating AFPGC, and its role in combination immunotherapy, we speculated that the combination of apatinib and PD-1 may further enhance the efficacy of AFPGC. Through this trial, we hope to verify the efficacy of apatinib combined with camrelizumab in AFPGC and to explore efficacy markers to guide clinical practice and provide direction for future studies.

### Risk/benefit assessment of this trial

Based on the efficacy of apatinib in treating AFPGC and the preliminary results of PD-1 combined with antiangiogenic drugs in patients with GC/GEJC, we will conduct a clinical trial on camrelizumab combined with apatinib as the basic treatment for AFPGC.

In terms of side effects, apatinib is an antiangiogenic tyrosine kinase inhibitors (TKIs), and its main toxic side effects include hypertension, proteinuria, and hand–foot skin reactions. From the safety results of phase II-III clinical trials of apatinib, the relatively common adverse events were hypertension, proteinuria, hand–foot skin reaction and fatigue, with incidence rates of 36.32%, 40.36%, 27.35%, and 17.94%, respectively. Camrelizumab is a PD-1 antibody, and its main toxic side effects include reactive cutaneous capillary endothelial proliferation (RCCEP), increased liver enzymes, and hypothyroidism. As shown by the safety data of 10 studies with a total of 1116 subjects, the common adverse events were RCCEP, high aspartate aminotransferase, high alanine aminotransferase, and hypothyroidism, with incidence rates of 77.4%, 19.0%, 17.5%, and 16.7%, respectively. In addition, immunotherapy may cause immune-related adverse reactions (incidence ≤ 1%), such as immune pneumonia, diarrhea, immune colitis, immune hepatitis, and immune nephritis. The safety data from previous studies suggest that two-drug combinations did not significantly increase the incidence of adverse events but reduced the incidence of RCCEP.

Currently, for AFPGC patients, the standard treatment regimen is the same as that for GC patients, and there is no special treatment option. In our intervention group 1, camrelizumab combined with apatinib and the standard regimen of oxaliplatin combined with S-1, recommended by the guidelines, will be administered to ensure that the subjects get the benefit of standard treatment. Intervention group 2 will be given the guideline-recommended monotherapy with paclitaxel, docetaxel, or irinotecan. The results of the Keynote-061 study suggested that PD-1 mAb was not inferior to paclitaxel monotherapy, so camrelizumab and apatinib treatment was used to ensure the benefit of the patients.

We expect that the treatment options of this trial will improve the treatment efficacy in advanced AFPGC patients with controllable safety. We hope that the results of this trial will provide exploratory and clinical guidance for identifying effective treatment options for AFPGC, a special GC population, and improving patient prognosis.

# Research objectives and study endpoints

**Primary objectives**

- To evaluate the ORR of patients treated with camrelizumab combined with apatinib and SOX as a first-line treatment for AFPGC/gastroesophageal junction adenocarcinoma.
- To evaluate the ORR of patients treated with camrelizumab combined with apatinib as a second-line treatment or above for AFP-producing gastric/gastroesophageal junction adenocarcinoma.

**Secondary objectives**

- To evaluate the progression-free survival (PFS), disease control rate (DCR), duration of response (DoR), overall survival (OS) and safety of patients receiving camrelizumab combined with apatinib and SOX for the first-line treatment of AFPGC.
- To evaluate the PFS, DCR, DoR, OS, and safety of camrelizumab combined with apatinib as the second-line and above treatment of AFPGC.

**Exploratory research objectives**

- To explore the changes in biomarkers such as HER2, EGFR, VEGFR2, vessel density, and PD-L1 as well as radiomic parameters and efficacy.
- To explore the correlation between the increase of AFP from the baseline and the decreasing proportion of AFP and the efficacy endpoints such as ORR, PFS, and OS.

**Primary endpoints**

- The ORR is evaluated by the investigator based on RECIST 1.1

ORR refers to the percentage of subjects with complete response (CR) or partial response (PR). A first finding of CR/PR should be confirmed by a subsequent tumor assessment at least 4 weeks later.

**Secondary endpoints**

Efficacy:

- The ORR is evaluated by the investigator based on the iRECIST criteria.
- PFS is evaluated by the investigator based on the RECIST 1.1 standard.

PFS is defined as the time from the date of treatment initiation to the date of the first occurrenc of disease progression or death due to any cause, whichever occurs earlier, according to the RECIST version 1.1 criteria. Subjects still alive without radiological progressive disease at data cut-off date will be censored at the last adequate tumor assessment date..

- PFS is evaluated by the investigators based on the iRECIST criteria;
- The DCR, TTR and DoR are evaluated by the investigator based on the RECIST 1.1 standard;

The DCR refers to the proportion of subjects with confirmed CR, PR, or stable disease.

TTR was defined as the time from treatment initiation to the first confirmed CR/PR.

DoR is defined as the time from the date of the first documentation of confirmed CR or PR (whichever occurs earlier) to the date of the first occurrence of progressive disease or death due to any cause, whichever occurs first. DoR is evaluated by the investigator. Subjects still alive without radiological progressive disease at data cut-off date will be censored at the last adequate tumor assessment date..

- The DCR and DoR are evaluated by the investigator based on the iRECIST criteria.
- OS is evaluated as the time from the date of first medication to the date of death from any cause.

For the subjects who are alive at the last follow-up, the OS of the subjects is censored based on the time of the last follow-up. For the subjects lost to follow-up, OS is considered the last confirmed survival before loss to follow-up. The OS with censored data is defined as the time from the first medication to censoring.

- Safety

(1) Adverse events (AEs), including type, incidence, classification (based on the NCI-CTCAE v5.0 standard), severity, duration, and correlation with the study treatment;

(2) Abnormal values of laboratory indicators, such as type, incidence, classification (based on the NCI-CTCAE v5.0 standard), duration, etc.;

(3) Vital signs, including blood pressure, pulse, respiratory rate, and ECOG-PS score;

Tolerability:

- Proportion of dose suspension, dose reduction and termination during the trial due to trial drug-related toxicity.

**Exploratory endpoints**

- Correlation between changes in HER2, EGFR, VEGFR2, vessel density, and PD-L1 and radiomics and efficacy.
- Correlations between the increase of AFP from the baseline and the decreasing proportion of AFP and ORR, PFS, and OS.

# Clinical trial design

## Description of the clinical trial design

This is a prospective, open, multicenter nonrandomized phase II clinical trial designed to evaluate efficacy and safety of camrelizumab combined with apatinib-based treatment for patients with unresectable/recurrent or metastatic AFPGC.

### Determination of sample size

This is a prospective, open-label, multicenter, phase II nonrandomized controlled clinical trial with two prospective intervention groups. The primary endpoint of this study is the ORR, and the Simon two-stage optimization design (Simon, 1989) is used.

Intervention group 1: A previous retrospective analysis of 105 patients showed an ORR of 30% in first-line chemotherapy for AFP-G/GEJ cancer patients with ~~an~~ AFP concentration ≥ 160 ng/ml. The null hypothesis H_0_ is that ORR = 30%. The alternative hypothesis H_1_ is that ORR = 55%. The one-sided type-I-error is 5% and statistical power is 80%. Nine subjects will be enrolled in the first stage. Among these 9 subjects, if at least four patients achieve a comfirmed objective response (CR or PR per RECIST 1.1), the study will proceed to the second stage and continue to enroll another 26 subjects. Among all 35 subjects, if at least 15 patients achieve comfirmed CR or PR, the null hypothesis will be rejected, and the result will deemed statistically significant.

Intervention group 2: The standard second-line treatment for GC is paclitaxel/docetaxel/irinotecan monotherapy. Previous analysis showed an ORR of 15-25% approximately. Since AFP elevation is an unfavorable prognostic factor in GC, null hypothesis H_0_ is set as ORR= 10%, and alternative hypothesis H_1_ is ORR = 30%. The one-sided type-I-error is 5% and the statistical power is 80%. Ten patients will be enrolled in the first stage. If among these 10 patients at least two achieve comfirmed CR or PR per RECIST 1.1, the study will proceed to the second stage and continue to enroll another 19 subjects. Among all 29 subjects, if at least 6 patients achieve CR or PR, the null hypothesis will be rejected, and the result will be deemed statistically significant.

A total of 64 patients are expected to be enrolled in the two groups, with 35 subjects in intervention group 1 and 29 subjects in intervention group 2. A total of 19 subjects will be enrolled in the two intervention groups in the first stage, and 45 subjects will be enrolled in the two intervention groups in the second stage.

## Overall design

The study includes patients who had unresectable/recurrent or metastatic G/GEJ adenocarcinoma with an AFP higher than 2 times the upper limit of the normal value or who are positive for AFP by immunohistochemistry, including patients who had not received advanced systemic chemotherapy and who failed at least first-line treatment. For patients who had not received advanced systemic chemotherapy, first-line treatment with camrelizumab combined with apatinib and SOX (S-1 capsules combined with oxaliplatin) is given. If there is no clear disease progression after four cycles, patients received maintenance treatment consisting of camrelizumab combined with apatinib until disease progression (intervention group 1); patients who failed at least first-line treatment are given second-line or above treatment consisting of camrelizumab combined with apatinib until disease progression (intervention group 2). The efficacy and safety of camrelizumab combined with apatinib in the two intervention groups will be observed. This study will also monitor the dynamic changes in serum AFP, biomarkers (such as HER2, EGFR, and VEGFR2), and radiomics to explore the predictive value of changes in AFP for efficacy and the correlation between biomarkers and treatment efficacy.

### Study Treatment

**Intervention Group 1: AFPGC patients who have not received advanced systemic chemotherapy**

Camrelizumab combined with apatinib and SOX

If there is no clear disease progression after four cycles, maintenance treatment with camrelizumab combined with apatinib will be started.

**Intervention Group 2: AFPGC patients who failed at least first-line treatment**

Camrelizumab combined with apatinib

**The specific dosage and medication were as follows:**

Camrelizumab: 200 mg, intravenous drip (infusion should be completed within 30-60 minutes), d1, 21 days as one cycle;

Apatinib: 250 mg, orally, Qd (orally 0.5 h after a meal, and the time of daily dosing should be the same as possible); 21 days as one cycle;

S-1 capsule: initial dosing of S-1 determined by body surface area (BSA <1.25m^2^, 40mg bid; 1.25 m^2^ ≤ BSA ≤ 1.5 m^2^, 50 mg bid; BSA>1.5 m^2^, 60 mg bid), 2 times a day, orally after breakfast and dinner, d1-14; 21 days as a cycle;

Oxaliplatin at 130 mg/m^2^ was intravenously infused for 2-4 hours, d1; 21 days as one cycle.

While receiving camrelizumab in combination with chemotherapy, the subjects will be given intravenous infusions of camrelizumab on the first day of each cycle, followed by an interval of at least 30 minutes and then intravenous infusions of oxaliplatin.

Treatment can be terminated for disease progression, intolerable toxicity, or other reasons for treatment termination.

### Imaging evaluation

All lesions are recorded and evaluated according to RECIST 1.1. The imaging examinations includes chest CT and enhanced CT scans of the abdomen and pelvis. If there is an allergy to the contrast agent on enhanced CT, a plain CT scan of the chest and an MRI scan of the abdomen and pelvis will be performed; patients with suspected brain metastases will also undergo cranial-enhanced MRI or enhanced CT to rule out brain metastases. Bone scan is required when definite or clinically suspected bone metastasis occurred.

During the treatment period, imaging evaluations will be done every 6-8 weeks until radiological progression, the receipt of other antitumor treatments, the withdrawal of informed consent, the loss to follow-up, or the death of the patient.

If treatment is discontinued during the treatment period due to reasons other than radiological disease progression, imaging examination should be done when treatment is completed, unless imaging evaluation has been performed within 28 days. For the subjects who still did not have radiological progression at the end of trial, the follow-up frequency during the tumor progression and survival period should be consistent with the follow-up frequency during the treatment period, and imaging assessments should be performed every 6-8 weeks until radiological disease progression, the receipt of other antitumor treatments, the withdrawal of informed consent, the loss to follow-up, or the death of the patient.

The allowed time window for each imaging examination is ±7 days unless otherwise stated. Unplanned imaging examinations may be performed when disease progression (such as worsening symptoms) is suspected.

### Survival follow-up

After the end of treatment, the subjects will undergo survival follow-up once every 3 months. The survival status and antitumor treatment data of the subjects will be collected and recorded until death or loss to follow-up.

### Safety follow-up

Safety visits are conducted on d1 of each treatment cycle, at the end of the study treatment, and 30 days (±7 days) after the end of the study treatment.

### Sample collection

Sample collection: Blood samples will be collected from the subjects during the screening period (-7 d to -1 d), at each imaging evaluation during the treatment period, and at the time of disease progression, with 2 ml (anticoagulated) and 5 ml (non-anticoagulated) of blood at each time for the detection of blood levels of AFP, and for the exploration of predictive markers of efficacy by NGS. In addition, 10 paraffin-embedded pathological tissue sections from the subjects before the first treatment and at the time of disease progression will be collected whenever possible for the detection of HER2, EGFR, and VEGFR2. The PD-L1 expression level, blood vessel density, and other indicators will also be measured.

After the end of the study, blood samples will be retained for at least 2 years and tissue samples will be retained for at least 5 years, then destroyed according to standard procedures for the destruction of biological samples. The results gained from biological samples will be used to explain scientific issues such as the occurrence and progression of the disease and its correlation with the efficacy of treatment.

### Trial design chart

The clinical trial design is shown in Figure 2.

Figure 2 Diagram of the clinical trial design


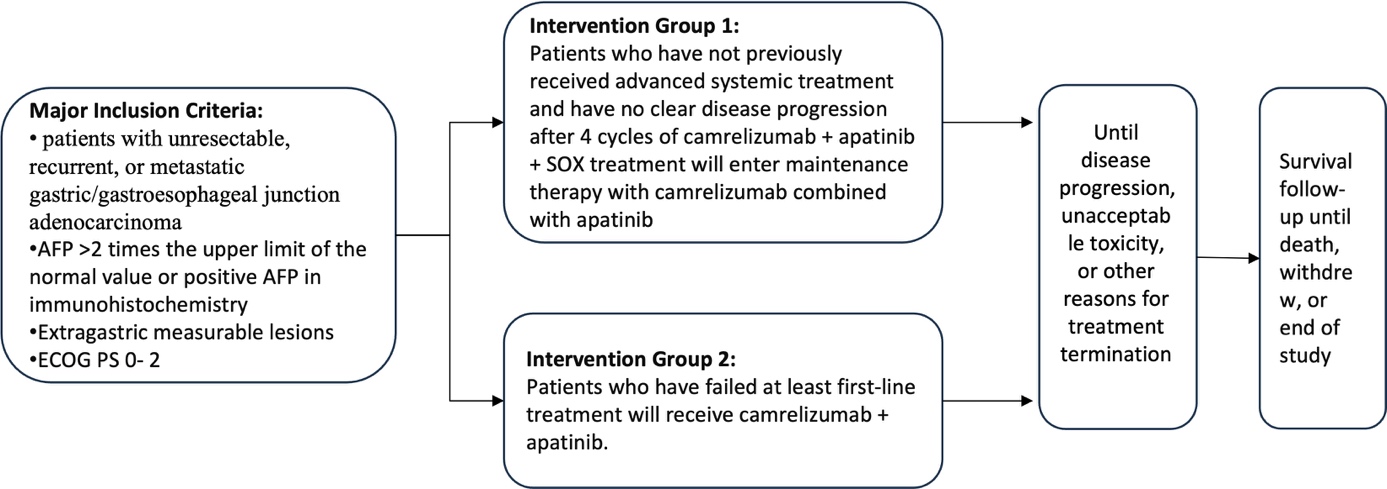


# Subject selection and withdrawal from the trial

Subjects who meet the following criteria are allowed to participate in the study. All medical and non-medical conditions of each subject will be taken into account in their compliance or non-compliance with the trial criteria. Before the subjects are included in the trial, the investigators will verify and record their suitability for the study.

## Inclusion criteria

Patients who meet all the following inclusion criteria will be included in this trial:

1. Patients who voluntarily joined the trial and sign the informed consent;
2. Male or female, aged ≥ 18 years;
3. ECOG PS score 0-2;
4. Pathologically confirmed gastric/gastroesophageal junction adenocarcinoma;
5. Clinical staging will be performed based on enhanced CT/MRI (combined with ultrasound gastroscopy and diagnostic laparoscopy when necessary). Patients with unresectable locally advanced or advanced disease with stage III-IV (according to the 8th edition of the AJCC TNM staging system for gastric cancer), and the possibility of radical surgery will be discussed and determined by a multidisciplinary team.
6. Patients who had not received advanced systemic treatment (intervention Group 1); patients who had received at least first-line systemic treatment (intervention Group 2);

Note: Neoadjuvant therapy is not included in the number of treatment lines. For patients who relapse within 6 months after the end of adjuvant therapy, the adjuvant therapy part is defined as first-line treatment; for patients who relapse more than 6 months after the end of adjuvant therapy, the adjuvant therapy part is not included in the number of treatment lines.

1. Extragastric measurable lesions (RECIST 1.1 criteria);
2. AFP >2 times the upper limit of the normal value (ULN) or a positive immunohistochemical AFP result;
3. Baseline complete blood count and biochemical indicators meeting the following criteria (the subjects have no blood transfusion or blood products within 14 days before the first medication and are not corrected by granulocyte–macrophage colony-stimulating factor (G-CSF) or other hematopoietic stimulatory factors):

Hemoglobin≥90 g/L;

Absolute neutrophil count (ANC) ≥1.5×10^9^/L;

Platelet ≥80×10^9^/L;

ALT and AST ≤2.5 × ULN or, if the patient had liver metastasis, ALT and AST ≤5 × ULN;

Total serum bilirubin (TBil) ≤ 1.5 × ULN;

Serum Cr ≤ 1.5 × ULN or endogenous creatinine clearance >50 ml/min (male: endogenous creatinine clearance=((140-age) × body weight)/(72×serum Cr); female: endogenous creatinine clearance rate=((140-age) × body weight)/(72× serum Cr) × 0.85; body weight unit: kg; serum Cr unit: mg/mL);

Serum albumin ≥30 g/L;

1. No serious concomitant disease causing survival <5 years;
2. Patients who can swallow medicine normally;
3. Patients who have good compliance and adhere to the regimen during the study;
4. Women who have a negative serum pregnancy test result within 72 h before the first treatment and who agree to use highly effective contraception during the trial and for 90 days after the end of treatment; all patients agree to use highly effective contraception during the treatment and for 90 days after the end of treatment;
5. They agree to provide blood and/or histological samples.

## Exclusion criteria

Patients who meet any of the following criteria cannot be included in this study:

- 1. Pregnancy or breastfeeding women;
  2. Known HER2 positivity (intervention Group 1; HER2 positivity was defined as IHC 3+ or IHC2+/ISH+);
  3. Adverse events (except for alopecia) caused by previous treatment that were not relieved to Grade ≤1 (CTCAE v5.0);
  4. History of other malignant diseases in the past 5 years or at the same time, except for cured basal cell carcinoma of the skin and carcinoma in situ of the cervix;
  5. For patients with a history of uncontrolled epilepsy, central nervous system diseases or mental disorders, the investigators should determine whether the clinical severity prevents the signing of informed consent or affects patient compliance with oral medications;
  6. Clinically serious (i.e., active) heart disease that is not well controlled, such as (1) symptomatic coronary heart disease, (2) New York Heart Association (NYHA) class II or more severe congestive heart failure or severe arrhythmias requiring drug intervention, (3) history of myocardial infarction in the past 12 months, (4) QTc interval ≥450 ms in males or ≥470 ms in females, and (5) LVEF<50%;
  7. Arterial/venous thrombosis events, such as cerebrovascular accidents (including transient ischemic attack, cerebral hemorrhage, and cerebral infarction), deep venous thrombosis and pulmonary embolism, that occurred within 6 months;
  8. For patients who have clinically significant bleeding symptoms or a clear bleeding tendency within 3 months, such as gastrointestinal bleeding or hemorrhagic gastric ulcers, if fecal occult blood is positive during the screening period, re-examination can be performed; if re-examination is still positive, gastroscopy can be conducted when necessary based on the clinical judgment (except for patients who had received gastroscopy within 3 months before enrollment and excluded this condition);
  9. Known hereditary or acquired bleeding and tendency of thrombocytopenia (such as hemophilia, coagulation disorders, thrombocytopenia, etc.);
  10. Upper gastrointestinal obstruction, abnormal physiological function or malabsorption syndrome, which may affect the absorption of oral drugs;
  11. Previous gastrointestinal perforation, abdominal abscess, or intestinal obstruction in the past 3 months or imaging findings and clinical symptoms suggesting intestinal obstruction;
  12. Abnormal coagulation function (INR>2.0 or prothrombin time>16 s), bleeding tendency, or receiving thrombolytic or anticoagulant therapy (the preventive use of small doses of aspirin, low-molecular-weight heparin, etc., is permitted);
  13. Patients with neurotoxicity caused by chemotherapy who were unsuitable for the use of oxaliplatin determined by the investigators cannot be included in intervention Group 1; however, patients who only exhibited disappearance of the deep tendon reflex (DTR) did not need to be excluded.
  14. Immunosuppressive therapy after organ transplantation or immunosuppressive drugs or systemic corticosteroids to achieve the immunosuppressive purpose within 14 days before treatment (such as >10 mg/day prednisone or other drug equivalent doses);
  15. Active ulcers, unhealed wounds, or fractures;
  16. Hypertension that cannot be well controlled by antihypertensive drug treatment (systolic blood pressure ≥140 mmHg or diastolic blood pressure ≥90 mmHg);
  17. Known dihydropyrimidine dehydrogenase (DPD) deficiency (intervention Group 1);
  18. Known allergies to any trial drugs or excipients;
  19. Previous treatment with PD1/PD-L1 or apatinib;
  20. Urinalysis suggesting a proteinuria concentration ≥ ++ and a confirmed 24-hour proteinuria concentration >1.0 g;
  21. Serous effusion with clinical symptoms (including ascites, pleural effusion, and pericardial effusion) that require symptomatic treatment. Asymptomatic patients with serous effusion are allowed to be included. Patients with symptomatic plasmapheresis that is well controlled by active management such as drainage are allowed to be enrolled at the judgment of the investigator;
  22. Active hepatitis (hepatitis B reference: HBsAg positive and HBV DNA ≥ 500 IU/ml; hepatitis C reference: anti-HCV positive and HCV viral copy number > ULN); patients in the active infection stage and requiring antimicrobial treatment (e.g., antibacterial drug therapy, antifungal drug therapy);
  23. Interstitial pneumonia, interstitial lung disease, or other diseases that may interfere with the determination and management of immune-related pulmonary toxicity, such as lung fibrosis, organizing pneumonia, pneumoconiosis, drug-associated pneumonia, idiopathic pneumonia, or screening CT showing active pneumonia or severe pulmonary function impairment; patients with active tuberculosis;
  24. Active autoimmune diseases or a history of autoimmune diseases and possible relapse (including but not limited to immune hepatitis, interstitial pneumonia, uveitis, enteritis, hypophysitis, vasculitis, nephritis, hyperthyroidism, hypothyroidism [subjects whose conditions are controllable by hormone replacement therapy can be included]). Patients with skin diseases that do not require systemic treatment (such as vitiligo, psoriasis, alopecia), type I diabetes that is controllable by insulin therapy, or a history of childhood asthma that is in complete remission without any intervention can be enrolled, though asthma patients requiring a bronchodilator cannot be enrolled.
  25. Other factors that, in the investigators’ judgment, may affect the trial results or cause the study to be stopped halfway, such as alcohol abuse, drug abuse, and other serious diseases (including mental illness) that require combined treatment, as well as seriously abnormal laboratory results, family factors, or social factors that can affect the safety of the patient.

## Withdrawal from the trial or termination of the treatment

### Criteria for termination of trial treatment

Termination of trial treatment does not imply withdrawal from the trial. Subjects who terminate the trial treatment must come to the remaining visits as required by the protocol. If the subject meets any of the following criteria, the trial treatment is terminated:

1. The subject voluntarily withdraws from treatment, terminates the trial medication, or withdraws informed consent;
2. The disease progresses based on the RECIST v1.1 criteria:

Note: Patients who had first disease progression, but were in clinically stable condition based on the investigators’ judgment, could continue treatment until progression was confirmed at a subsequent evaluation 4-8 weeks later.

1. The patient cannot tolerate the drug toxicity;
2. Significant protocol deviations such as the ineligibility of the subjects are found after enrollment (the decision will be discussed with the sponsor);
3. The subject does not comply with the protocol;
4. The subject is lost to follow-up;
5. The investigator deems that other circumstances make it necessary to terminate the trial medication;
6. The subject is pregnant.

### Criteria for withdrawal from the trial

Subjects may voluntarily withdraw from the trial at any time or may be required by the investigator or the sponsor to withdraw from the trial for safety or behavioral reasons or the inability to comply with the trial visits or protocol. The reasons for a subject’s withdrawal from the trial may include the following:

1. The subject withdraws informed consent to participate in the trial and refuses further follow-up;
2. The investigator believes that it is necessary to withdraw the subject from the trial; for example, the subject may lose the ability to freely express his/her will due to imprisonment or isolation;
3. The subject is lost to follow-up;
4. The subject dies;
5. The trial is terminated;
6. The subject gets pregnant.

### Steps to withdraw from the trial or terminate the treatment

After the subjects’ participation in the trial is terminated, the follow-up schedule specified in the protocol will be followed. The efficacy and safety checks at the time of termination of treatment and the safety follow-up period must be completed, and the AEs as well as the outcomes and the combined drugs and treatments are comprehensively investigated. The investigator may suggest or provide new or alternative treatment methods to the subjects based on the actual situation of the subjects. For subjects without disease progression, imaging evaluations should continue to be performed according to the planned frequency and time until the subject starts new antitumor treatment, disease progression, loss to follow-up, or death. We will attempt to obtain evidence of disease progression from such subjects.

If the subject refuses to come the research center for further visits, the survival status is tracked unless the subject withdraws consent to share any more information or to keep being contacted. If the subject clearly requests to withdraw from the trial, the investigator should record in writing the extent to which the subject requests to withdraw from the study steps, that is, only the withdrawal from the study treatment and/or the follow-up after the withdrawal from treatment. When determining survival status, only publicly available information will be appropriately used to determine whether the subject is alive.

### Loss to follow-up

All efforts must be made to know and report the status of each subject, including contacting the subject. Loss to follow-up is defined as no response to at least three contact events on different days. Contact methods include but are not limited to any of the following: telephone, fax, text message, social media tools, and email. All attempts to make contact are recorded in the medical file. If it is determined that the subject has died, the research center will use the permitted methods to obtain death information and the cause of death. The research centers may also use public resources, such as community health registries and databases, to obtain contact information. If the subject’s status still cannot be obtained after all the attempts, the investigator should report the date when the subject was last known alive and record it in the subject’s medical history.

## Early termination or suspension of the trial

If there are good reasons, the study can be terminated or suspended early. This may be due to the decision of the regulatory agency, the change in the opinion of the ethics committee, the efficacy or safety of the trial drug, or the judgment of the sponsor. The party that decides to terminate or suspend the trial will send a written notification to the investigator, the sponsor, and the regulatory agency listing the reasons for the termination or suspension. The investigator should immediately notify the ethics committee and the sponsor and provide relevant reasons.

The reasons for the early termination or suspension of the trial may include the following:

1. Unexpected, significant, or unacceptable risks to patients are identified;
2. Major errors in the protocol are discovered during the trial execution;
3. The trial drug/treatment is not effective, or it is pointless to continue the trial;
4. Due to reasons such as severe lag in patient enrollment or frequent protocol deviations, the sponsor decides to terminate the study.

Once the abovementioned drug safety, regimen adherence, and data quality problems that causes the suspension of the trial are resolved and approval is obtained from the sponsor and the ethics committee, the study can continue.

## Definition of end of the trial

Definition of the end of the trial: The study ended after the final analysis.

# Trial drugs

## Overview of the trial drugs

The trial drugs are apatinib mesylate tablets, camrelizumab, S-1 capsules, and oxaliplatin. For all treatment cycles, according to the judgment of the investigator, for management reasons, the drug is administered before or within 3 days after the planned first day of each cycle.

|  | Apatinib mesylate Tablets | Camrelizumab | S-1 Capsules | Oxaliplatin |
| --- | --- | --- | --- | --- |
| Manufacturer | Jiangsu Hengrui Pharmaceutical Co., Ltd | Jiangsu Hengrui Pharmaceutical Co., Ltd | -- | -- |
| Dosage form | Tablet | Lyophilized powder for injection | Capsule | Powder for injection |
| Specifications | 250 mg | 200 mg | 20 mg/25 mg | 50 mg |
| Route of administration | Oral | Intravenous injection | Oral | Intravenous injection |

### Drug storage and stability

Apatinib tablets and oxaliplatin need to be protected from light, sealed from air, and stored below 25 °C. The S-1 capsules are sealed and stored at room temperature. Camrelizumab should be protected from light, sealed, and stored in a cool place (2–8°C), not frozen. The investigator or an authorized representative (e.g., pharmacist), will ensure that all trial drugs are stored in a secure area that meets the storage conditions, has controlled access, and meets applicable regulatory requirements.

Research centers must be able to record the maximum and minimum temperature of each working day for all trial drug storage locations. The recording period should start from drug reception to the time that all the remaining trial drug is returned. Even with a continuous monitoring system in place, research centers should have record logs to ensure the correct storage temperature. The temperature monitoring devices and storage devices (such as incubators) should be periodically checked to ensure normal operation.

Any deviation from the product labeling conditions should be reported promptly. The research center should take active measures to store the product under the storage conditions stated on the label as soon after receiving it as possible and to report to the sponsor any temperature deviation and remedial measures taken. The trial drugs affected by temperature deviation should be temporarily isolated in an environment that is suitable for drug storage and cannot be used further until the sponsor gives permission. The sponsor will provide the research centers with specific steps for reporting temperature deviation.

### Dosage adjustment

Every effort should be made to administer the trial drugs to each subject according to the requirements of the protocol. If it is necessary to suspend the trial treatment and/or adjust the dosage due to adverse drug reactions, the reasons for the suspension/adjustment should be recorded.

General principles for dosage adjustment:

- If the dosing delay/adjustment is caused by chemotherapeutic drugs in combination therapy or the toxicity of camrelizumab or apatinib, the planned use of other drugs in combination therapy is not affected.
- The investigator may delay all combination treatment (not more than 7 days) according to the actual situation to maintain the synchronization of the study treatments.
- The interval between doses of camrelizumab should not be shorter than 12 days;
- The discontinuation of chemotherapeutic drugs, camrelizumab, or apatinib should not affect the continued use of other trial drugs;
- For subjects receiving apatinib, camrelizumab, S-1, or oxaliplatin, the latest version of the medication package insert or medical practice should be referred to for dose adjustment.

The subjects may suspend treatment due to other medical, surgical, or accident events that are not related to the trial treatment unless the investigator and the sponsor discuss other resolutions. The reasons for the suspension of treatment must be recorded.

If the drug cannot be administered on the original medication date due to special circumstances such as holidays, the drug can be administered at a convenient time that is closest to the original medication date. The date of the next treatment cycle will be calculated from the actual date of medication.

#### Dosing adjustment of camrelizumab

##### Criteria of dosing adjustment of camrelizumab

Increases or decreases in the dose of camrelizumab are not allowed. If camrelizumab treatment is suspended due to toxicity, the subject can continue to receive treatment at the original dose when the toxicity is relieved. The adverse event associated with camrelizumab may be immunotoxicity, which may occur within a short time after the first dose or a few months after the last dose. If the situations listed in Table 1 occur, camrelizumab should be suspended. If, in clinical practice, the investigators, considering the benefit/risk ratio of the subjects, believe that the operations listed in Table 1 are not suitable or that the conditions not listed in the table occur and that the investigators need to suspend or resume camrelizumab, they should discuss the decision with the sponsor.

##### During the trial, a maximum of 12 weeks (four cycles) of camrelizumab suspension is allowed. Criteria for dosing adjustment in camrelizumab administration

When camrelizumab is suspended due to camrelizumab-related toxicity, camrelizumab administration can be resumed when the toxicity is reduced to level ≤1 or the baseline level is reached. (Adverse reactions such as alopecia and fatigue, which are not considered by the investigator to have a significant impact on the safety assessment, are excluded.) At this time, S-1, oxaliplatin, and apatinib can still be administered on the original treatment cycle. If camrelizumab-related toxicity is relieved within 7 days, camrelizumab can be used in the current treatment cycle, and the date of the next treatment cycle is determined based on the chemotherapy date of the current cycle. If camrelizumab-related toxicity cannot relieved within 7 days, camrelizumab should not be used in the current cycle. If it can relieve within 21 days, the combination can be used on d1 of the next scheduled treatment cycle (chemotherapy, camrelizumab, and apatinib).

The interval between administrations of camrelizumab should not be shorter than 12 days.

Table 1 Criteria for delayed administration of camrelizumab caused by immune-related toxicity

| **Camrelizumab related toxicity** | **AE grade for suspension of treatment** | **Criteria for restarting treatment** | **Termination of treatment** |
| --- | --- | --- | --- |
| Diarrhea/colitis | 2-3 | Recovery to grade 0-1 | Not relieved within 12 weeks after the last dose, or the dose of corticosteroids cannot be reduced to 10 mg/d or the less potent prednisone (or equivalent drug) within 12 weeks. |
|  | 4 | Termination of medication | Termination of dosing |
| Elevated AST/ALT or TBil | 2 | Recovery to grade 0-1 | Not relieved within 12 weeks after the last dose, or the dose of corticosteroids cannot be reduced to 10 mg/d or the less potent prednisone (or equivalent drug) within 12 weeks. |
|  | 3-4 | Termination of medication^a^ | Termination of medication |
| Type I diabetes (if new onset) or hyperglycemia with signs of β-cell failure | Grade 3-4 or new-onset type I diabetes | The use of camrelizumab should be suspended for new-onset type 1 diabetes or grade 3-4 elevated blood glucose combined with evidence of beta cell damage | Camrelizumab treatment should not be restarted until the clinical and metabolic status of the subjects stabilizes. |
| Hyperthyroidism | 3 | Toxicity reduced to grade 0-1 | Not relieved within 12 weeks after the last dose, or the dose of corticosteroids cannot be reduced to 10 mg/d or the less potent prednisone (or equivalent drug) within 12 weeks. |
|  | 4 | Termination of medication | Termination of medication |
| Hypothyroidism |  | After starting thyroid hormone replacement therapy, study treatment could be continued. | |
| Pneumonia | 2 | Toxicity reduced to grade 0-1 | Not relieved within 12 weeks after the last dose, or the dose of corticosteroids cannot be reduced to 10 mg/d or the less potent prednisone with lower potent (or equivalent drug) within 12 weeks. |
|  | 3-4 | Termination of medication | Termination of medication |
| Immune related hypophysitis | 2-4 | Toxicity decreased to Grade 0-1. After starting endocrine replacement therapy, camrelizumab treatment can be continued. | Not relieved within 12 weeks after the last dose, or the dose of corticosteroids cannot be reduced to 10 mg/d or the less potent prednisone with lower potent (or equivalent drug) within 12 weeks. |
| Renal failure or nephritis | 2 | Toxicity reduced to grade 0-1 | Not relieved within 12 weeks after the last dose, or the dose of corticosteroids cannot be reduced to 10 mg/d or the less potent prednisone with lower potent (or equivalent drug) within 12 weeks. |
|  | 3-4 | Termination of medication | Termination of medication |
| Infusion reactions | 2^b^ | Toxicity reduced to grade 0-1 | If symptoms return after adequate prophylaxis, the dosing should be terminated. |
|  | 3-4 | Termination of medication | Termination of medication |
| Other drug-related toxicity^c^ | 3 | Toxicity reduced to grade 0-1^d^ | Not relieved within 12 weeks after the last dose, or the dose of corticosteroids cannot be reduced to 10 mg/d or the less potent prednisone with lower potent (or equivalent drug) within 12 weeks. |
|  | 4 | Termination of medication | Termination of medication |

For any recurrent grade ≥3 drug-related adverse events (pneumonia grade ≥2) or any life-threatening adverse events, the medication will be terminated.

1. For the subjects with liver metastasis at the start of study treatment and with a grade 2 increase in ALT/AST, if the increase in ALT/AST is ≥50% of the baseline level and lasts at least 1 week, treatment will be terminated.
2. If symptoms are relieved within 1 hour of temporary discontinuation of treatment, the infusion can be continued at 50% of the initial infusion rate. Otherwise, the symptoms should be completely relieved before continuing to administer the drug. The subjects also need to be fully given prophylactic medication when the next medication is administered. For further treatments, please see the recommendations in Section 5.4.2 on the management of infusion reactions.
3. For subjects who have intolerable or persistent grade 2 drug-related adverse events, the investigators may appropriately suspend camrelizumab. If the toxicity does not decrease to Grade 0-1 or baseline within 12 weeks after the last dose, the treatment should be terminated.
4. If the subject has grade 2 fatigue, the medication can be continued; if the subject has grade 2 skin toxicity (without grade ≥ 3 skin toxicity), the medication can be continued;
5. Most drug-related grade 4 toxicities require the discontinuation of camrelizumab, with the following exceptions:
   1. Grade 4 neutropenia for ≤7 days;
   2. Grade 4 lymphopenia or grade 4 leukopenia;
   3. Grade 4 amylase or lipase abnormalities without clinical symptoms or not caused by pancreatitis;
   4. Grade 4 electrolyte imbalance/abnormality, which can be corrected within 72 hours after effective treatment;
   5. Grade 4 drug-related endocrine disorders were fully controlled with hormone replacement therapy at a physiological dose.

#### Adjustment for apatinib medication

The dosage adjustments of apatinib include dose suspension, medication adjustment (1 day on, 1 day off), and medication termination. When severe AEs (hematological or nonhematological toxicity* grade ≥3) occur during the trial, the administration of apatinib will be stopped, and the dosage of apatinib will be adjusted to qod (1 day on, 1 day off after the toxicity is relieved. If the tolerability to apatinib is still poor after the adjustment to qod, the medication should be suspended when the first severe AE occurs, and qod medication should be resumed after the toxicity is relieved. If severe AE occurs again, apatinib should be terminated. After the dosage of apatinib is adjusted during the study period, no callback is allowed.

* Among the nonhematological toxicities, manageable nausea, vomiting, fever with definite causes (such as infection or tumor), and elevated AKP of the third/fourth degree can be treated with aggressive symptomatic management and therapy without dose suspension or dose reduction.

#### Medication adjustments for S-1 and oxaliplatin

The dosage of chemotherapeutic drugs is adjusted as follows:

1. The initial dose of each chemotherapeutic drug should be calculated from the baseline weight of the subject. If during the study the subject’s body weight changes by >10% (from baseline), the dose is recalculated; if the subject’s body weight changes ≤10%, the dose does not need to be adjusted (unless for drug-related toxicity).
2. Chemotherapy drugs that are missed and not used due to toxicity do not need to be replenished during this treatment cycle.
3. For the use of chemotherapeutic drugs that are suspended due to chemotherapy-related toxicity, the chemotherapy can be resumed when the toxicity falls to grade ≤1 or the baseline level (except for adverse reactions such as alopecia and fatigue that the investigator considers has no significant impact on safety). If chemotherapy-related toxicity is relieved within 7 days (including 7 days), chemotherapy can be used in the current treatment cycle, and the date of the next treatment cycle is counted from the chemotherapy administration date. If chemotherapy-related toxicity cannot be relieved within 7 days, chemotherapy will not be given in the current cycle; if it can be relieved within 21 days, combination therapy (chemotherapy, camrelizumab and apatinib) can be started on d1 of the next treatment cycle.
4. For nonserious toxicity or nonfatal toxicity (such as alopecia, changes in appetite, and nail discoloration), dose adjustment or suspension of medication may not be made after the decision of the investigator.
5. If multiple toxicities associated with dose adjustment occur simultaneously, the dose should be adjusted based on the highest grading of CTCAE v5.0.

##### Dosage adjustment of S-1

The recommended dosage adjustment criteria for S-1 are shown in Table 2. If any situation meets the dose suspension criteria during the administration of S-1, S-1 will be suspended. If the medication criteria are fully restored in the subsequent cycle, the medication can be resumed, and the dose should not be adjusted after being lowered.

Table 2 Criteria for dose suspension/restarting of S-1

| Adverse reaction | Dose suspension criteria | Criteria for restarting dosing |
| --- | --- | --- |
| Neutropenia | Grade 3 or 4 | Above 1.0× 10^9^/L |
| Thrombocytopenia | Grade 3 or 4 | Above 50× 10^9^/L |
| Elevated serum creatinine | More than 1.5 × ULN | Below ULN |
| Infection | Have an infectious fever of 38°C or higher | No infectious fever |
| Diarrhea, oral ulcer | Grade 3 or 4 | Grade 1 and below |
| Other | When other adverse reactions occur and the investigator determines that it is difficult to continue medication, the treatment can be discontinued. | If other adverse reactions that led to the discontinuation of treatment were relieved, the investigator determined that the medication could be restarted. |

After the second cycle, the dose reduction of S-1 can be performed in the next cycle according to the dose reduction standards, but each time, the dose can only be reduced by one dose level, and the dose can be reduced at most two times. Tables 3 and 4 show the amount reduction levels and standards.

Table 3 Dose reduction levels of S-1

| Initial dose | Level of reduction | | | | |
| --- | --- | --- | --- | --- | --- |
| 80 mg/d | 50 mg/d | → | 40 mg/d | → | Withdrawal of treatment |
| 100 mg/d | 80 mg/d | → | 50 mg/d | → | Withdrawal of treatment |
| 120 mg/d | 100 mg/d | → | 80 mg/d | → | Withdrawal of treatment |

Table 4 Standards for dose reduction of S-1

| Adverse reaction | Grade of adverse reaction | Level of reduction |
| --- | --- | --- |
| Leukopenia | Grade 4 | Reduce dose by 1 level |
| Neutropenia | When the medication criteria for the next cycle are not met within Day 15 ^*^ | Reduce dose by 1 level |
| febrile neutropenia | Grade 3 and above | Reduce dose by 1 level |
| Thrombocytopenia | Grade 4 | Reduce dose by 1 level |
|  | When the medication criteria for the next cycle are not met by d15 ^*^ | Reduce dose by 1 level |
| Diarrhea | Grade 3 and above | Reduce dose by 1 level |
|  | When the medication criteria for the next cycle are not met by d15 ^*^ | Reduce dose by 1 level |
| Allergies | Grade 3 and above | – |
| Other |  | When adverse reactions that do not meet the above discontinuation criteria are found and the medication can no longer be continued, the investigator may reduce the dose if necessary. |

^*^Delays are allowed for up to 4 days (Day 18).

##### Dosage adjustment of oxaliplatin

Oxaliplatin-induced toxicity can be managed by symptomatic treatment and/or treatment interruption or dose reduction. The recommended dosage adjustment criteria for oxaliplatin are shown in Table 5. Once the dose is decreased, it cannot be adjusted again.

Table 5 Recommended principles for dosage adjustment of oxaliplatin

| Adverse reaction | Dosage adjustment |
| --- | --- |
| Neurotoxicity | - The use of oxaliplatin could be suspended for grade 2 peripheral sensory neurotoxicity (moderate paresthesia or dysesthesia) or limitation of the use of tools in daily life. When the toxicity is relived to grade ≤1, the dose of oxaliplatin can be adjusted to 75% of the initial dose and the continued to use. If oxaliplatin is discontinued for more than 4 weeks (2 consecutive administrations) due to neurotoxicity, oxaliplatin should be permanently terminated. - For grade ≥ 3 peripheral neurotoxicity (severe paresthesia or dysesthesia) or limitation of self-care activities, oxaliplatin treatment should be permanently discontinued. |
| Renal impairment | - For mild or moderate renal impairment (CrCl > 50 ml/min), there is no need to adjust the dose of oxaliplatin. - For severe renal impairment, the dose of oxaliplatin should be adjusted to 75% of the initial dose |
| Hematological toxicity | - For grade 2 or 3 thrombocytopenia, the dose of oxaliplatin should be adjusted to 75% of the initial dose. For grade 4 thrombocytopenia, the dose of oxaliplatin should be adjusted to 50% of the initial dose. - For grade 3 or 4 neutropenia or febrile neutropenia, the dose of oxaliplatin should be adjusted to 75% of the initial dose. |

##### Termination criteria for S-1 and oxaliplatin

If subjects experience the following chemotherapy-related adverse events, the use of corresponding chemotherapeutic drugs should be discontinued:

- Oxaliplatin should be terminated for grade ≥ 4 peripheral neurotoxicity.
- Oxaliplatin should be terminated for persistent paresthesia ≥ Grade 3.
- For grade ≥ 3 chemotherapy-related skin/mucosal adverse reactions, S-1 and oxaliplatin should be discontinued.
- For grade ≥ 3 chemotherapy-associated thrombocytopenia combined with clinically significant bleeding, S-1 and oxaliplatin should be terminated.
- For patients with chemotherapy-related abnormal liver function, S-1 and oxaliplatin are terminated if any of the following criteria are met:
  - ALT/AST > 5-10 × ULN for >2 weeks;
  - ALT/AST > 10 × ULN;
  - TBil > 5 × ULN;
  - ALT/AST >3 × ULN and TBil >2 × ULN.
- When oxaliplatin causes creatinine clearance < 30 ml/min (calculated using the Cockcroft-Gault formula), oxaliplatin should be terminated.
- After two dose adjustments (reductions), if the same chemotherapy-related adverse events still occurred, the corresponding chemotherapeutic drugs are terminated.
- For grade ≥ 3 allergic reactions or infusion reactions caused by chemotherapeutic drugs, those chemotherapeutic drugs should be terminated. If the investigator determines that the allergic reaction/infusion reaction is not caused by chemotherapeutic drugs, the chemotherapeutic drugs can be continued.
- For any grade 4 chemotherapy-related adverse events, if the investigator believes that dosage adjustment is not suitable, termination of the corresponding chemotherapeutic drug can be considered.
- For any chemotherapy-related adverse events that cannot be relieved within 42 days, the corresponding chemotherapeutic drugs should be discontinued.

## Drug management, distribution and return

The management, distribution, and return of the trial drug are the responsibility of the assigned personnel. The investigators must ensure that all trial drugs are used only by the subjects participating in the clinical trial, and the dosage and usage should be in accordance with the trial protocol. Any remaining drugs or expired drugs were returned to the sponsor, and clinical medications cannot be transferred to any nonclinical trial participant.

The trial drugs are stored according to the drug storage conditions (refer to the drug information for details). When receiving drugs, the drug receiving form must be signed by two people, and the notice should be signed in duplicate. The clinical research unit and the sponsoring unit each held a copy. At the end of the study, the remaining drugs and empty boxes are removed, and the two parties sign the drug return form. The distribution and return of each drug should be recorded in a timely manner on the special record sheet.

The monitor is responsible for monitoring the supply, use, and storage of the clinical trial drugs and the disposal process of the remaining drugs.

## Combined medication/concomitant treatment

Combined medication/concomitant treatment are other drugs/treatments that are given at the discretion of the investigators and in the best interest of the subjects.

The combined medication/concomitant treatment from the time when the subjects signed up for the trial to the end of the safety follow-up period will be recorded. Once the subject discontinues the trial treatment, only the combined medications and concomitant treatments used for the new or unrelieved adverse events related to the trial treatment are recorded, and all the combined medications, blood products, and nondrug interventions (e.g., puncture) received should be recorded in the case report form in strict accordance with the regulations of the GCP.

Drugs or treatments specifically prohibited by the study protocol are prohibited throughout the trial. If the subjects have comorbidities that require the use of prohibited drugs, they may need to stop the trial drug treatment or receive a protocol-prohibited drug. The investigators need to contact the sponsor to make such a change. After discussion, the decision of subjects to continue to participate in the study or to receive protocol-prohibited drugs is ultimately made by the investigator, the sponsor, and the subject.

In addition, for patients receiving camrelizumab, apatinib, S-1, or oxaliplatin, reference should also be made to the latest version of the medication package insert or medical practice conventions for contraindications and precautions.

### Prohibited drugs and treatments and drugs allowed with caution during the clinical trial

**All subjects:**

- During the study period, other systemic antitumor treatments are not allowed, including chemotherapy, molecular-targeted therapy, hormone therapy, immunotherapy, biological therapy, nonpalliative radiation therapy, and immunomodulators (including but not limited to interferons, interleukin-2, and thymosin).
- Patients may not participate in clinical trials of other drugs or devices.
- The concurrent use of flucytosine, a fluorouracil antifungal drug, is not allowed during the study.
- The simultaneous use of sorivudine or its structural analogs, such as brivudine, is not allowed during the study.
- G-CSF and granulocyte/macrophage colony-stimulating factor (GM-CSF) cannot be used as a means of primary prevention before treatment, but they can be used for adverse events. Colony-stimulating factors must be stopped 48 hours before the start of a chemotherapy.

**During treatment with camrelizumab:**

- The long-term systemic use of corticosteroids is not allowed, except for the treatment of immune-related adverse events (irAEs) according to the medication package insert. Within 14 days before the first treatment, subjects who require systemic treatment with corticosteroids (> 10 mg/d prednisone or other corticosteroids at the equivalent physiological dose) or other immunosuppressants will not be included in this trial. In the absence of active autoimmune disease, short-term use of topical or inhaled steroids is permitted.
- Concurrent use of other immunosuppressants during the trial period (except for treatment for drug-related adverse events) was not allowed.
- Live attenuated vaccines should not be used within 4 weeks before the first dose and during the trial.

**During treatment with apatinib:**

Preclinical studies have shown that apatinib is mainly metabolized by the hepatic enzyme CYP3A4. Therefore, strong inducers and strong inhibitors of CYP3A4 should be used with caution during the trial (including within 2 weeks before the first treatment) (Appendix 4). At the same time, during the trial, subjects should also be cautious about consuming foods with CYP3A4-inhibitory effects, such as grapefruit, bitter orange, and their products.

### Combined drugs and treatments allowed during the trial

#### Vaccine

Vaccines can be used to prevent infectious diseases, such as pneumonia and influenza, but these vaccines must be inactivated. Other vaccines must be discussed with the sponsor before use.

#### Immunomodulators and corticosteroids

Given the possibility of interfering with the pharmacodynamic activity of camrelizumab, the use of systemic corticosteroids and other immunosuppressants before starting camrelizumab should be avoided. To treat immune-related adverse reactions, systemic corticosteroids and other immunosuppressants can be used according to the medication package insert after starting camrelizumab treatment. The prophylaxis of chemotherapy or contrast agent allergy is allowed. Corticosteroids can be used for no more than 3 weeks to treat non-autoimmune diseases (such as delayed allergies caused by contact allergens).

Emergency use, topical application, spray inhalation, eye drops, or local injection of corticosteroids are permitted. Systemic corticosteroids (≤10 mg/d prednisone or its equivalent) may be used at a physiological replacement dose (such as an adrenal replacement steroid).

#### Hematopoietic growth factor

The use of G-CSF or GM-CSF as a means of primary prevention before treatment is not allowed, but its use for the treatment of adverse events is. The use of colony-stimulating factors must be stopped 48 hours before the start of subsequent chemotherapy.

Erythropoietin-stimulating agents (ESAs) can be used for cancer-related anemia. For anemia caused by chemotherapy, when hemoglobin (Hb) < 11 g/dL or ≥ 2 g/dL lower than the baseline level, erythropoietin can be used at the discretion of the investigator.

#### Anti-inflammatory treatment

If there is no known or predictable drug interaction and if the drugs are not prohibited by the protocol, anti-inflammatory or narcotic analgesics can be given.

### Drugs that should be used with caution in patients who received apatinib treatment during the trial

During treatment with apatinib, drugs that may affect the metabolism of apatinib or prolong the QT interval should be used with caution.

- **Drugs that may have drug‒drug interactions with apatinib mesylate**

In vitro metabolizing enzyme studies showed that apatinib is mainly metabolized by CYP3A4. The concomitant application of apatinib and strong CYP3A4 inhibitors (itraconazole, clarithromycin, voriconazole, telithromycin, saquinavir, ritonavir, etc.) may increase the plasma concentration of apatinib. When CYP3A4 inducers (dexamethasone, phenytoin, carbamazepine, rifampin, phenobarbital, rifapentine, etc.) are used, plasma apatinib may decrease. When combined with other drugs, alternative drugs that do not inhibit or induce CYP3A4 are recommended. If it is used at the same time as strong CYP3A4 inhibitors or inducers, the dose adjustment should be based on clinical observation.

- **Drugs that cause cardiac QT interval prolongation**

Because tyrosine kinase inhibitors have the toxic side effect of prolonging the QT interval in clinical practice, drugs that prolong the QT interval should be used with caution during the trial. These mainly include but are not limited to the classes of drugs listed below:

- Antibiotics: fluoroquinolones: sparfloxacin, gatifloxacin, levofloxacin, moxifloxacin, ofloxacin, ciprofloxacin; macrolides: erythromycin, clarithromycin, telithromycin, azithromycin, roxithromycin, metronidazole.
- Antiarrhythmic drugs: quinidine, procainamide, disopyramide, flecainide, propafenone, amiodarone, dronedarone, sotalol, dofetilide, ibutilide.
- Chronic heart failure treatment: ranolazine, ivabradine.
- Antipsychotics: risperidone, fluphenazine, droperidol, haloperidol, thioridazine, pimozide, olanzapine, clozapine.
- Antifungal drugs: voriconazole, posaconazole.
- Antimalarials: mefloquine, chloroquine.
- Antihistamines: terfenadine, astemizole, and hydroxyzine.
- Gastrointestinal drugs: antiemetics: ondansetron, granisetron, dolasetron, droperidol (0.625-1.25 mg is probably a safe dose), hydroxyzine; prokinetic drugs: cisapride, domperidone, metoclopramide
- Antidepressants: amitriptyline, imipramine, clomipramine, dothiepin, doxepin.

## Suggestions for symptomatic treatment of common adverse reactions to camrelizumab

The toxicity of immune checkpoint inhibitors (ICIs) is different from that of other types of antitumor drugs, each having its own particularities in severity and duration. These events can be divided into infusion reactions and irAEs. The adverse events caused by camrelizumab need to be identified and treated early to reduce the incidence of serious toxic events.

irAEs are defined as specific events that require individuals to receive immunosuppressant drugs for treatment (including pneumonia, diarrhea/colitis, hepatitis, nephritis/renal dysfunction, rash and endocrine diseases, etc.).

### Immuno-oncological drug safety management rules

The safety handling rules and relevant guidelines of similar products marketed in other countries (e.g., “Management of toxicities from immunotherapy: ESMO Clinical Practice Guideline for diagnosis, treatment and follow-up”) can be referred to to help investigators assess and handle adverse events in the following systems: gastrointestinal tract, kidney, lung, liver, endocrine, skin, and nerves (see Appendix 5 for details on the safety management rules of immuno-oncology drugs).

The general principle is to make a differential diagnosis according to standard medical norms, consider noninflammatory causes first and perform appropriate treatment. After other causes (such as disease progression, infection, and other drugs-related) are excluded, irAEs are considered, and immunosuppressant drugs are used for treatment. Autoantibody examination is usually helpful in diagnosis, but the role of tissue biopsy in the diagnosis of irAEs is still unclear. Ww recommended consulting a medical or surgical specialist, especially before invasive diagnostic or treatment procedures.

Corticosteroids are the main treatment for irAEs. Patients with low-grade toxicity who can walk on their own can consider using the oral equivalent of the recommended intravenous dose. When changing to an equivalent dose of oral corticosteroids, the low bioavailability of oral corticosteroids should be taken into consideration.

### Infusion reactions

Camrelizumab is a fully humanized mAb, so it is unlikely to cause infusion reactions, and prophylactic medication is generally not needed before infusion. Once an infusion reaction occurs, the infusion should be slowed or interrupted according to the patient’s situation, supportive clinical treatment should be given, and preventive medication should be given before the next medication.

Relevant symptoms and signs of acute infusion reactions usually occur during or shortly after drug infusion and usually disappear within 24 hours after the infusion is complete. Symptoms and signs include allergic reactions/hypersensitivity reactions (including drug-induced fever), cough, chills, shiver, dizziness, headache, fatigue (fatigue, lethargy), rash, itchy skin, joint pain, muscle pain, low or high blood pressure, nausea, vomiting, sweating, tachycardia, hives, difficulty breathing (shortness of breath), and bronchospasm.

Allergic reactions should be treated according to the medical practices and guidelines of the research institution. Symptomatic treatment (e.g., oral antihistamines or corticosteroids) should be used for the treatment of delayed hypersensitivity reactions (e.g., pruritus that occurs 1 week after the end of the infusion). The specific treatment recommendations for infusion reactions are shown in Table 7.

Table 7 Treatment recommendations for camrelizumab infusion reactions

| CTCAE grade | Clinical symptoms | Recommended treatment | Camrelizumab treatment |
| --- | --- | --- | --- |
| 1 | Mild transient reaction | Observe at the bedside and monitor closely until recovery. In the future, prophylactic treatment before infusion should be used: diphenhydramine 50 mg, or equivalent and/or acetaminophen 325-1,000 mg should be used at least 30 minutes before camrelizumab treatment. | Continue to use |
| 2 | Moderate reactions, which require treatment or the suspension of medication, are rapidly relieved after symptomatic treatment (such as antihistamines, nonsteroidal anti-inflammatory drugs, anesthetics, bronchodilators, intravenous infusion, etc.) | Saline IV, Benadryl 50 mg IV or equivalent and/or acetaminophen 325-1000 mg; bedside observation and close monitoring until recovery.  According to clinical needs, corticosteroids or bronchodilators may be considered;  The trial drug infusion volume is recorded in the original medical record.  In the future, prophylactic treatment before infusion should be used: diphenhydramine 50 mg, or equivalent and/or acetaminophen 325-1,000 mg should be used at least 30 minutes before camrelizumab treatment. If necessary, corticosteroids (equivalent to 25 mg of hydrocortisone) could be used. | Pause. When restarting the drug after the relieving of symptoms, use 50% of the initial infusion rate. If there is no complication within 30 minutes, the original infusion speed can be restored.  Monitor closely. If symptoms recur, no more infusion should be performed. |
| ≥3 | Grade 3: Severe reaction, no rapid relief after treatment and/or suspension of treatment; or recurrence of symptoms after remission; sequelae requiring hospitalization.  Grade 4: Life-threatening. | Stop the infusion of camrelizumab immediately.  An intravenous infusion of normal saline was started.  Bronchodilators are recommended, with subcutaneous injections of 0.2-1 mg of 1:1000 epinephrine solution or 0.1-0.25 mg of 1:10,000 epinephrine solution administered slowly intravenously. If necessary, diphenhydramine 50 mg as well as methylprednisolone 100 mg (or equivalent) may be given intravenously.  The medical practice and guidelines of the investigation institution for the treatment of anaphylaxis were followed. Patients were observe at the bedside and monitor closely until recovery. | Termination of treatment |

### Grading criteria and therapy recommendations for camrelizumab-induced reactive cutaneous capillary endothelial proliferation

Table 8 Grading criteria and treatment recommendations for camrelizumab-induced reactive capillary hyperplasia

| Grading | Clinical manifestation | Treatment suggestions |
| --- | --- | --- |
| 1 | Multiple or single nodule, the maximum diameter of which was ≤10 mm, with or without ulceration. | Continue the medication. Strengthen local treatment for patients with ulceration and bleeding to prevent infection. |
| 2 | Multiple or single nodule, diameter >10 mm, with or without ulceration. | Continue the medication. Observe, or give local treatment measures such as laser or surgical resection. Enhance local treatment for ulcer bleeding to prevent infection. |
| 3 | Multiple nodules were distributed all over the body, complicated with skin infection. | Suspend the drug, and resume it after relief to grade ≤1. Observe the patient or give local treatments such as laser or surgical resection. Give anti-infective treatment to patients complicated with infection. |

Remarks:

- There have been no cases of grade 4 life-threatening disease or grade 5 death, so no classification has been made for them.

Because reactive cutaneous capillary endothelial proliferation is self-limiting, it may resolve on its own in some patients during treatment or after the discontinuation of treatment. Therefore, the patient may be observed without any treatment.

# Research procedures

We will do our best to ensure that the tests and steps required by the protocol are implemented as planned. Unplanned situations may come up that are beyond the control of the researcher, making them difficult to detect. Under these circumstances, the investigators should take all necessary measures to protect the safety and interests of the subjects. When the testing required by a protocol cannot be performed, the investigator should record the reasons. In addition, the research team should be notified of unexpected situations in a timely manner.

## Screening period

Subjects will sign an informed consent form before undergoing any screening procedures specified in the trial. If routine tumor imaging evaluation is performed before the signing of the informed consent, as long as these CT or MRI scans are taken within 21 days before the start of the trial drug (bone scan can be done within 42 days) and meet the imaging evaluation requirements of this trial, there is no need to repeat CT, MRI, or bone scans during the screening period. If routine laboratory examination or other evaluations are performed before the subject signs the informed consent form, as long as the screening period is ongoing, there is no need to repeat the examination or evaluation during the screening period.

Unless otherwise specified, the following information should be collected within 21 days before the first treatment:

- Written informed consent;
- Each subject’s ID number. All participants will be assigned a unique ID number in this trial: the different digits of this ID number reflect the ID number of the research center and the ID number of the subject in the same center. The subject ID number is ordered sequentially according to the order in which they signed the informed consent form (001, 002, 003, etc.);
- Demographic data;
- Tumor diagnosis, surgical history, treatment history, and radiotherapy history. Tumor diagnosis includes at least histological classification, histological grade, Lauren classification, clinical stage, and time of initial diagnosis;
- Other medical history, including history of drug allergy, diagnosis and treatment of other serious concomitant diseases, and history of tumors other than gastric cancer and gastroesophageal junction cancer;
- Medication history within 4 weeks before signing the informed consent;
- Collection of combined medication/treatment data: information on combined medication and concomitant treatment after providing informed consent until the end of the safety follow-up period. Once the subjects stop the trial treatment, only the combined medications and concomitant treatments used for the new or unresolved adverse events related to the trial treatment will be recorded.
- Thyroid function values (see the test flow chart for specific requirements);
- Virological markers (including HBV, HCV, and HIV markers; see the test flow chart for specific requirements);

Imaging results within 21 days before signing the informed consent. The data will be evaluated according to RECIST v1.1. The imaging examinations include chest CT and enhanced CT scans of the abdomen and pelvis (CT scan thickness ≤ 5 mm). If the patient is allergic to the contrast agent for enhanced CT, a plain CT scan of the chest + MRI scan of the abdomen and pelvis could be performed. Patients with suspected brain metastases will undergo cranial-enhanced MRI or enhanced CT to rule out brain metastases. A bone scan is required when bone metastasis is suspected clinically;

- AEs;
- Blood and/or tissue samples
- The meeting of the inclusion and exclusion criteria.

The following information will be collected within 7 days before the first treatment:

- Complete blood count: Leukocytes, neutrophils, lymphocytes, red blood cells, hemoglobin, and platelets;
- Blood biochemistry: TBil, direct bilirubin, ALT, AST, AKP, γ-GT, total protein, albumin, urea/urea nitrogen, creatinine, endogenous creatinine clearance rate, uric acid, fasting blood glucose, triglyceride ester, cholesterol, potassium, sodium, chloride, calcium, and phosphorus;
- AFP;
- Blood amylase: When blood amylase is abnormal to a clinically significant level, a blood lipase test should be performed;
- Urinalysis, including proteinuria, urine glucose, urine occult blood, urine red blood cells, and white blood cells. If two consecutive semiquantitative methods show a protein concentration ≥2%, a quantitative 24-hour proteinuria test must be done;
- Fecal occult blood test: if fecal occult blood is positive, the test must be done again. If the fecal occult blood is again positive, gastroscopy should be performed if the investigator deems it necessary;
- Coagulation function, including the INR, APTT, PT, and FIB;
- For female subjects who could get pregnant, serum human chorionic gonadotropin (HCG) within 72 h before the first medication should be detected;
- Myocardial enzymes: creatine kinase MB isoenzyme (CK-MB), troponin I (cTnI), and lactate dehydrogenase;
- Vital signs (still sitting for 5 minutes), including temperature, blood pressure, pulse and respiratory rate;
- Comprehensive physical examinations: general condition, head and face, skin, lymph node, eye, ear, nose and throat (ENT), oral cavity, respiratory system, cardiovascular system, abdomen, reproductive-urinary system, musculoskeletal, nervous system, and mental status;
- height and weight measurements;
- ECOG PS score (see Appendix 2 for details);
- 12-lead ECG (performed after sitting still for at least 5 minutes): heart rate, PR interval, and QTc interval;
- Echocardiography, including assessment of LVEF;
- Blood pressure monitoring (see the test flow chart for specific requirements);
- Combined medications/treatments;
- AEs;
- The meeting of the inclusion and exclusion criteria;
- If all the inclusion criteria are met and no exclusion criteria are met, the subject can be enrolled.

## Treatment period and end of treatment

For details of the medications, please refer to Section 3.2.1 Medication regimen.

During treatment, relevant physical and laboratory examinations will be performed. Information on AEs and combined medications will be collected. Information on drug distribution, return, and inventory will be recorded. The subjects’ diaries will be distributed, checked for completeness, and returned.

During the treatment period, the imaging examinations should be performed under the same conditions as the baseline examination (scanned layer thickness, use of contrast agent, etc.). During the treatment period, the imaging evaluation was performed once every 2 cycles and at the end of treatment; If treatment is discontinued during the treatment period for reasons other than radiological progression, imaging should be performed at the end of treatment, unless imaging examinations are performed within 28 days.

The following examinations must be completed before medication on d1 of each treatment cycle and at the end of treatment. If the visit examination planned before the first medication is performed during the screening period and within 3 days before the first medication, a repeat examination is not needed before the first-cycle medication:

- Complete blood counts: leukocytes, neutrophils, lymphocytes, red blood cells, hemoglobin, and platelets;
- Blood biochemistry: TBil, direct bilirubin, ALT, AST, AKP, γ-GT, total protein, albumin, urea/urea nitrogen, creatinine, endogenous creatinine clearance rate, uric acid, fasting blood glucose, triglyceride esters, cholesterol, potassium, sodium, chloride, calcium, and phosphorus;
- AFP;
- Urinalysis, including proteinuria, urine glucose, urine occult blood, urine red blood cells, and white blood cells. If two consecutive semiquantitative methods show a protein concentration ≥2%, a quantitative 24-hour proteinuria test must be performed;
- Fecal occult blood test: performed when necessary;
- Coagulation function, including the INR, APTT, PT, and FIB;
- For subjects who can get pregnant, a pregnancy test will be done when necessary during the treatment period.
- Myocardial enzymes, including creatine kinase MB isoenzyme (CK-MB), troponin I (cTnI), and lactate dehydrogenase, were measured when necessary;
- Vital signs (still sitting for 5 minutes), including temperature, blood pressure, pulse and respiratory rate;
- Physical examination: A targeted physical examination was performed when there were clinical indications.
- Height and weight measurements;
- ECOG PS score (see Appendix 2 for details);
- 12-lead ECG (performed after sitting still for at least 5 minutes): performed when necessary, including heart rate, PR interval and QTc interval;
- Echocardiography: performed when necessary, including assessment of LVEF;
- Blood and/or tissue sample collection
- Blood pressure monitoring: During the trial period, blood pressure monitoring was completed by the subjects themselves, and the data were recorded in the subjects’ diary cards. In the first two periods, blood pressure was measured at least three times a week. If the blood pressure was abnormal, blood pressure monitoring was performed every day. If the blood pressure was normal, blood pressure was measured at least two times a week after the second cycle; in addition, blood pressure measurements were performed at each follow-up visit (see the trial flow chart for specific requirements).
- AFP/EGFR/VEGFR2 biomarker detection;

For subjects receiving camrelizumab, in addition to the above inspections, the following inspections should also be received on d1 of each treatment cycle before medication and after treatment.

- Blood amylase: When blood amylase is abnormal and has clinical significance, a blood lipase test should be performed;
- Thyroid function test (check not required for C1D1);
- Myocardial enzymes, including CK-MB, cTnI, and lactate dehydrogenase;

## Follow-up period

After the end of the treatment visit, the follow-up will cover safety, tumor progression, and survival.

After the end of treatment, the combined medications and concomitant treatments used for new or unresolved AEs related to the trial treatment will be recorded. After the end of treatment, AEs will be recorded until the end of the safety follow-up period, and the AEs will be followed up until the end of the safety follow-up period or until the AEs to the baseline level or grade ≤1, the achievement of a stable state, or a reasonable explanation (such as loss to follow-up or death).

Safety follow-up:

Regardless of whether the subjects started new antitumor treatment, the subjects needed to return to the research center for safety follow-up or follow-up by telephone. If the subject cannot resume the study medication due to an AE and the date of the last medication is determined as the date of the last medication, if this date is more than 30 days (+7 days) from the time when the subject is determined to withdraw from the trial treatment, there is no need to record the protocol deviation.

Follow-up of the time to disease progression:

For subjects who still have no radiological progression at the end of treatment, the follow-up frequency during the treatment period should be maintained, and radiological evaluation should be performed every 6-8 weeks until radiological progression, receiving other antitumor treatments, withdrawal of informed consent, loss to follow-up, or death.

Survival follow-up:

After the end of treatment, the subjects will be followed up for survival every 3 months until the subject died or lost to follow-up, the sponsor terminated the study, or other end-of-study criteria were reached (whichever occurred first). The researchers may ask the subjects, their relatives, or local physicians by telephone to collect information on the survival status (date and cause of death) and other antitumor treatments after the end of the trial. The status of each survival follow-up will be recorded in detail in the original medical records.

## Unplanned visits

Before the end of the trial, if the subjects experience or need unplanned follow-up (such as the occurrence of AEs), the following items will be recorded:

- Date of visit;
- Reasons for visit;
- Combined medications/treatments (within the collection period specified in the protocol);
- Information about AEs (within the collection and follow-up period specified in the protocol);
- Relevant examinations (including imaging examinations, if any);
- Whether the subject could continue or resume the study treatment, and if so, the administered dose.

# Evaluation

## Efficacy evaluation and analysis

This trial used RECIST v1.1 to evaluate tumor remission in the subjects.

The tumor assessment during the screening period (baseline) included chest CT and enhanced CT scans of the abdomen and pelvis (CT scan thickness ≤ 5 mm). If subjects are allergic to the contrast agent of enhanced CT, plain chest CT scan + MRI scan of the abdomen and pelvis can be performed. Subjects with suspected brain metastases also must undergo craniocerebral-enhanced MRI or enhanced CT to rule out brain metastases. Bone scans are required for subjects with definite or clinically suspected bone metastasis (subjects who will undergo bone scan within 42 days before signing the informed consent can be waived). Tumor assessment at other locations can be performed if clinical manifestation is identified.

Rules for the selection of target lesions: as long as each organ has no more than two target lesions and the total number of target lesions does not exceed five, as many involved organs should be covered as possible. At baseline, the number of target lesions, location, long axis of each target lesion (excluding the lymph nodes), short axis of the lymph node lesions, and sum of the diameters of all lesions are recorded. Due to the difference in measurement caused by the different degrees of filling of the stomach, the gastric lesions cannot be selected as the target lesions.

The baseline evaluation and the posttreatment efficacy evaluation should use the same method, and the same investigator should try to do all the evaluations:

- At the end of every 6-8 weeks during treatment and at the end of treatment;
- If the subjects have unconfirmed disease progression at the end of treatment, imaging examination will be conducted before discharge from the trial (if the previous examination is within 4 weeks after discharge from the trial, there is no need to undergo another examination at the end of treatment).
- To study the tumor progression and survival, for subjects who still have no radiological progression at the end of treatment, the follow-up interval during the treatment period should be maintained, and imaging assessments should be performed every 6-8 weeks until radiological progression, the start of other antitumor treatments, withdrawal of informed consent, loss to follow-up, or death.

Tumor evaluation during treatment includes chest CT, enhanced CT scan of the abdomen and pelvis (CT scan thickness ≤ 5 mm); if there is an allergy to the contrast agent on enhanced CT, plain CT scan of the chest + contrast-enhanced MRI scan of the abdomen and pelvis are performed; cranial CT scan/MRI and bone scan are performed only with clinical indications. Each tumor assessment must cover all target lesions and all nontarget lesions.

When clinically indicated, the investigators may perform unplanned tumor assessments. During the entire trial period, each subject will undergo the same imaging examinations. Before the medication of the next cycle, the investigator will review the results.

Once clinical progression occurs, physical examination and imaging confirmation are required immediately at any time instead of waiting for the next planned imaging examination. If this unscheduled imaging examination reveals clinical progression that is not in line with the disease progression defined in RECIST v 1.1, the follow-up examination should still include the next imaging examination on the originally planned date, unless the next planned examination is less than 14 days after this examination.

## Safety evaluation

### Adverse events

The evaluation of AEs included the type, incidence, severity (graded according to NCI-CTCAE v5.0), occurrence and end time and whether they are severe AEs (SAEs) and their correlation and outcomes.

### Pregnancy test

For female subjects who can get pregnant, a serum pregnancy test will be performed within 72 h before the start of medication. If they have a negative pregnancy test during the screening period, appropriate contraceptive measures should be started. If the hCG test is positive, the subject should withdraw from the trial.

### ECOG PS score

The ECOG PS performance status scoring criteria.

# Adverse event report

## Adverse events (AEs)

### Definition of AEs

An AE is an adverse medical event that occurs after the clinical trial subject provides informed consent. It can be any unfavorable unexpected symptoms, signs, abnormal laboratory tests, or diseases but does not necessarily have a causal relationship with the treatment.

This trial will collect information on AEs that arise from the time the subjects sign the informed consent form to the end of the safety follow-up period. AEs include at least the following situations:

1) Deterioration of a preexisting (before entering the clinical trial) medical condition/disease, including the aggravation of symptoms, signs, and abnormal laboratory tests, after starting a trial drug;

2) Any new AEs (including symptoms, signs, and newly diagnosed diseases);

3) Abnormal clinically significant laboratory test values or results.

Diagnostic or therapeutic invasive (such as surgery) and noninvasive procedures should not be reported as AEs. When the disease condition that leads to the operation meets the definition of an AE, it should be reported. For example, acute appendicitis that occurs during the reporting period of an AE should be reported as an AE, and appendectomy should be recorded as the treatment for this AE.

The researchers will record in detail any AEs that occur to the subjects, including the name of the AE, time of occurrence and end, severity (grading is in accordance with NCI CTCAE v5.0), the link between the AE and any trial drug, its duration, its causes, measures taken with respect to the trial medication as a result of the AE, the outcome of the AEs, and whether it is serious.

### Criteria for determining the severity of AEs

The grading criteria for AEs are will follow NCI-CTCAE version 5.0. The following criteria should be applied for the AEs not listed in the NCI-CTCAE version 5.0 table:

| **Grade** | **Clinical description of severity** |
| --- | --- |
| 1 | Mild. No clinical symptoms or mild clinical symptoms; only clinical or laboratory test abnormalities; no treatment required |
| 2 | Moderate. Minor, local, or noninvasive treatment is needed; age-matched activities of daily living (ADLs) using tools, such as cooking, shopping, making phone calls, counting money, etc. are limited. |
| 3 | Severe. The patient’s condition is medically serious but not life-threatening for the time being; the patient is hospitalized or disabled from it. The self-care ADLs are limited, such as bathing, dressing, undressing, eating, going to the bathroom, taking medication, etc. |
| 4 | Life-threatening. Urgent treatment indicated |
| 5 | AEs resulting in death |

Attention should be paid to the severity of AEs. For example, “severe headache” may be classified as severe to some degree, but it should not be classified as a SAE unless it meets the criteria for SAEs.

### Criteria for determining the relationship between AEs and trial drugs

Investigators should comprehensively determine the relationship between AEs and investigational drugs, such as whether the occurrence of AEs and the medication are in a reasonable chronological order, the characteristics of the trial drugs, the toxicological and pharmacological effects of the trial drugs, whether the subjects used other combined drugs, the severity of the AEs, the underlying diseases, medical history, family history, and dechallenge and rechallenge responses. The possible relationship between AEs and the trial drugs is evaluated according to the five-level classification method of “definitely related, possibly related, possibly irrelevant, definitely irrelevant, and undeterminable”.

## SAEs

### Definition of SAEs

SAEs are medical events that require hospitalization or prolong hospitalization, cause disability, hinder the ability to work, threaten death, and cause congenital malformations, etc., that occur during a clinical trial. AEs that meet one or more of the following criteria are considered SAEs:

- Events that led to death;
- Life-threatening events (the term “life-threatening” means that the subject is at risk of death at the time of the event/reaction; it does not mean that death is possible only after the event/reaction worsens);
- Events requiring hospitalization or prolonged hospitalization;
- Events that can lead to permanent or severe disability/incomplete function/affect the ability to work;
- Congenital anomalies or birth defects;
- Other important medical events/reactions that are not immediately life-threatening and will not lead to hospitalization but, according to reasonable medical and scientific judgment, may cause harm to the subject or may require intervention (such as drugs or surgery) to prevent the serious consequences listed above.

### Hospitalization

In clinical trials, adverse events that lead to hospitalization (even if it is less than 24 hours) or prolonged hospitalization are considered SAEs.

However, hospitalization or prolongation of hospitalization due to the following situations does not need to be reported as an SAE:

1. Rehabilitation institution admission

2. Nursing home admission

3. Routine emergency room admission (less than 24 hours)

4. Same-day surgery (such as outpatient/same-day/ambulatory surgery)

5. Social reasons (medical insurance reimbursement, etc.)

Hospitalization or prolonged hospitalization that is not related to an AE is not an SAE. Such hospitalization may include but is not limited to the following situations:

1. The patient is admitted to the hospital due to preexisting disease, not a new AE or aggravation of existing disease (e.g., abnormal laboratory test results that persisted before the trial);
2. Hospitalization for management reasons (e.g., annual routine physical examination);
3. Hospitalization as stipulated in the trial protocol during the clinical trial (e.g., operating in accordance with the requirements of the trial protocol);
4. Elective hospitalization (such as elective surgery) that is not related to AEs;
5. Scheduled treatments or surgical procedures should be recorded in the entire trial protocol and/or the individual baseline information of the subjects;
6. The patient is admitted to the hospital due to the use of blood products only.

### Disease progression and death

Disease progression is defined as the deterioration of the condition caused by the indications of the trial, including the radiological progression or the progression of clinical symptoms or signs. New metastatic lesions of the primary tumor or the progression of the original metastatic lesions are considered disease progression. Life-threatening events that require hospitalization or prolonged hospitalization or events that resulted in permanent or severe disability/incompetence/affecting work ability, congenital anomalies, or birth defects are not reported as SAEs due to symptoms and signs of disease progression. If there is any uncertainty about whether an SAE is due to disease progression, it should be reported as an SAE.

In the population in this study, disease progression is expected, so the term “disease progression” will not be referred to as an AE. When disease progression occurs, the events classified as disease progression should be reported as AEs. For example, if a subject develops epilepsy that is determined to be related to brain metastasis, the AE item should be recorded as “epilepsy” rather than “disease progression” or “brain metastasis”.

Deaths assessed by the investigator as possibly due to the signs and symptoms of disease progression should be reported as SAEs. The word “death” should not be used as the AE or SAE term; rather, the result of the event and the event causing or leading to death should be recorded as the AE or SAE. If the cause of death is unknown and cannot be determined at the time of reporting, the AE or SAE is recorded as “death from unknown causes”.

### Other new antitumor treatments

If the subjects start to receive other new antitumor treatments before the end of the safety follow-up period, for nondeath AEs that are suspected to be unrelated to the trial drugs, the reporting period is up to the start of new antitumor treatment. If death occur during the safety follow-up period, whether or not the subject has received other treatments, death must be reported promptly as a SAE.

### Abnormal liver function test

If an abnormal aspartate transaminase (AST) and/or alanine transaminase (ALT) level is accompanied by an abnormally elevated total bilirubin level after receiving a study treatment, the conditions [1][2][3] below are met, and there is no other cause of the abnormality, this condition should always be treated as an important medical event that needs to be reported according to the SAE procedure.

| Condition | Criteria |
| --- | --- |
| [1] Abnormal AST or ALT | ALT or AST ≥ 3 × ULN (if normal at baseline);  ALT or AST ≥ 2 × baseline level and≥ 3 × ULN; or ≥ 8 × ULN (if abnormally elevated at baseline). |
| [2] Abnormal TBIL | TBIL > 2 × ULN (if normal at baseline);  Increase in TBIL ≥ 1×ULN or its value > 3×ULN (if abnormally elevated at baseline). |
| [3] No hemolysis and alkaline phosphatase < 2 × ULN (or no ALP information obtained) | |

Note: ULN (upper normal value)

If the subjects have abnormal AST and/or ALT levels combined with abnormally increased total bilirubin levels during the safety follow-up period, they should come to the research center for evaluation as soon as possible (preferably within 48 hours of learning of the abnormal result).

### Reporting of SAEs

During the trial, if the occurrence of a SAE, whether it is the first report or the follow-up report, the investigator must immediately fill in the "Serious Adverse Event (SAE) Reporting Form", sign and date it, and immediately report to relevant units within 24 hours of being informed of the SAE regulations.

If SAEs occur after starting to use the trial drug, they should be reported to Hengrui promptly. Hengrui’s email address for the report of SAEs in this project is hengrui_drug_safety@hrglobe.cn.

SAEs, including their symptoms, severity (according to the NCI-CTC5.0 grading system), association with each trial drug, time of occurrence, treatment time, measures taken for each trial drug due to SAE, time and method of follow-up, and outcome, should be recorded in detail. If the investigator believes that an SAE is not related to the trial drug but is potentially related to the trial conditions (such as termination of the original treatment or comorbidities during the trial), this relationship should be described in detail in the narrative section of the SAE report form. If the intensity of an ongoing SAE or its relationship with the trial drug changes, the SAE should be reported immediately. If the investigator believes that a reported SAEs has been misreported, the investigator can correct, withdraw, or downgrade the report in a follow-up report and then report it according to the SAE reporting procedures.

## Collection and follow-up period of AEs/SAEs

The AE/SAE collection period of this trial will start from the time when the subjects sign informed consent to the end of the safety follow-up period. If a patient dies during the safety follow-up period, it is reported as an SAE. After the safety follow-up period, the investigators only needed to report the SAEs related to the trial drug. AEs/SAEs should be followed up until they disappear, recover to the baseline level or to grade ≤1, reach a stable state, or are reasonably explained (such as loss to follow-up or death). The best outcome and a clear drug-related determination should be obtained as much as possible. At each visit, the investigators should ask about the AEs/SAEs that occur after the previous visit and provide follow-up information in a timely manner according to the queries received.

## Pregnancy

If a female subject becomes pregnant during the clinical trial, she should immediately stop using the trial drug and be removed from the group. During the clinical trial, if the female partner of a male subject gets pregnant, the subject can continue with the study. The investigator should fill in the "Hengrui Clinical Research Pregnancy Report/Follow-up Form" within 24 hours of learning about the pregnancy event, report it to the sponsor and report the ethics in a timely manner. If a female participant gets pregnant between starting a trial drug and the end of the safety follow-up period, an investigator should fill out the “Hengrui Clinical Trial Pregnancy Report/Follow-up Form” file it with Hengrui within 15 days after the investigator becomes aware of the pregnancy.

The investigators fellow up on the pregnancy results until 1 month after delivery. The results must be reported to the sponsor and Hengrui. Pregnancy results such as stillbirth, spontaneous abortion, and fetal malformation are considered SAEs that are reported according to the time limit for SAEs. If the subject has a SAE while pregnant, the investigator should also complete the "Reporting Form of Serious Adverse Events" and follow the reporting procedure for SAEs.

## Reporting of nonserious AEs and other special conditions

If the investigator determines that the relationship between an AE and the trial drug is “definitely related”, “possibly related” or “undeterminable”, that AE is considered due to the trial drug*.* The investigator should report the monthly new/updated nonserious adverse drug reaction information to Hengrui in the form of the “Nonserious adverse drug reaction (NSADR) list”. Other special circumstances may not meet the definition of AEs, but the data still need to be collected to meet the requirements of the regulations. Special circumstances include but are not limited to medication errors, drug misuse, drug abuse, and occupational exposure.

# Trial management

## Ethics standards and informed consent

This clinical trial complies with the Declaration of Helsinki (1996 update), the Good Clinical Trials (GCP) guidelines promulgated by the China National Medical Products Administration (NMPA), and relevant laws and regulations. Approval from the ethics committee must be obtained before the start of the trial. Any modifications to the protocol during the trial will be reported to the ethics committee, and approval will be obtained before proceeding. All clinical researchers will follow all applicable rules and regulations to protect the subjects. The informed consent forms used in the informed consent process must be approved by the ethical review board and available for inspection.

The clinical investigator must explain to the participants that participation in the clinical trial is voluntary and that the subjects have the right to withdraw from the trial at any time without being discriminated or retaliated against; their medical treatment and rights and interests remain unaffected, and they can still continue to receive other forms of treatment. The subjects must be made aware that the personal data of the participants in the trial and the trial will be kept confidential. In addition, subjects should be informed of the nature, purpose, possible expected benefits, and possible risks and inconveniences of the clinical trial, as well as of the other treatment methods available and the rights of the subjects under the Declaration of Helsinki. This will give the subjects enough time to consider whether they are willing to participate in the trial and to sign the informed consent form.

Before performing any procedure required by the protocol, the subjects must:

- Be informed of the contents of the study and all the contents and terms of the informed consent form.
- Have enough time to ask questions and consider whether to participate in the study.
- Voluntarily agree to participate in the research.
- Sign and date the informed consent forms approved by the institutional review board (IRB)/independent ethics committee (IEC).

Any major changes in the study will require revisions to the protocol. Unless an obvious direct harm to the subjects must be handled, the investigator should not make any changes to the study without the approval of the IRB/IEC and the sponsor. Changes to the protocol to eliminate obvious direct risks to the subjects can be implemented immediately, but the changes must be recorded in the protocol modification, reported to the IRB/IEC, and submitted to the appropriate regulatory agency within the required deadline. The review and approval process of the original plan must be the same as the review and approval process of the original plan.

## Protocol revision

This “Clinical Trial Protocol” and “Clinical Trial Case Report Form” are developed by the principal investigator and implemented after approval by the Ethics Committee of our hospital. During the clinical trial, any modification to the trial protocol should be considered by the investigator and approved by the ethics committee.

## Quality assurance of clinical trials

To ensure the quality of the clinical trial, before the start of the formal trial, the principal investigators will discuss and formulate the clinical research plan. All researchers who will participate in the clinical trial will be trained for it.

The drugs used in clinical trials must be managed according to the standard operating procedures, including the drug supply, storage, distribution, return, and destruction.

According to the GCP guidelines, necessary steps should be taken during the design and implementation stages of the study to ensure that the collected data are accurate, consistent, complete, and reliable. All observed results and abnormal findings in clinical trials should be carefully verified and recorded in a timely manner to ensure data reliability. All instruments, equipment, reagents, and standard products used in the various inspection in clinical trials should have strict quality standards and be sure to work under normal conditions.

## Data management

The purpose of data management is to ensure the reliability, completeness, and accuracy of the data. The goal is to obtain high-quality real data for statistical analysis. This study will use an electronic case report form (eCRF) to collect and manage the trial data.

### Data collection

This trial uses eCRFs to collect research data. The electronic data capture (EDC) system training will be provided to designated research institution personnel, and the research institution personnel can only log in to the EDC system after receiving the training. The lead investigator or dedicated data entry personnel (clinical research coordinator should enter data into the EDC system in accordance with the requirements of the visit process and the guidelines for filling out the eCRF. The system logic verification program will perform completeness and logic checks of the clinical trial data on the data entered in the EDC system and generate error messages for the problematic data, allowing the lead investigator or clinical research coordinator to modify or explain the problematic data. After the database is locked, the investigator will receive a CD-ROM or file copy of the subject’s data for archiving at the research institution.

Investigators are ultimately responsible for the collection and reporting of all clinical data and laboratory data recorded on the eCRF and other data collection forms (original records) to ensure attributability, legibility, timeliness, originality, accuracy, durability, completeness, and consistency. The eCRF must be signed by the investigator or relevant authorized person to confirm that the data recorded in the eCRF are real. Any data amendment in an eCRF or original record must be dated and signed, and necessary explanations should be given, but the previous original records cannot be obscured.

Original records are hospital medical records and examination reports. Data collected in the eCRFs must be consistent with these original data. In some cases, the eCRF can also be used as the original record. At this time, research institutions need to have relevant documents to clarify which data will be recorded in the eCRF and use the eCRF as the original record.

### Data management and quality control

To ensure the authenticity and reliability of the clinical trial data and improve the quality of the clinical data, the clinical monitor appointed by the investigators will check the completeness, consistency, and accuracy of the trial data in the clinical database in accordance with the standard operating procedures. The personnel of the research institution will be guided to make necessary additions or corrections to the problematic data.

Data review investigators must keep the original documents of each subject participating in the trial, including study medical records and visit records (inpatient or outpatient medical records), which include demographic indicators, medical information, laboratory data, ECGs, and any other examinations and results of any evaluations. All information on the eCRFs must be from the original documents in the subject’s file. The researchers must also save all signed informed consent forms.

### Maintenance of trial records

The investigators/institutions agree to save relevant records for 5 years after the end of the clinical trial, including the identification numbers of all involved subjects (enough information will be linked to the records, such as the eCRF and the hospital) and all originally signed informed consent medical records, copies of all eCRFs, safety report forms, original records, detailed records of treatment, and relevant communication documents (such as letters, meeting records, and telephone reports). Investigators/institutions should preserve and record the trial data in accordance with the relevant specifications.

# Data analysis/Statistical methods

The statistical analysis plan will provide all the contents of the analysis and the expression of the results, which will be kept by the investigator. Appropriate modifications can be made in the statistical analysis plan for the plan marked in the scenario. Any important revisions to the definition and analysis of the primary endpoints should be reflected in the protocol amendments.

## Analysis Population

- Full analysis set (FAS): All subjects who sign the informed consent and receive at least one dose of any study treatment are included in the FAS.
- Per-protocol set (PPS): The PPS is a subset of FAS, defined as the subjects who have no major protocol deviations during the trial, which may have significant impact on study treatment efficacy.
- Safety set (SS): the subjects who are enrolled and receive at least one dose of any study treatment.
- The efficacy evaluation of this study will be performed in FAS and PPS, with FAS as the primary analysis set. Safety analysis will be done in SS.

## Statistical methods

### General method

Unless otherwise stated, all data will be analyzed by the intervention groups defined in section 3.2.1 using appropriate statistics according to the data type: mean ± standard deviation or median (minimum, maximum) will be calculated for the continuous data, while the categrical data will be summarized using frequency and the corresponding percentages, given as n (%). The Kaplan‒Meier method will be used for analyzing time-to-event data to estimate the median survival time, and the corresponding 95% confidence intervals will be calculated based on Brookmeyer-Crowley method.. If necessary, Kaplan-Meier curves will be also presented.

### Subject disposition

Subject disposition information, including but not limited to the number of subjects being enrolled, treated, , withdrawing from the trial, and major protocol deviations occurring in patients, will be summarized. The reasons for screening failure and the reasons for withdrawal from the trial will also be summarized.

### Demographics and baseline characteristics

The demographic characteristics including age(years), sex, and so on, as well as other baseline clinical characteristics including tumor history, previous medical history, will be summarized by descriptive statistics.

### Primary Efficacy Analysis

In this study, the primary endpoints are the objective response rate (ORR) in each intervention group. . The confirmed ORR will be analyzed mainly based on FAS for each intervention group. The 95% confidence interval of ORR will be calculated using the Clopper–Pearson method based on binomial distribution.

Subgroup analysis of ORR will be performed for age, sex, primary tumor location, metastatic site, and AFP level, ORR as well as the corresponding 95% Clopper-Pearson CI will be calculated for each subgroup.

### Secondary Efficacy Analysis

For time-to-event data of the secondary endpoints, Kaplan‒Meier method will be adopted to estimate median time to event of PFS, DoR, TTR and OS, and theBrookmeyer–Crowley method will be used to estimate the corresponding 95% confidence intervals. The Kaplan-Meier curves will also be presented. The analysis will be performed mainly based on FAS. Subgroup analysis will be performed for age, sex, primary tumor location, metastatic site, and AFP level.

The statistical analysis methods used for the disease control rate are the same as those being used for ORR.

### Safety Analysis

All the safety analysis will be performed in SS for each intervention group.

#### Analysis of Adverse Events

AEs are coded as low-level terms, preferred terms, and major system–organ–class (SOC) using MedDRA (v20.0 or higher).

Treatment-emergent adverse events are defined as any AEs that occur after starting the use of the trial drug. The incidence of AEs, SAEs, AEs of grade ≥3, drug-related AEs, drug-related SAEs, AEs that lead to dosage adjustments, AEs that lead to termination of therapy, and surgery-related AEs will be summarized for each intervention group. They will be further summarized by preferred term (PT) and severity. In addition, AEs with an incidence of ≥5%, as well as SAEs with an incidence of ≥5% will also be summarized by PT and severity. If applicable, the Kaplan‒Meier method will be used to analyze the time to AE occurrence and duration of AE for AEs of special concern.

#### Laboratory examination and analysis

The results of laboratory examinations (such as complete blood count and urine biochemistry tests) will be evaluated appropriately. Abnormal laboratory test values will be determined taking normal range as reference (higher or lower). The baseline and worst postbaseline clinical abnormalities of the laboratory examination parameters will also be summarized. The baseline and highest postbaseline CTCAE grade laboratory test(s) will be summarized if NCI CTCAE grading is applicable.

### Exploratory endpoint analysis:

If applicable, the exploratory analysis will be perform mainly in a descriptive way. If applicable, the Cox proportional-hazards model or logistic regression will be used for multivariate analysis to explore the correlation between each biomarker and treatment efficacy according to the efficacy analysis endpoint type.

### Previous and concomitant medications

The concomitant medications will be coded using the WHO Drug Dictionary. The previous and concomitant medications will be summarized and listed according to the anatomical therapeutic chemical (ATC) classification. Previous medication is defined as drugs used stopped before the first administration of the study treatment. Concomitant medication is defined as drugs except for the study treatment being used after first administration of the study treatment. Previous and combined medications will be listed.

# Data management methods

## Data recording

This trial will use an EDC system to collect and manage clinical research data.

### Filling in of original medical records and records

The original medical records and records will be completely preserved as the original documents of the clinical trial. The investigator is responsible for filling in and keeping the original medical records. The subject information on the cover of the medical records will be checked before filling it in. The handwriting must be neat and legible so it is convenient for the sponsor’s monitor to perform data verification with the eCRF.

### Fill-in eCRFs

The data in the eCRFs come from original documents such as study medical records and laboratory examination reports and should be consistent with the original documents. Any observation or inspection results during the test should be completed in the eCRF in a timely, correct, complete, standardized and truthful manner.

When making data corrections to the eCRF, you need to fill in the reason for the data modification according to the system prompts.

### eCRF audit

The investigator should complete, save, and submit the eCRF for each subject soon after the visit. The system logic checks will check the completeness and logic of the data entered in the EDC system and question the problematic data, allowing the researcher or data entry personnel to modify or explain the problematic data. If necessary, this will be done until the problematic data are resolved. The monitors, data administrators, and medical reviewers will also review the eCRF data and question doubtful data when necessary. Researchers should promptly respond to queries from system and data review personnel. After the data cleaning is completed, the PI issues an electronic signature on the completed eCRF.

## Data management

### Establishing EDC database

The data administrator establishes the research data acquisition system and database according to the trial protocol, and subjects can use it online before they enroll in the trial. All EDC users need to complete relevant training and fill in the training records and account application form to obtain the corresponding account number to log in to the system.

### Data auditing and database locking

Before the database is locked, all the deviations from the protocol that occur during the trial should be compiled, and a data review meeting should be convened. All decisions made in the data review meeting will be recorded in documents.

After all the data have been approved, the database will be locked after confirmation by the researchers and statistical analysts, after which the data files can no longer be changed. The locked data should be properly preserved for future reference.

### Data archiving

After the completion of the study, the EDC system will generate each subject’s eCRF in PDF format, save it on the CD-ROMs, and hand it to the sponsor and each institution for retention of records. The storage and management of the test data must follow the requirements of the GCP.

# Raw data and raw documents

In accordance with the International Council for Harmonization (ICH) E6, relevant regulations and research institutions' protection requirements for subjects' personal information, each research center must properly maintain the treatment and scientific records related to this trial. As part of the trials funded or participated in by Hengrui, each research center should allow Hengrui or its authorized representatives and regulatory agencies to inspect (or copy, if permitted by law) clinical records for quality review, audit, assessment of safety, research progress, and data validity.

Raw data are all the information necessary for the reconstruction and evaluation of clinical trial and are the original records of clinical discoveries, observations, or other activities. Examples of these original files and data records include but are not limited to hospital records, laboratory records, memorandums, subject diary cards, pharmacy dispensing records, recordings of consultation meetings, data recorded by automated instruments, photocopied or transcribed records that are verified as accurate and complete, microfilm, photographic negative, microfilm or diskettes, X-ray films, and the documents and records of the subjects kept in the pharmacies, laboratories, and medical technology departments participating in the trial.

# Quality Assurance and quality Control

To ensure the quality of the trial, before the official start of the trial, the investigators have developed a clinical trial plan. Whether the researchers who participated in the trial had received appropriate GCP training will be confirmed.

Each research center must manage the trial medications according to standard operating procedures, including reception, storage, distribution, return, and destruction (if applicable).

According to the GCP guiding principles, necessary steps should be taken during the design and implementation stages of the trial to ensure that the collected data are accurate, consistent, complete, and credible. All observed results and abnormal findings in the clinical trial should be verified and recorded in a timely manner to ensure data reliability. The instruments, equipment, reagents, and standards used in the various inspection items in clinical trials should have strict quality standards and ensure that they are working normally.

The drug regulatory department, IRB/IEC, and auditors may conduct systematic inspections of the activities and documents related to the clinical trial to evaluate whether the trial is conducted in accordance with the requirements of the trial protocol, standard operating procedures, and relevant laws and regulations and whether the trial data are timely, authentic, accurate, and complete. The audit should be performed by personnel who are not directly involved in the clinical trial.

# Regulatory ethics, informed consent and protection of subjects

## Regulatory considerations

This study will fully comply with the ICH E6 GCP guidelines and the Declaration of Helsinki or the laws and regulations of the country where the trial is conducted, whichever provides greater protection to the individual. This study will meet the requirements of the ICH E2A guidelines (Clinical Safety Data Management: Definition and Standards for Expedited Reporting).

The legal basis for the design of the scheme is as follows:

1) Measures for the Administration of Drug Registration

2) Good Clinical Practice for Drugs

3) Technical Guiding for Clinical Pharmacokinetic Studies of Chemical Drugs

4) Consensus on ethical principles based on international ethical guidelines, including the Declaration of Helsinki and the international ethical guidelines of the Committee for International Organizations in Medical Sciences (CIOMS)

5) ICH guidelines

6) Other applicable laws and regulations

## Ethical norms

The investigators will ensure that the trial fully complies with the requirements for the protection of subjects stipulated in 45 CFR Part 46, 21 CFR Part 50, 21 CFR Part 56, and/or ICH E6.

Without agreement by both the trial sponsor and the investigator, neither party will unilaterally modify the trial protocol. To eliminate direct and immediate harm to the subjects, the investigator may change or deviate from the trial protocol before obtaining approval from the ethics committee/institutional review board. At the same time, the deviations or changes and the reasons thereof, as well as the proposed amendments to the protocol, should be submitted to the ethics committee/institutional review board for consideration as soon as possible. The investigator must explain and record any deviations from the protocol.

During the clinical trial, any modification to the trial protocol is submitted to the ethics committee. If necessary, corresponding modifications to other trial documents should be made and submitted and/or approved in accordance with the requirements of the ethics committee. The investigator is responsible for regularly submitting the interim report/annual review report according to the relevant requirements of the ethics committee, and after the end of the trial, the investigator should notify the ethics committee that the trial is over.

## Independent ethics committee

The protocol, informed consent form, recruitment materials, and materials of all subjects will be submitted to the ethics committee for review and approval. The subjects can be enrolled after the protocol is approved, and informed consent must be obtained. Any amendments to the protocol must be reviewed and approved by the ethics committee before implementation. All amendments to the informed consent form must also be approved by the ethics committee, and the ethics committee should decide whether to sign the new version again for the subjects who have signed the previous version of the informed consent form.

## Informed consent

### Informed consent and other written information required by the subjects

The informed consent form describes the medications and trial processes in detail and fully explained the risks of the trial to the subjects. Before subjects perform any research-related procedures, written informed consent must be obtained.

### Informed consent process and records

Informed consent is obtained before the subjects agree to participate in the clinical trial, and informed consent is maintained during the whole process of clinical trial. The risks and possible benefits of participating in the trial will be discussed in detail with the participants or their legal representatives. The subjects will be required to read and review the informed consent form approved by the ethics committee. The investigator will explain the clinical trial to the subjects and answer any questions the subjects may have. The subjects could only participate in the trial after signing the informed consent. During the whole process of clinical trial, subjects can withdraw their consent at any time. A copy of the informed consent will be retained by the subjects. Even if the recruited subjects refuse to participate in this trial, their rights and interests will be completely protected, and the quality of their medical care will not be affected.

## Confidentiality of subject information

The confidentiality of subject information is strictly implemented by the investigators and participating researchers. Confidentiality covers both biological samples and genetic tests in addition to the clinical information of the subjects. Therefore, the study protocol, documents, data, and all other information generated therefrom will be strictly confidential.

Other authorized representatives of the sponsor, IRBs, regulatory departments, and representatives of the pharmaceutical company that provide the trial drugs may inspect all the files and records that the investigator is required to maintain, including but not limited to medical records and the medication records of the subjects. The research center should allow access to these records.

The contact information of the subjects will be securely stored at each research center and will be used only for internal use during the trial process. At the end of the study, all records will continue to be stored in a secure place for the time frame defined by local IRBs and regulations.

The trial data of subjects collected for statistical analysis and scientific reports will not include the contact information or identifying information. Instead, individual subjects and their study data will have unique identification numbers. The trial data entry and trial management systems used by the researchers of each clinical research center are confidential and password-protected. At the end of the trial, the identifying information of all research databases will be deidentified and will be archived at each clinical research center.

# Publication of trial results

The trial results are obtained from the sponsors and funders. If the investigator plans to publish any information related to the trial, the investigator should provide the sponsor and funders with the original manuscripts, abstracts, or full texts of all planned publications (posters, invited presentations, or guest lectures) at least 30 days before the submission of the document for publication or other form of release.

In compliance with standard editorial publication and ethical norms, this study supports the publication of the results of the multicenter study as a whole. After the overall trial results are published, researchers at other participating centers may negotiate with the leading unit/team leader’s unit on the publication of individual results for each subcenter/subcenter region.

The rules of authorship in publications will be determined through discussion between the researchers.

# Clinical trial progress

Time to enroll in the first study: September 2020

Enrollment time of the last subject: March 2023

End of the study: March 2024

# References

[1]. Al-Batran S-E, Hofheinz RD, Pauligk C, et al: Histopathological regression after neoadjuvant docetaxel, oxaliplatin, fluorouracil, and leucovorin versus epirubicin, cisplatin, and fluorouracil or capecitabine in patients with resectable gastric or gastro-oesophageal junction adenocarcinoma (FLOT4-AIO): results from the phase 2 part of a multicentre, open-label, randomised phase 2/3 trial. The Lancet Oncology 2016, 17(12):1697-1708.

[2]. Chen W, Sun K, Zheng R, et al.: Report of Cancer Incidence and Mortality in Different Areas of China, 2014. China Cancer 2018, 27(1):1-14.

[3] Chun H, Kwon SJ: Clinicopathological characteristics of alpha-fetoprotein-producing gastric cancer. J Gastric Cancer 2011, 11(1):23-30.

[4]. Lunghi A, Petreni P, Romanelli RG, et al. : Aggressive Gastric Carcinoma Producing Alpha-Fetoprotein: A Case Report and Review of the Literature. Case Reports in Oncology 2014, 7(1):92-96.

[5] Wang Y, Shen L, Lu M, et al. Multimodality Treatment Including Triplet Regimen as First-Line Chemotherapy May Improve Prognosis of Serum AFP-Elevated Gastric Cancer with Liver Metastasis. Gastroenterol Res Pract 2017, 2017:5080361-5080361.

[6]. Takahashi Y, Ohta T, Mai M: Angiogenesis of AFP producing gastric carcinoma: correlation with frequent liver metastasis and its inhibition by anti-AFP antibody. Oncology reports 2004, 11(4):809-813.

[7] Chen J, Chen L, Yang L, et al. Clinical efficacy and safety of apatinib in treatment of alpha fetoprotein-positive advanced gastric cancer. Journal of Clinical Oncology 2017, 35(15_suppl):e15513-e15513.

[8]. Li N. Efficacy and safety of apatinib for the treatment of AFP-producing gastric cancer. Annals of Oncology 2019, 30:v309.

[9]. Wang N, Chen S, Liu D, et al. Therapeutic effect of small molecule targeting drug apatinib on gastric cancer and its role in prognosis and anti-infection mechanism. Saudi Journal of Biological Sciences 2020, 27(2):606-610.

[10]. Qin S, Li JJTCR: Apatinib: get better application in gastric cancer and other cancers. 2016 2016:S601-S603.

[11]. Zhu AX, Kang YK, Yen CJ, et al. Ramucirumab after sorafenib in patients with advanced hepatocellular carcinoma and increased alpha-fetoprotein concentrations (REACH-2): a randomized, double-blind, placebo-controlled, phase 3 trial. The Lancet Oncology 2019, 20(2):282-296.

[12] Figueroa-Protti L, Soto-Molinari R, Calderón-Osorno M, et al. Gastric Cancer in the Era of Immune Checkpoint Blockade. Journal of Oncology 2019, 2019:1079710.

[13] Meng W, Bai B, Bai Z, et al. The immunosuppression role of alpha-fetoprotein in human hepatocellular carcinoma. Discovery medicine 2016, 21(118):489-494.

[14] Wang X, Wang Q: Alpha-Fetoprotein and Hepatocellular Carcinoma Immunity. Can J Gastroenterol Hepatol 2018, 2018:9049252-9049252.

[15]. Chen DS, Mellman I: Oncology meets immunology: the cancer-immunity cycle. Immunity 2013, 39(1):1-10.

[16] Xu J, Zhang Y, Jia R, et al.: Anti-PD-1 Antibody SHR-1210 Combined with Apatinib for Advanced Hepatocellular Carcinoma, Gastric, or Esophagogastric Junction Cancer: An Open-label, Dose Escalation and Expansion Study. Clinical Cancer Research 2019, 25(2):515.

[17]. Shen L, Peng Z, Zhang YQ, et al.: Camrelizumab combined with capecitabine and oxaliplatin followed by camrelizumab and apatinib as first-line therapy for advanced or metastatic gastric or gastroesophageal junction cancer: Updated results from a multicenter. Journal of Clinical Oncology 2019, 37(15_suppl):4031-4031.

[18]. Zitvogel L, Kepp O, Kroemer G. Immune parameters affecting the efficacy of chemotherapeutic regimens. Nature reviews Clinical oncology 2011, 8(3):151-160.

[19]. Wang.YK, Shen L, Jiao, X, Zhang, XT, et al.: Predictive and prognostic value of serum AFP level and its dynamic changes in advanced gastric cancer patients with elevated serum AFP. World J Gastroenterol.2018, 24(2):266-273.
